# MiR-132-3p suppresses peritoneal fibrosis induced by peritoneal dialysis via targeting TGF-*β*1/Smad2/3 signaling pathway

**DOI:** 10.1371/journal.pone.0301540

**Published:** 2024-04-11

**Authors:** Yangyang Yin, Yuqi Yang, Yongqiang Zhang, Yu Shang, Qian Li, Jing Yuan

**Affiliations:** 1 School of Medicine, Guizhou University, Guiyang, Guizhou, China; 2 Department of Nephrology, Guizhou Provincial People’s Hospital, Guiyang, Guizhou, China; 3 Guizhou University of Traditional Chinese Medicine, Guiyang, Guizhou, China; Nagoya University Graduate School of Medicine, JAPAN

## Abstract

**Background:**

Peritoneal fibrosis (PF) is the main complication of peritoneal dialysis (PD) and the most common cause of cessation from PD. There is still no effective therapeutic approach to reserve PF. We aimed to investigate the role of miR-132-3p and underlying potential mechanisms in PF.

**Methods:**

A total of 18 Sprague-Dawley (SD) rats were divided randomly into three groups (n = 6): (i)Control group (ii)PF group (iii)PF+Losartan group; Rats in the PF group and PF+Losartan group received daily intraperitoneal injections of 3 mg/kg chlorhexidine for 14 days, and rats in the PF+Losartan group simultaneously received daily intraperitoneal injections of 2 mg/kg losartan for 14 days. The control group was injected with saline in the same volume. Met-5A cells were treated for 24h with TGF-*β*1 dissolved in recombinant buffered saline at a concentration of 10 ng/ml, meanwhile, PBS solution as a negative control. The human peritoneal solution was collected for the detection of miR-132-3p.

**Results:**

*In vivo*, SD rats were infused with chlorhexidine to establish PF model, and we found that miR-132-3p significantly decreased and the expressions of transforming growth factor-*β*1 (TGF-*β*1), and Smad2/3 were up-regulated in PF. *In vitro*, miR-132-3p mimics suppressed TGF-*β*1/Smad2/3 activity, whereas miR-132-3p inhibition activated the pathway. In human peritoneal solution, we found that the expression of miR-132-3p decreased in a time-dependent model and its effect became more pronounced with longer PD duration.

**Conclusion:**

MiR-132-3p ameliorated PF by suppressing TGF-*β*1/Smad2/3 activity, suggesting that miR-132-3p represented a potential therapeutic approach for PF.

## Introduction

Peritoneal dialysis (PD) is a common alternative mode of renal replacement therapy for end-stage renal disease (ESRD) patients [1, 2]. Long-term PD treatment can cause changes in peritoneal morphology and function, including the loss of the mesothelium, accumulation of extracellular matrix, vascular hyalinization, and angiogenesis, eventually leading to PF [3, 4]. PF is the most common cause of dropping out of PD treatment. Hence, it is urgent to find effective approaches to prevent and delay the progression of PF.

Transforming growth factor-*β*1 (TGF-*β*1) is a well-known key pro-fibrotic cytokine and is involved in the mesothelial-mesenchymal transition, which leads to PF development [5]. TGF-*β*1 activates its downstream signaling molecules, such as Smad 2 and Smad 3 proteins, which play a competitive role in profibrotic and antifibrotic actions, so as to regulate the expression of fibrosis cytokines [6]. Previous studies have demonstrated the critical role of the TGF-*β*1/Smad2/3 signaling pathway in the pathogenesis of PF [7].

MicroRNA (miRNA) is a small endogenous non-coding RNA molecule consisting of approximately 21–25 nucleotides [8] and by binding the mRNA complementary sequence of the 3′-untranslated region (3′-UTR), inhibit gene expression at the post-transcriptional level and induce inhibit protein synthesis through mRNA degradation or inhibition of translation [9]. MiRNAs Participate in various biological functions including not limited to cell division, differentiation, metabolism, apoptosis, etc [10]. Increasing evidence suggests that miRNAs play important roles in the progression of PF [11]. Among fibrosis-associated miRNAs, miRNA-132 has been found as an antifibrotic regulator in multiple organ fibrosis, including renal fibrosis, myocardial fibrosis, and liver fibrosis [12–14]. However, whether miR-132 has a role in alleviating PF remains unclear. Therefore, the aim of this study is to investigate the expression and effect of miR-132 on PF by targeting TGF-*β*1/Smad2/3 signaling pathway.

## Materials and methods

### Animal model

A total of 18 male SD rats weighing between 210 and 250 g were obtained from Chongqing Tengxin Biotechnology Co., LTD. All rat feeding and experiments were performed in accordance with the NIH Guide for the Care and Use of Laboratory Animals. The experiment was conducted in accordance with the requirements of Animal Ethics Committee of Guizhou Provincial People’s Hospital (approval number [2023] 017). Six-week-old rats were fed adaptively for one week under a 1:1 day-night cycle with free access to food and water. After 14-day intervention, the rats were anesthetized using intraperitoneal Pentobarbital sodium (100–200 mg/kg), and were euthanized by aortic exsanguinations. The visceral peritoneal tissues were collected and stored at -80°C for subsequent analyses. 4% paraformaldehyde was utilized to fix the collected tissues for pathological staining and immunohistochemistry.

### Cell lines and cell culture

The immortalized human pleural mesothelial cell lineMet-5A used in this study were purchased from Beijing Fenghui Biotechnology Co., Ltd. The Medium M199 (Gibco, Grand Island, NY) with 10% fetal bovine serum(Gibco) and 100 U/ml penicillin (Gibco) were used to culture Met-5A cells in an incubator with 100% humidity and 5% carbon dioxide at 37°C. Met-5A cells were cultured in the incubator with 100% humidity and 5% carbon dioxide at 37°C for 24h before changing the medium. TGF-*β*1 (Gibco) dissolved in phosphate-buffered saline (PBS, Gibco) with a concentration of 10 ng/ml was used to treat the Met-5A cells for 24h for further analysis, and PBS solution was used as the negative control.

### Cell transfection and treatment

The has-miR-132-3p mimics and has-miR-132-3p inhibitor were designed and purchased from the SuZhou GiMa Gene Biotech Co., Ltd (Suzhou, China). 10nM siRNA was added into the cell culture, respectively, and the infected Met-5A cells were then cultured in the incubator with 100% humidity and 5% carbon dioxide at 37°C for 24 h before changing the medium. TGF-*β*1 (Gibco) dissolved in phosphate-buffered saline (PBS, Gibco) with a concentration of 10 ng/ml was used to treat the Met-5A cells for 24 h for further analysis, and PBS solution was used as the negative control.

### Human peritoneal solution collection

The study scope was to select adults over the age of 18 who had sufficient cognitive and verbal abilities to discuss the purpose of the study and complete the survey. Samples were obtained from May 19, 2019 to July 9, 2023. We recruited a total of 26 patients who were interested in participating, all patients provided written informed consent, and then conducted a survey. After the investigation was completed, they were evaluated clinically. Authors had access to information that could identify individual participants during and after data collection. In a sterile environment, 30 ml of ascites was extracted from the dialysis hole of the dialysis patient. fitted in a 50ml sterilized,sterile enzyme-free centrifuge tube. The supernatant was removed by centrifugation at 4000 g/min and used for further qPCR experiments. The experiment was conducted in accordance with the requirements of Ethics Committee of Guizhou Provincial People’s Hospital (approval number [2019] 29).

### Quantitative real-time polymerase chain reaction (RTq-PCR)

The total RNA of Met-5A cells, rat peritoneal tissues, and human peritoneal fluids were extracted respectively using a TRIzol Kit (ThermoFisher) under the instruction. Then, a SuperScript IV Kit (ThermoFisher) was used to reversely transcribe the mRNA of each sample to cDNA for further experiment. RNA levels of miR-132-3p, TGF-*β*1, α-SMA, Smad2, and Smad3 were examined by q-PCR mix, and a S1000 PCR Thermal cycler was used to detect the system. *β*-actin served as the loading control. The mRNA expression level was calculated using the comparative period threshold (Ct) method of normalised *β*-actin. The specific primer sequences for q-PCR are shown in Table 1.

**Table 1:** Sequences of the primers for q-PCR.

Gene	Forward primer	Reverse primer
MiR-132-3p primer(rat)	ACA CTC CAG CTG GGT AAC AGT CTA CAG	CTC AAC TGG TGT CGT GGA
MiR-132-3p primer(human)	CGT AAC AGT CCA GCC ATG	TGG TGT CGT GGA GTC G
α-SMA primer (human)	GCG TGC GGC TCT ACT ACA TC	GCA CAT TCG GGT CAA CTG GTA
β-ACTIN primer (human)	TGA TGA TAT CGC CGC GCT C	CCA TCA CGC CCT GGT GC
TGF-β1 primer(human)	GCC GAC TAC GCC AAG GAG	TGT GTG TAC TCT GCT TGA ACT TGT C
Smad2 primer(human)	AGC AGA ATA CCG AAG GCA GAC G	GCT TGA GCA ACG CAC TGA AGG
Smad3 primer(human)	GTA GTA GGA GAT GGA GCA CCA GAA G	AAC CAG TGA CCA GAT GAA CC
U6	CTC GCT TCG GCA GCA CA	AAC GCT TCA GAA TTT GCG T

### Histopathology

Parietal peritoneum tissues were prepared into 4 mm thick paraffin sections, HE and Masson staining were performed using an HE staining kit and a Masson three color dyeing kit (Beijing Solarbio Science & Technology Co. Ltd., Beijing, China), according to the manufacturer’s instructions.

### Immunohistochemistry

Paraffin-embedded peritoneal tissues were dewaxed, rehydrated by sectioning, and blocked by peroxidase for 30 min. The tissue slices were high-pressure repaired by citrate repair solution (pH = 6.0) for 2 min. The tissue slices were sequentially incubated with primary antibodies of interest (TGF-*β*1[1:100], α-SMA [1:100], Smad2 [1:50], Smad3 [1:50]) overnight at 4°C. The secondary antibody (horseradish peroxidase [HRP] labelled) was incubated at 37°C for 30 min. Finally, the signal was visualized by using a 3, 3’-diaminobenzidine (DAB) kit (Beijing Zhongshan Jinqiao Biotechnology, Beijing, China). Then counterstained with hematoxylin, dehydrated with gradient alcohol and xylene and sealed with neutral balsam.

The primary antibodies used were as follows: Smad2 (A00090-1, Boster, 1:50 dilution; China); Smad3 (BA4559, Boster, 1:50 dilution; China); α-SMA (BM0002, Boster, 1:100 dilution; China); TGF-*β*1 (BA0290, Boster, 1:100 dilution; China).

### Immunofluorescence

The cells were grafted to 2.5 × 10^4/well seed containing slides in 24-well plates and cultured for 48 h. After collecting the cells, they were fixed with 4% paraformaldehyde. Cells were permeabilized with 0.2% tritonX-100 for 1h and then blocked with 5% BSA and sequentially incubated with the primary antibodies of interest and the next day after incubation using phasic secondary antibodies, The samples were washed with saline-sodium citrate (SSC) twice, stained with DAPI, and the slides were removed and sealed with an anti-fluorescence quenched tablet, and tested by fluorescence test under laser scanning confocal microscope.

The primary antibodies used were as follows: Smad2 (A00090-1, Boster, 1:200 dilution; China); Smad3 (BM3919, Boster, 1:200 dilution; China); α-SMA (BM0002, Boster, 1:200 dilution; China); TGF-*β*1 (MA00019, Boster, 1:200 dilution; China).

### Western blot

Cells were harvested and lysed in whole protein extraction reagent (Solarbio, Beijing, China) containing protease inhibitors and tyrosine and serine-threonine phospho-inhibitors for 30min. The supernatant was resolved at 12000rpm for 30 min after 95°C degeneration for 15min, and the proteins were separated on SDA-PAGE (Solarbio, Beijing, China) and transferred to polyvinylidene fluoride (PVDF) membranes. These were then incubated in Fast sealing liquid for 10 min and incubated with TGF-*β*1 primary antibody (BA0290, Boster, 1:2000 dilution; China), and β-actin (ab8227, immunoway 1:5000 dilution; China). After incubation with the corresponding HRP coupled secondary antibodies (Boster, Wuhan, China), the protein were treated at 37°C for 2 h. Protein sample reagents (Millipore, Billerica, MA) were detected by enhanced chemiluminescence (ECL), and signals were quantified by Image J.

### Statistical analysis

All experiments were repeated three times. SPSS 26.0 (IBM, Armonk, NY) statistical software was used for statistical analysis. The results were expressed with mean ± standard deviation for normally distributed variables, and median (interquartile range) for non-normally distributed variables. The differences among groups were compared using one-way analysis of variance or the Kruskal-Wallis test. A value of *P*< 0.05 was considered statistically significant.

## Results

### The expression of miR-132-3p decreased in the peritoneal tissues of PF rats

**Fig 1:**
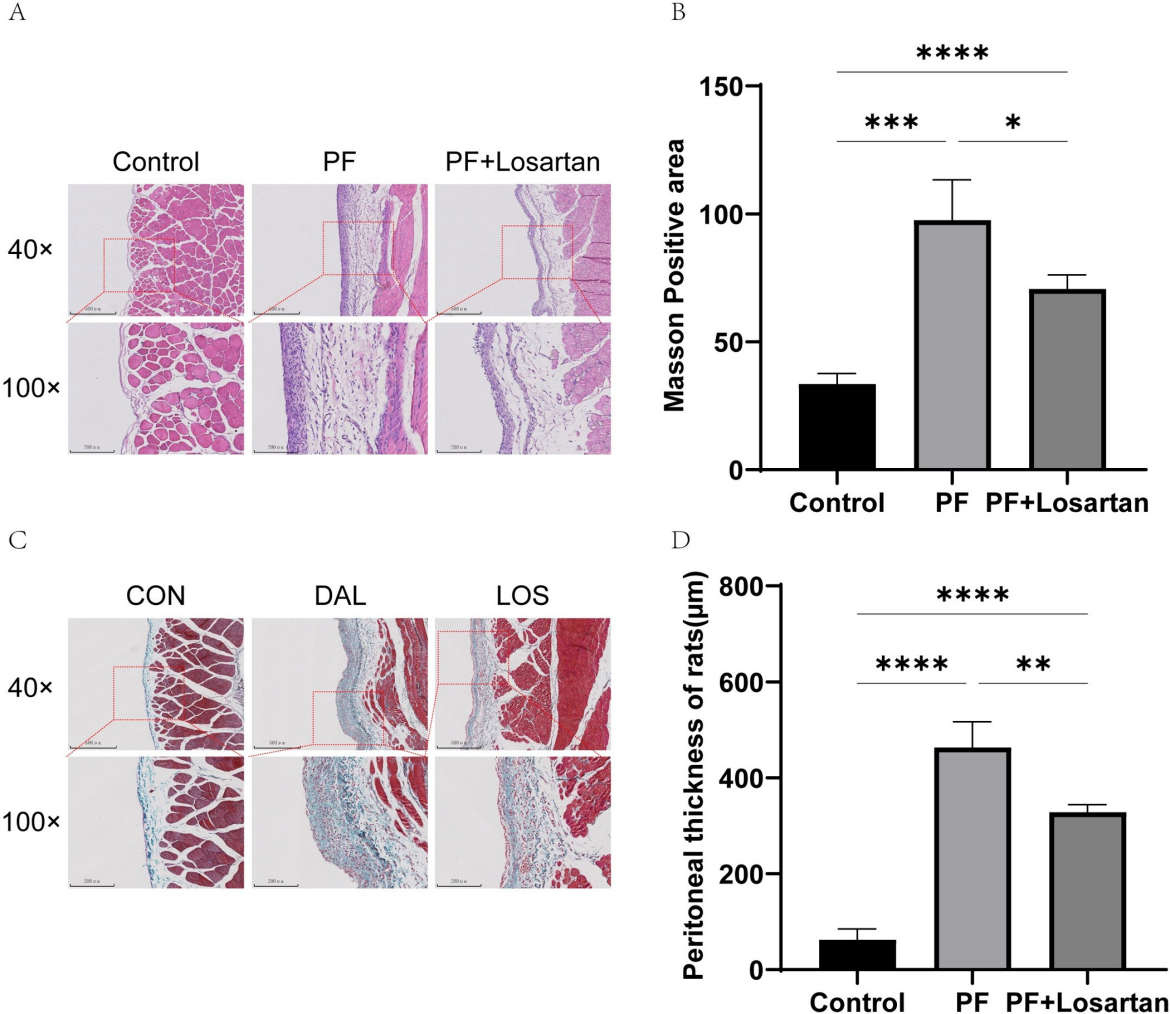
Changes of peritoneal structure and function in rats. (A) HE staining results;(B) HE staining positive area statistics; (C) Masson staining results;(D) Masson staining positive area statistics. All data are presented as mean ± SD. n = 6 for each group. *p < 0.05, **p < 0.01, ***p < 0.001and ****p < 0.0001 between the indicated groups.

To confirm the morphological changes of PF, rat peritoneal tissues were collected and stained pathologically. HE staining results showed that in the control group, single-layer mesothelial cells were continuously distributed with complete structure and loose inter-subcutaneous connective tissue without hyperplasia or exudation. In the PF group, there was a loss and discontinuous distribution of peritoneal mesothelial cells, collagen exposure, hyperplasia and thickening of subcutaneous stroma, tissue edema with exudation of cellulose and inflammatory cells, and increased vascular structure in stroma (Fig 1A and 1C). Masson staining results showed that the thickness of subcutaneous connective tissue increased significantly in the PF group, compared with the control group, which confirmed that the PF model was established successfully (Fig 1B and 1D).

**Fig 2:**
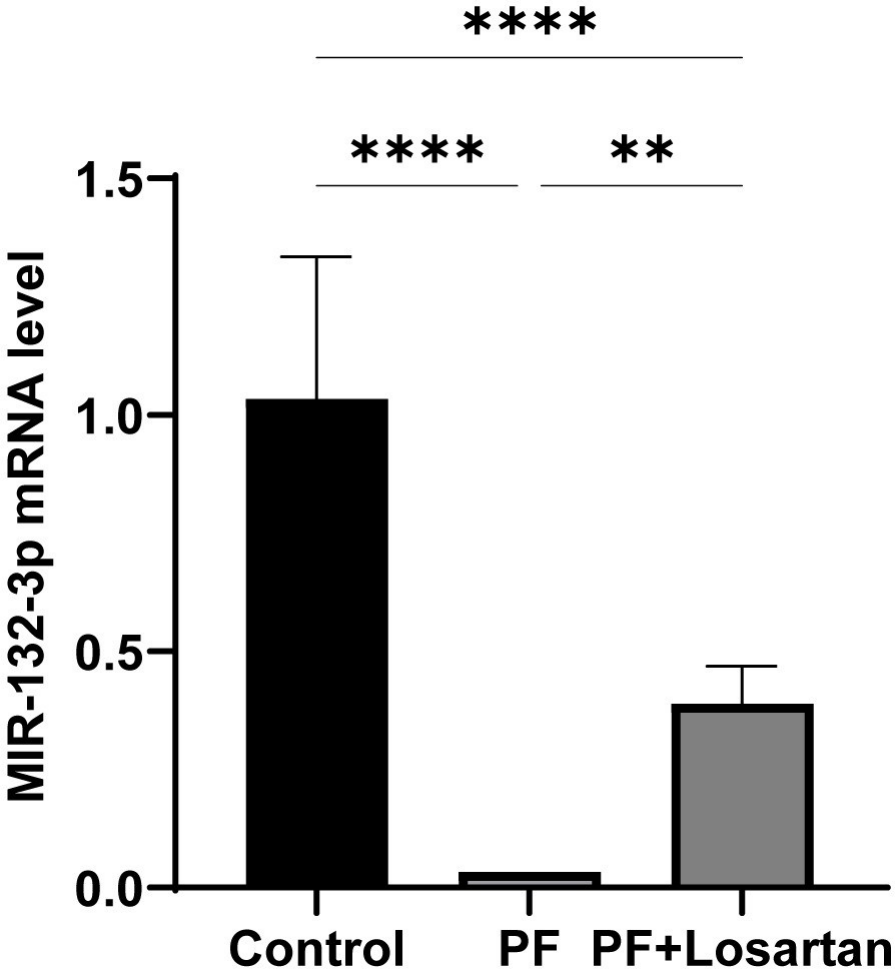
Changes of miR-132-3p in rat peritoneum. All data are presented as mean ± SD. n = 6 for each group. **p < 0.01; ****p < 0.0001 between the indicated groups.

In addition to the morphological changes of PF, we found that the expression of miR-132-3p was down-regulated (Fig 2), so we speculated that miR-132-3p played a role in the process of PF.

### MiR-132-3p was associated with the activity of TGF-*β*1/Smad2/3 signaling pathway in PF rats

**Fig 3:**
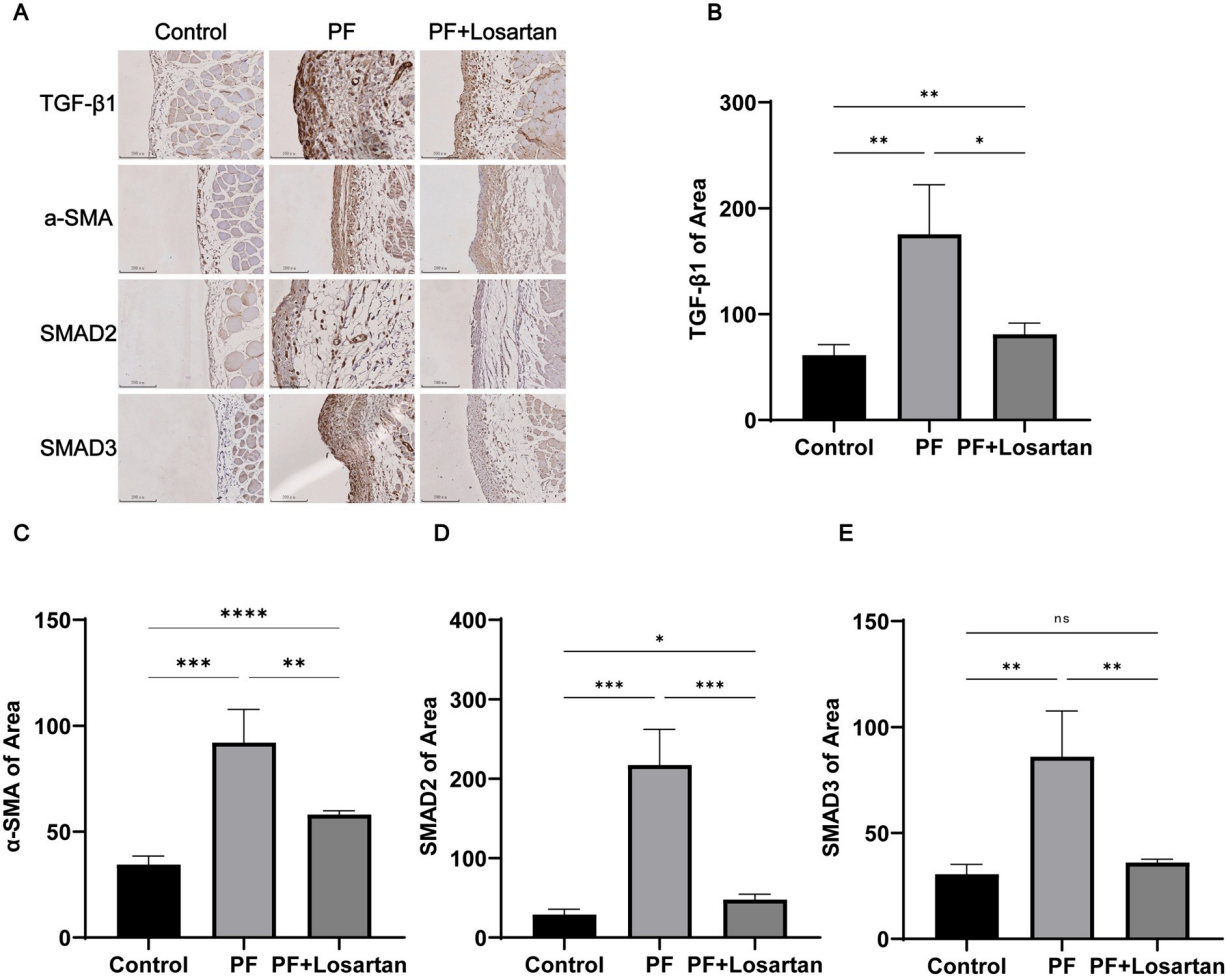
Changes of fibrotic proteins in rat peritoneum. (A) Area semi-quantitative statistical analysis by immunohistochemistry; (B-E)Expression of TGF-*β*1, smad2, smad3,col1a1 col3a1and α-SMA by immunohistochemistry. All data are presented as mean ± SD. n = 6 for each group. *p < 0.05; **p < 0.01; ***p < 0.001; between the indicated groups.

To further explore the effect of miR-132-3p on PF in SD rats, the expressions of fibrosis-related molecules, including TGF-*β*1, Smad2, Smad3, and α-SMA, were detected by immunohistochemistry. Semiquantitative analysis of the positive expression areas showed that the protein levels of TGF-*β*1, α-SMA, Smad2, Smad3 in the PF group significantly increased, compared with the control group (Fig 3A–3E). These results suggest that miR-132-3p were associated with TGF-*β*1/Smad2/3 signaling pathway and might be a potential therapeutic target for PF.

### Losartan treatment improved PF in rats and induced the upregulation of miR-132-3p expression

Compared with the PF group, the thickness of the peritoneum was significantly reduced and the degree of fibrosis was alleviated, which indicated that losartan had a certain inhibitory effect on the process of PF (Fig 1A–1D). In addition, the expression of fibrosis-related factors was suppressed by losartan treatment, as indicated by the expression of proteins involved in TGF-*β*1/Smad2/3 signaling pathway (Fig 3A–3E). We further detected the expression of miR-132-3p in the peritoneal tissues of losartan-treated rats, and found that the expression of miR-132-3p in the losartan-treated group was significantly higher than that in the PF group (Fig 2). This result confirmed that miR-132-3p was involved in the process of PF and down-regulated the TGF-*β*1/Smad2/3 signaling pathway. Therefore, we hypothesized that miR-132-3p could improve PF by regulating TGF-*β*1/Smad2/3 signaling pathway, and we performed the following cell experiments to verify this hypothesis.

### MiR-132-3p was downregulated in TGF‐*β*1 stimulated Met‐5A cells

**Fig 4:**
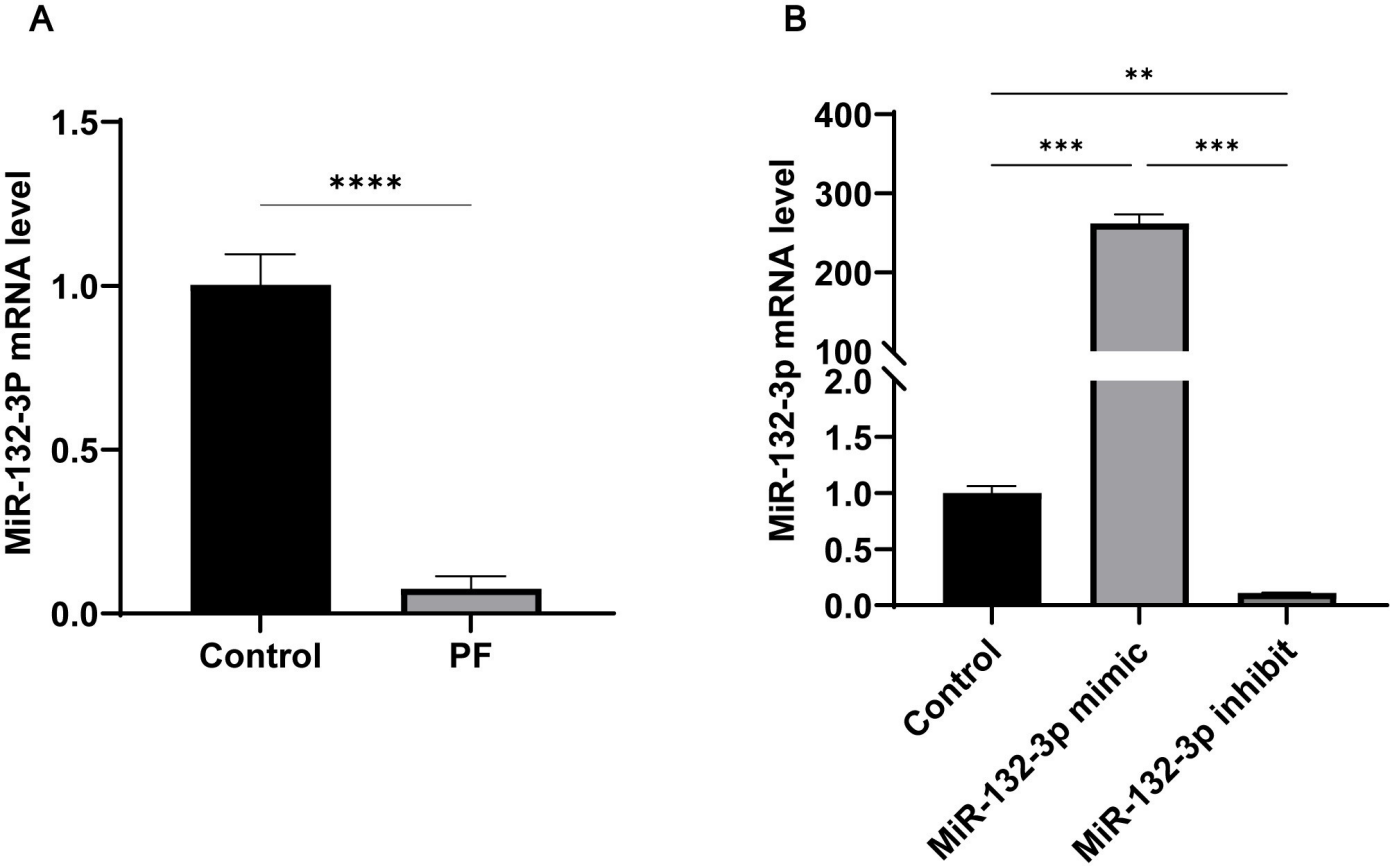
MiR-132-3p was downregulated in TGF-*β*1 stimulated Met-5A cells and miR-132-3p mimics and miR-132-3p inhibitor transfected Met-5A cells. MiR-132-3p mRNA expression assessed by qRT‐PCR in Met‐5A cells exposed to control conditions (unstimulated) or TGF‐*β*1 for 24 h; (B) Met‐5A cells were transfected with, miR-132-3p mimics or miR-132-3p inhibitor for 24 h. Non-transfected cells were included as control. MiR-132-3p inhibitor mRNA expression assessed by qRT‐PCR. All data are presented as mean ± SD. n = 3 for each group. **p < 0.01; ***p < 0.001 and ****p < 0.0001 between the indicated groups.

We found that TGF-*β*1 treatment of human Met-5A cells induced the down-regulation of miR-132-3p expression (Fig 4A). We confirmed that miR-132-3p was negatively related to PF, which was similar to the animal experiments.

### Overexpression and inhibition of miR-132-3p in Met-5A cells

To regulate the expression level of miR-132-3p in Met-5A cells, we transfected has-miR-132-3p mimics and has-miR-132-3p inhibitor, respectively. Has-miR-132-3p transfection inhibitor decreased miR-132-3p in Met-5A cells, while has-miR-132-3p mimics had the opposite result (Fig 4B).

### Overexpression of miR-132-3p down-regulated TGF-*β*1/Smad2/3 signaling pathway in Met-5A cells

**Fig 5:**
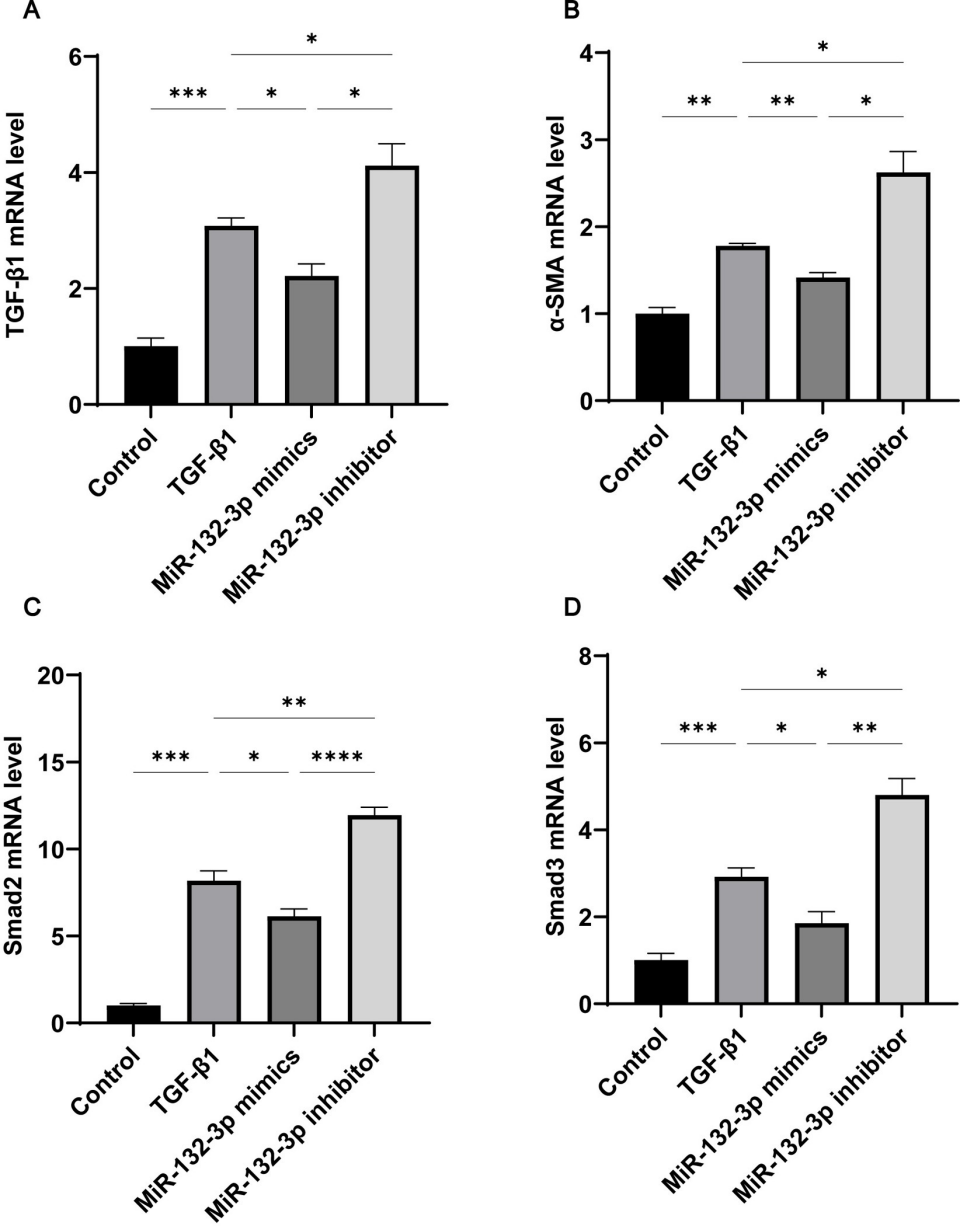
Overexpression of miR-132-3p inhibits the TGF-*β*1/Smad2/3 signaling pathway in TGF-*β*1 induced MET-5A cells. Quantitative real time-PCR was used to measure the mRNA levels of(A)TGF-*β1*;(B)α-SMA;(C)Smad2;(D)Smad3. All data are presented as mean ± SD. n = 4 for each group. *p < 0.05; **p < 0.01; ***p < 0.001 and ****p < 0.0001 between the indicated groups.

**Fig 6:**
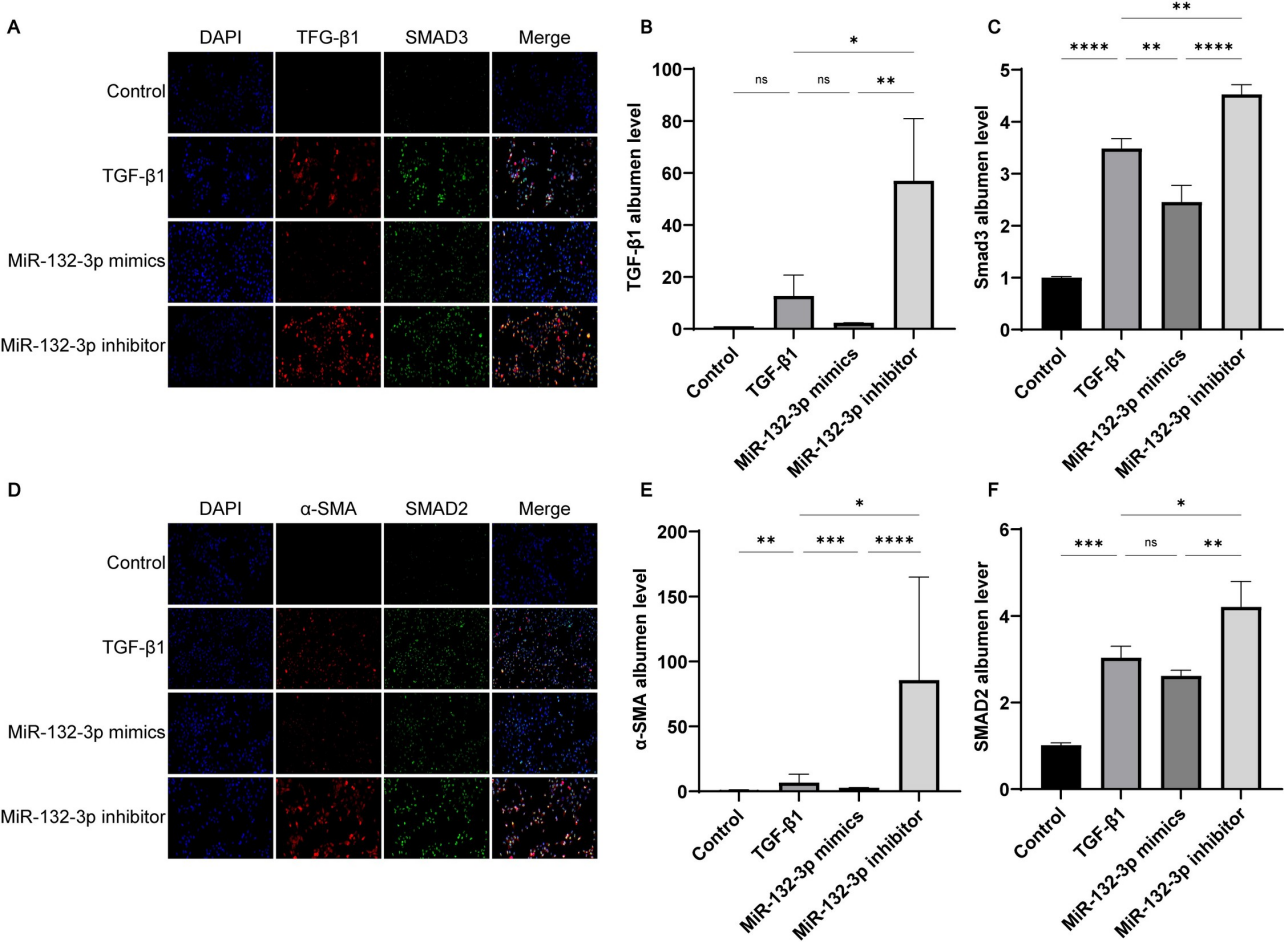
The effect of MiR-132-3p on protein expression in TGF-*β*1/Smad2/3 signaling pathway was observed by immunofluorescence. (A and D) Immunofluorescence was used to measure the protein expression levels of(B)TGF-*β*1;(C)Smad3;(E)*α*-SMA;(F)Smad2. All data are presented as mean ± SD. n = 3 for each group. *p < 0.05; **p < 0.01; ***p < 0.001 and ****p < 0.0001 between the indicated groups.

**Fig 7:**
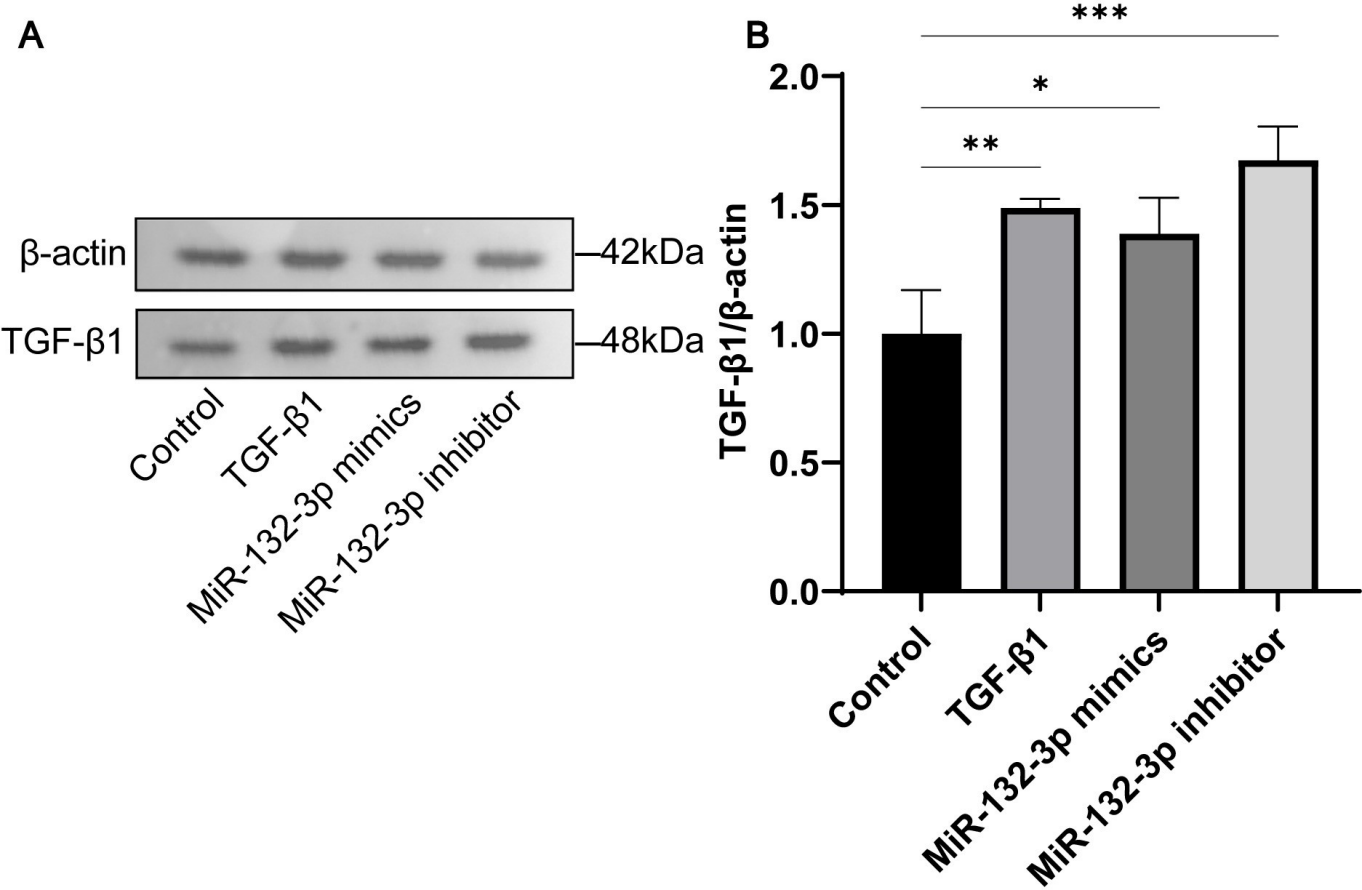
Regulation of TGF-*β*1 protein expression by MiR-132-3p was verified using WB. (A)The protein levels of fibrosis molecules TGF-*β*1. (B)The semi-quantitative analysis of imprinting of related proteins. All data are presented as mean ± SD. n = 3 for each group. *p < 0.05; **p < 0.01 and ***p < 0.001 between the indicated groups.

To demonstrate the regulatory effect of miR-132-3p on TGF-*β*1-induced PF, has-miR-132-3p mimics and has-miR-132-3p inhibitor were transfected into Met-5A cells and then treated with TGF-*β*1. As shown in Fig 5A–5D, the mRNA expression of TGF-*β*1, Smad2, Smad3, and *α*-SMA showed similar trends in response to different stimuli. Similarly, these proteins were also found to have a similar trend of expression by Fluorescence in situ hybridisation(Fig 6A–6F). TGF-*β*1 stimulation significantly upregulated the expression of all these proteins, whereas in vitro overexpression of has-miR-132-3p significantly inhibited their expression. We similarly verified the expression of the TGF-*β*1 protein in the Western blot experiments (Fig 7A and 7B). Taken together, the overexpression of has-miR-132-3p inhibited TGF-*β*1-induced fibrosis progression in Met-5A cells.

### The expression of miR-132-3p decreased in a time-dependent mode in PD patients

**Fig 8:**
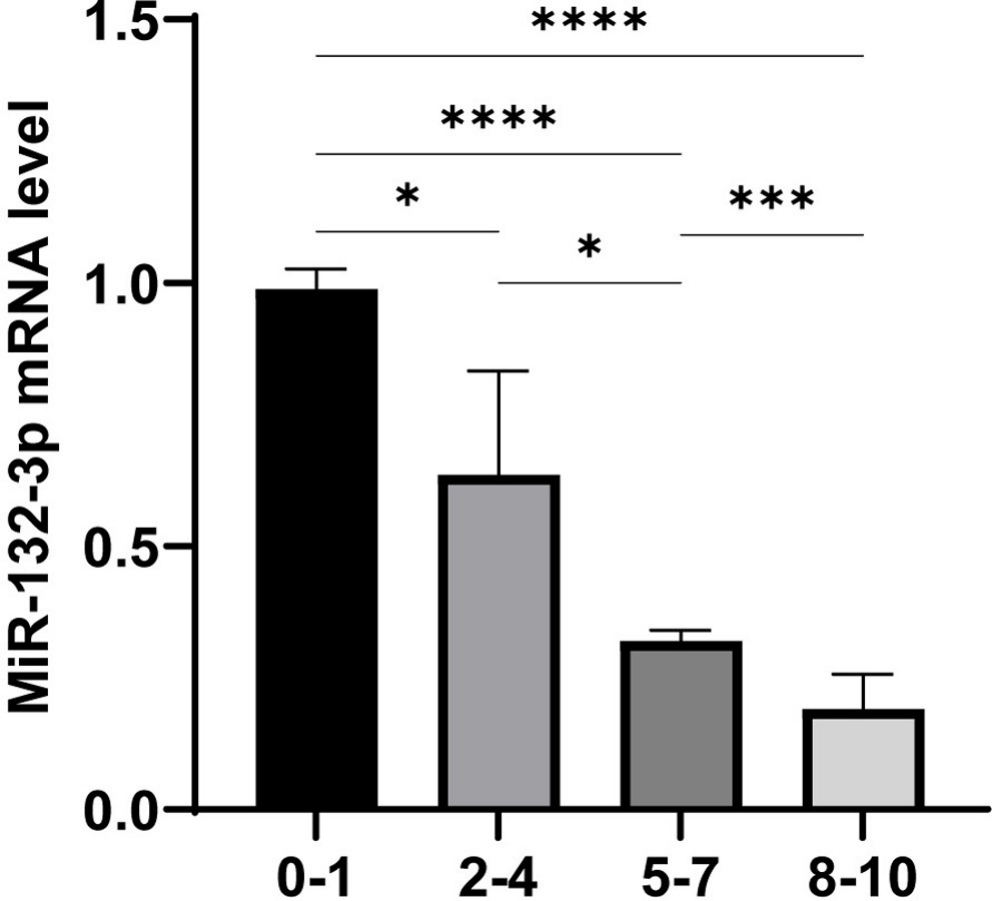
Changes of miR-132-3p in peritoneal dialysis patients with different duration. All data are presented as mean ± SD. n = 5 for <1 year group, n = 5 for 1-5year group, n = 7 for 5-8year group,n = 9 for >8year group. *p < 0.05; **p < 0.01 and ***p < 0.001 between the indicated group.

To verify whether the expression of miR-132-3p changes during PD duration, we collected the peritoneal solution from 26 PD patients (18–80 years) with different PD duration and divided them into four groups: <1 year, 1–5 years, 5–8 years, and >8 years. Compared with patients in <1 year as the control group, we found that the inhibition effect of miR-132-3p expression enhanced in three other groups with longer PD duration (Fig 8). The results also showed that miR-132-3p in peritoneal solution decreased gradually with the increase of PD duration in PF patients, which indicated that miR-132-3p expression was inversely associated to the degree of PF. We may utilize miR-132-3p as a treatment target, and further explore the potential ability of inhibiting or delaying the process of PF.

## Discussion

In this study, we demonstrated that miR-132 expression is down-regulated in peritoneal mesothelial cells where the fibrotic response accumulated. These findings were confirmed by qPCR *in vitro*, *in vivo*, even in PD patients. Therefore, our data supported an ameliorative role of miR-132 in PF.

Previous studies have shown that miR-132 plays an antifibrotic role in multiple organ fibrosis, which was consistent with this study. Many previous studies have shown that miR-132 can reduce the process of fibrosis. Guoyu Wang *et al*. [12] demonstrated that miR-132 can reduce myocardial fibrosis, and Roel Bijkerk *et al*. [13] demonstrated that miR-132 can reduce renal fibrosis. In this study, we found that the expression of miR-132 was down-regulated in PF, whether in PF rats, cell models, or human peritoneal solution.

Previous researches have studied the effect of losartan and similar drugs on the treatment of PF. In this study, we demonstrated the significant therapeutic effect of losartan on PF in SD rats,which was similar with previous studies. Junju Zou *et al*. [15] confirmed that losartan ameliorated renal interstitial fibrosis, and Wilson SE *et al*. [16] also found that losartan can prevent and treat corneal scarring fibrosis. Guo F *et al*. [17] verified that losartan could attenuate pulmonary fibrosis in rat. We also found that miR-132 expression was significantly up-regulated in peritoneal mesothelial cells after treatment.

TGF-*β*1 plays an important role in the progression of PF, and TGF-*β*1/smad2/3 signaling pathway plays a role in PF through cell signal transduction. Accumulating evidence suggest that divergent miRNAs participates in the liver fibrotic process, which partially regulates members of the TGF-*β*/SMAD signaling pathway, which address the possibility of novel therapeutic approaches to hepatic fibrosis [18]. TGF-*β* levels are highly correlated with the activated pro-fibrotic pathways and disease progression. As the key driver of renal fibrosis, its down-stream Smad2 and Smad3 are activated in the fibrotic kidney of CKD patients and animal models. TGF-*β*1/Smad signaling regulates renal fibrosis via epigenetic-correlated mechanisms and promotes pro-fibrotic gene expression [19]. The inhibitor of TGF-*β*/Smad signal pathways may be a strategy for prophylactic treatment of pulmonary fibrosis progression [20], and its cooperation with the expression of hepatocyte growth factor in pulmonary capillaries reduced silicosis fibrosis [21].

Although the expression levels of TGF-*β*1, α-SMA, SMAD2 and SMAD3 vary greatly in animal models, it is not possible to confirm their relationships with miR-132. MiR-132-3p overexpression was confirmed to down-regulate the expression of α-SMA, smad2 and smad3 in TGF-*β*1-stimulated MET-5A cells by transfection with miR-132-3p overexpression or inhibition. This suggests that miR-132-3p plays an important role in inhibiting PF.

MiR-132 acts as a tumor suppressor inhibited the migration and invasion of cancer cells through TGF-*β*1/Smad2/3 signals significantly. This indicate that miR-132 may be a suitable therapeutic target for the treatment of oral squamous cell carcinoma [22]. Upregulation of miR-132 inhibits TGF- β signaling, thereby improving epithelial-mesenchymal transition in human prostate cancer cells [23]. The expression levels of miR-132 were increased in MI-induced heart failure, along with the expression levels of collagen I, collagen III, TGF-*β*, and α-SMA. Up-regulation of miR-132 ameliorated cardiac dysfunction, and miR-132 attenuated cardiac fibrosis in myocardial infarction-induced heart failure rats, which may be a therapeutic strategy for heart failure and cardiac fibrosis [12].

The peritoneal solution of 26 PD patients was collected. According to the duration of PD, it was found that the expression of miR-132-3p significantly decreased during the PD duration. Therefore, we hypothesized that the increase in the degree of PF was in direct proportion to the decrease in miR-132-3p, which further confirmed that miR-132-3p was negatively correlated with PF. This has not been found in previous studies, and further studies with a larger sample size is needed to explore more effective treatment directions.

In this study, we aimed to investigate the regulatory role of miR-132-3p in the PF induced by TGF-*β*1 signaling. We reported for the first time that miR-132-3p inhibited the production of extracellular matrix proteins activated by TGF-*β*1/Smad2/3 signaling pathway during the formation of PF, and we found the similar relationship between miR-132-3p and PF in vitro and in vivo, and even in humans. Our findings may provide new insights into the mechanism of mesodermal-mesenchymal transition during the progression of PF. But regard with phosphorylation, we indeed did not perform the related experiment in the phosphorylation levels, and further research need to complete it.

## Conclusions

Taken together, this study identified miR-132 as an important and novel regulator of PF. Up-regulation of miR-132 through the TGF-*β*1/Smad2/3 signaling pathway is of benefit to delay the progression of PF and the therapeutic approach aimed at upregulated miR-132 deserves attention as a potential treatment for PF.

## Supporting information

S1 File This is the raw data of IHC-1 in Rat. (ZIP)

S2 File This is the raw data of IHC-2 、qPCR、Masson and HE in Rat、qPCR in human peritoneal solution and qPCR in Met-5A cell. (ZIP)

S3 File This is the raw data of Immunofluorescence in Met-5A cell. (ZIP)

S4 File This is the raw data of Western blot in Met-5A cell. (ZIP)
