# Supplementary figures and images for: MiR-132-3p suppresses peritoneal fibrosis induced by peritoneal dialysis via targeting TGF-β1/Smad2/3 signaling pathway
Source: PLoS One. 2024 Apr 11;19(4):e0301540. doi: 10.1371/journal.pone.0301540 (PMC11008817; doi:10.1371/journal.pone.0301540)

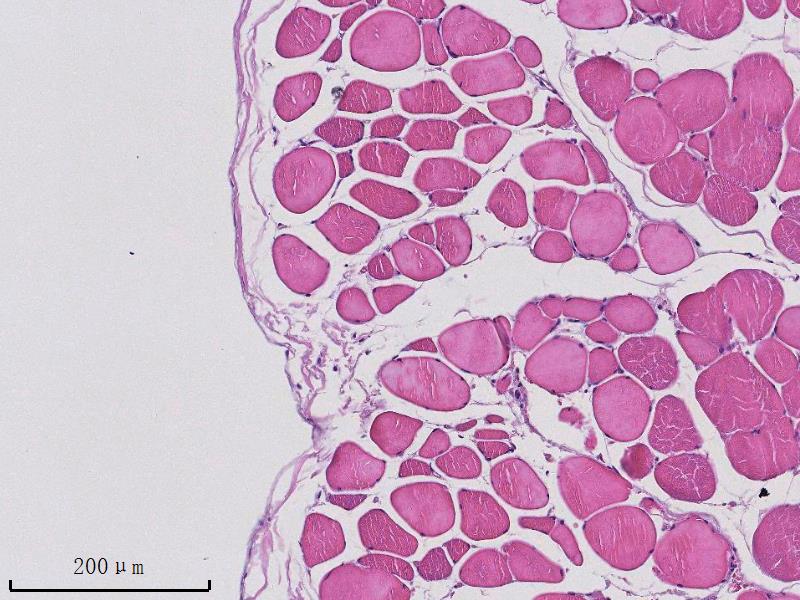

Supplement: S2 File — (ZIP) [file pone.0301540.s002.zip › raw data 2/HE/HE/23-10-1.jpg]

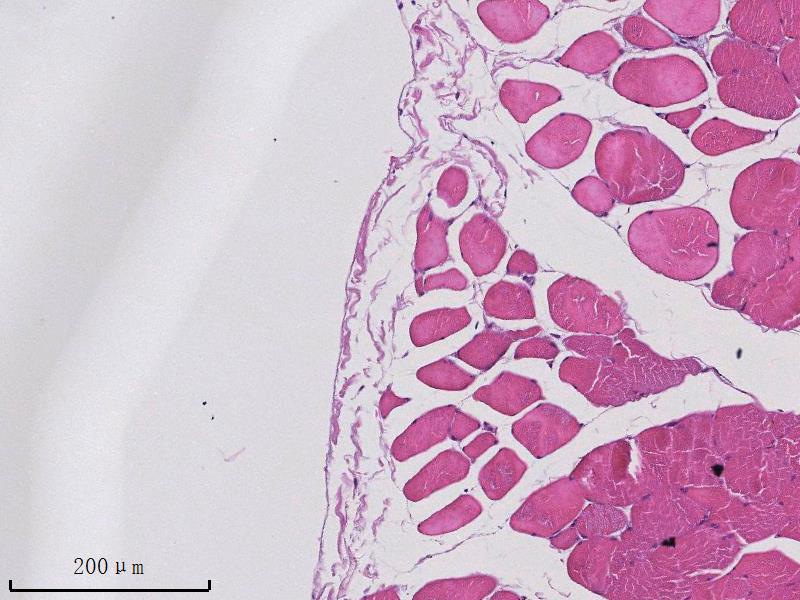

Supplement: S2 File — (ZIP) [file pone.0301540.s002.zip › raw data 2/HE/HE/23-10-2.jpg]

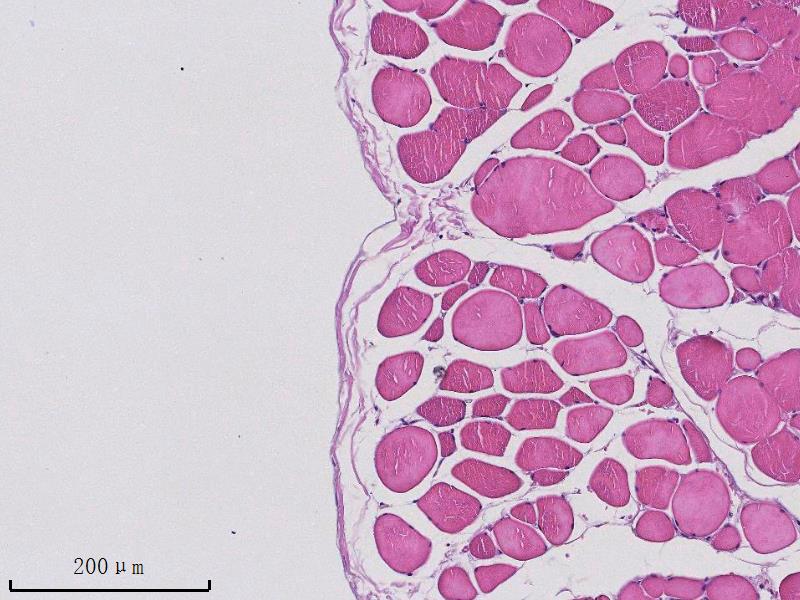

Supplement: S2 File — (ZIP) [file pone.0301540.s002.zip › raw data 2/HE/HE/23-10-3.jpg]

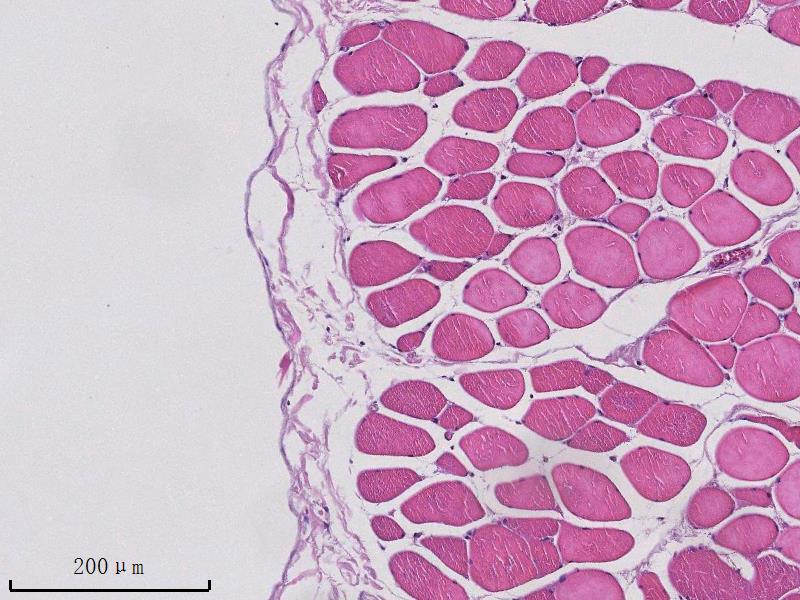

Supplement: S2 File — (ZIP) [file pone.0301540.s002.zip › raw data 2/HE/HE/23-10-4.jpg]

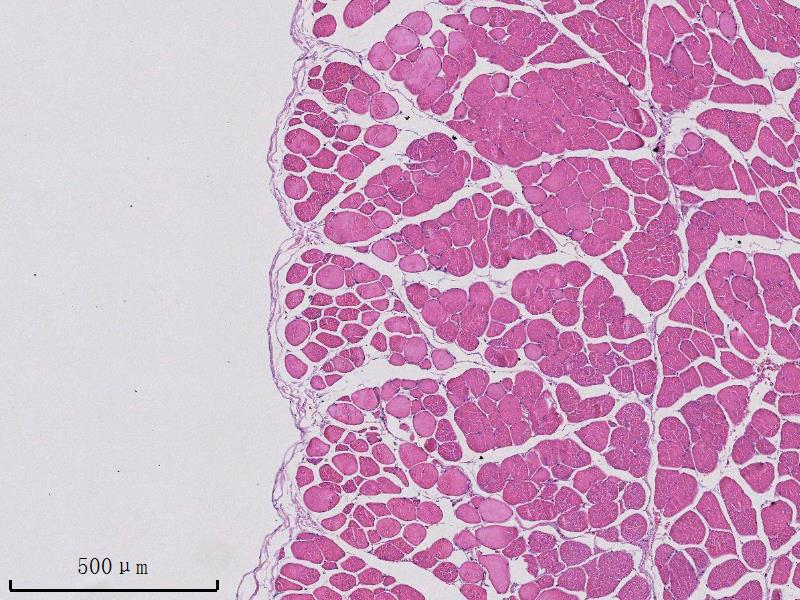

Supplement: S2 File — (ZIP) [file pone.0301540.s002.zip › raw data 2/HE/HE/23-4-1.jpg]

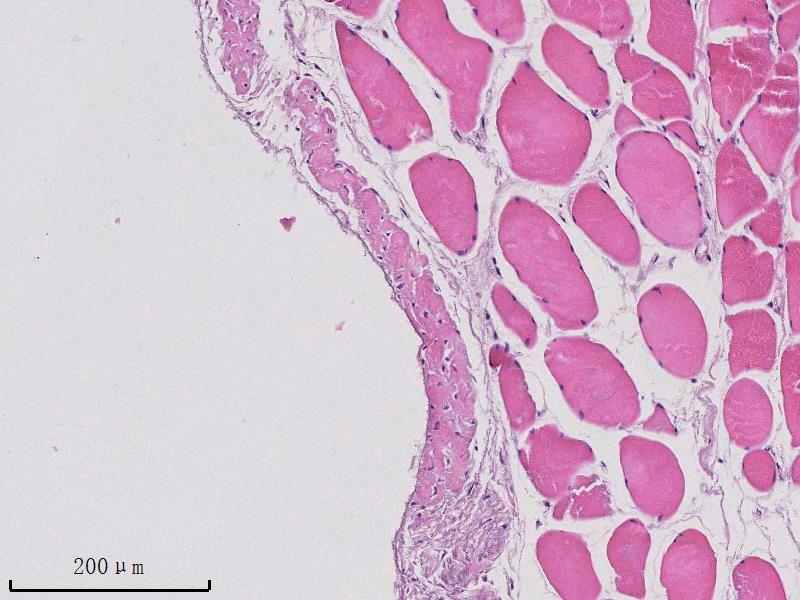

Supplement: S2 File — (ZIP) [file pone.0301540.s002.zip › raw data 2/HE/HE/24-10-1.jpg]

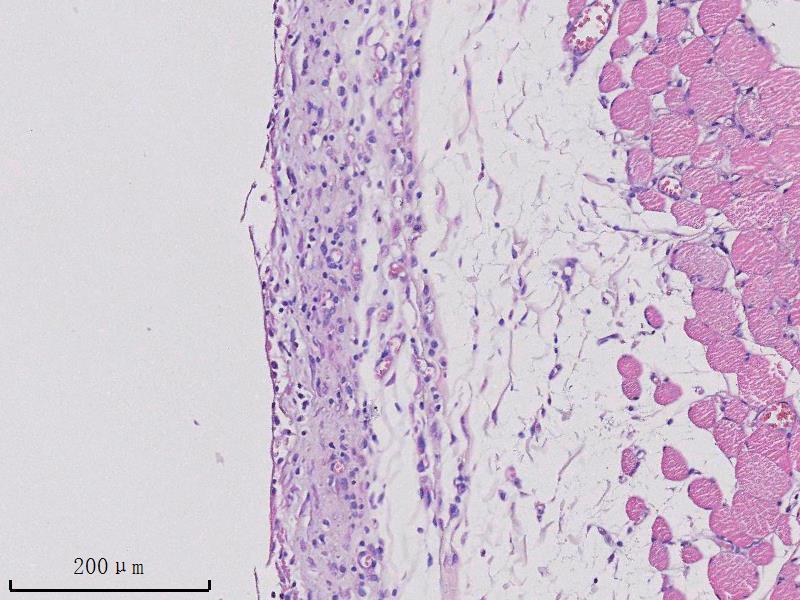

Supplement: S2 File — (ZIP) [file pone.0301540.s002.zip › raw data 2/HE/HE/32-10-1.jpg]

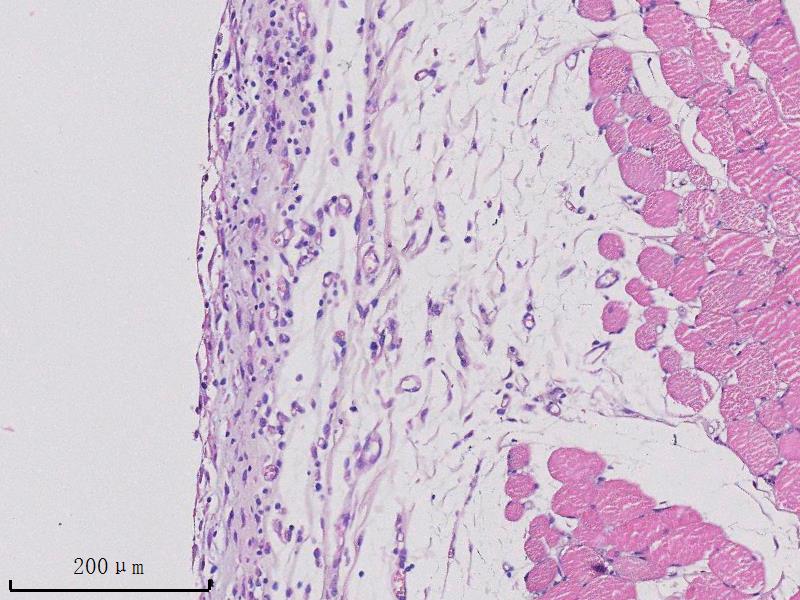

Supplement: S2 File — (ZIP) [file pone.0301540.s002.zip › raw data 2/HE/HE/32-10-2.jpg]

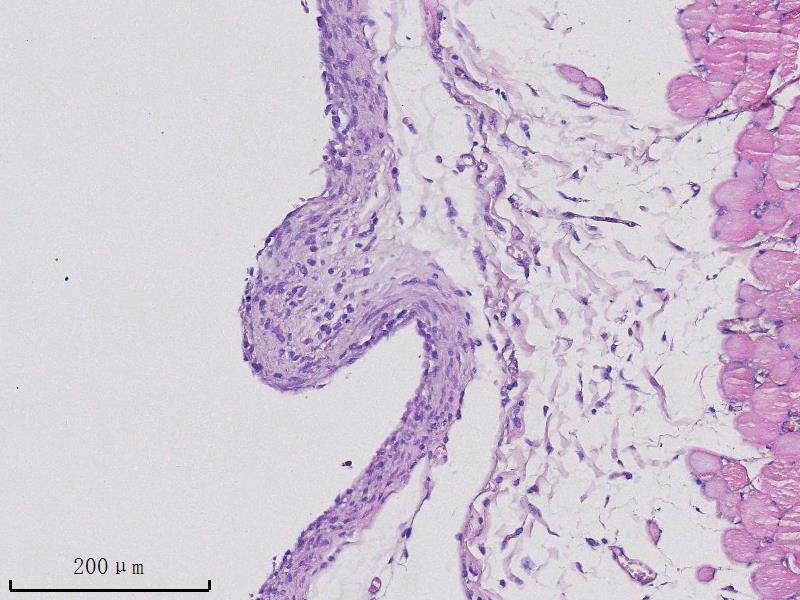

Supplement: S2 File — (ZIP) [file pone.0301540.s002.zip › raw data 2/HE/HE/34-10-1.jpg]

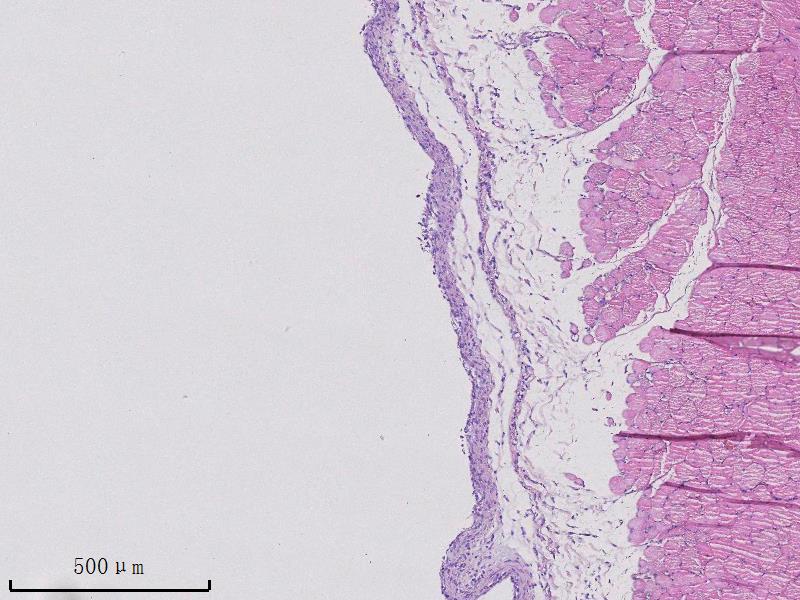

Supplement: S2 File — (ZIP) [file pone.0301540.s002.zip › raw data 2/HE/HE/34-4.jpg]

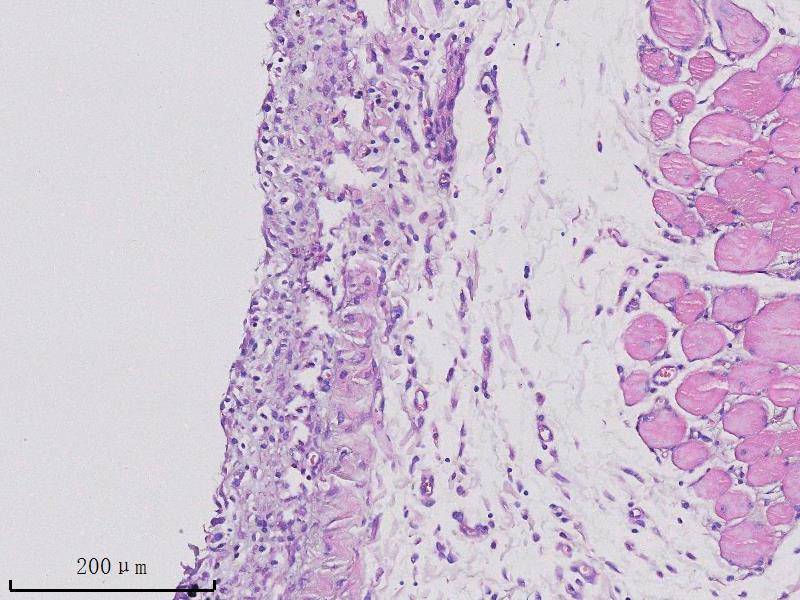

Supplement: S2 File — (ZIP) [file pone.0301540.s002.zip › raw data 2/HE/HE/35-10-1.jpg]

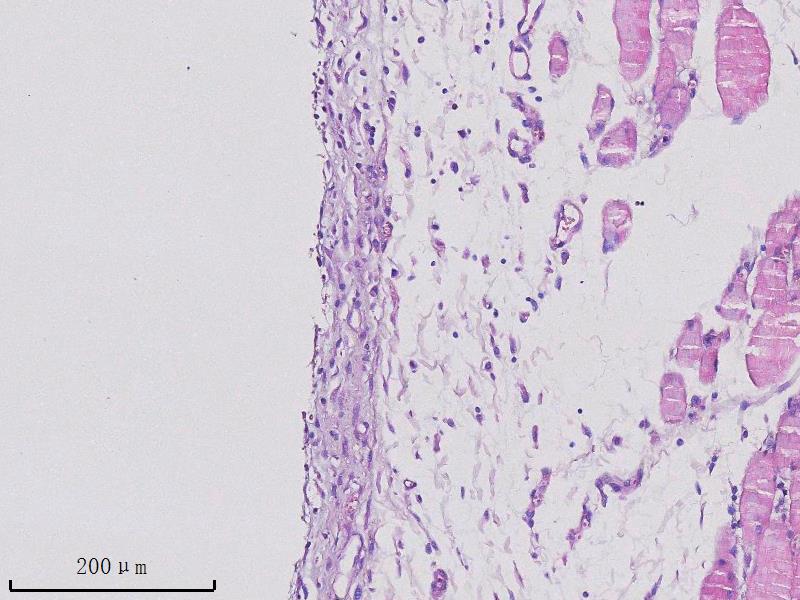

Supplement: S2 File — (ZIP) [file pone.0301540.s002.zip › raw data 2/HE/HE/35-10-2.jpg]

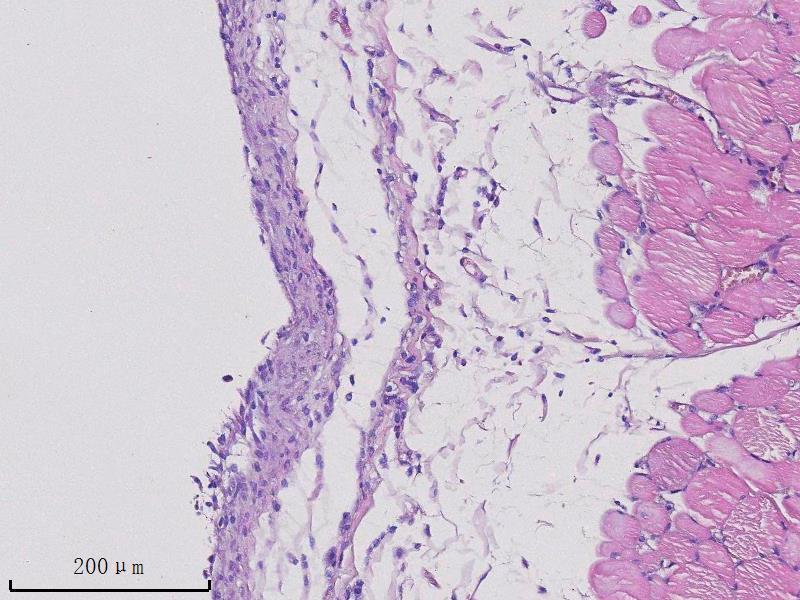

Supplement: S2 File — (ZIP) [file pone.0301540.s002.zip › raw data 2/HE/HE/35-10-3.jpg]

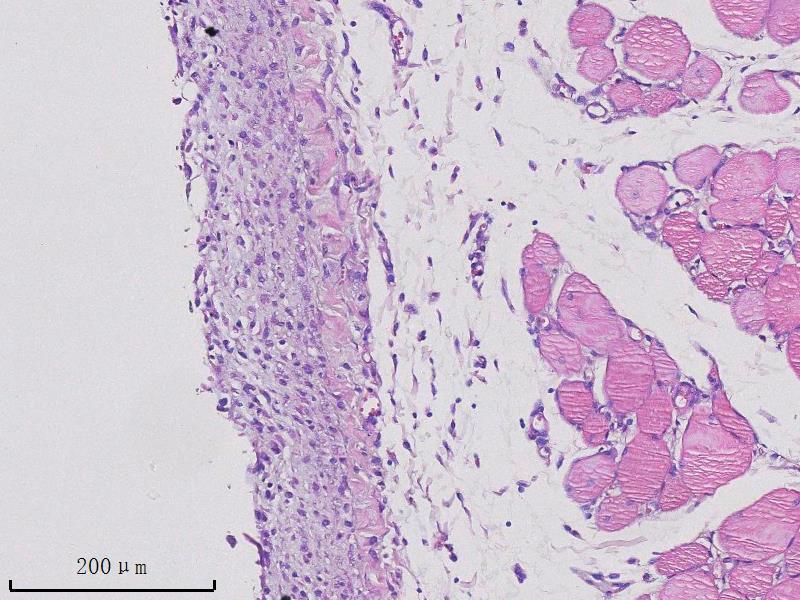

Supplement: S2 File — (ZIP) [file pone.0301540.s002.zip › raw data 2/HE/HE/35-10.jpg]

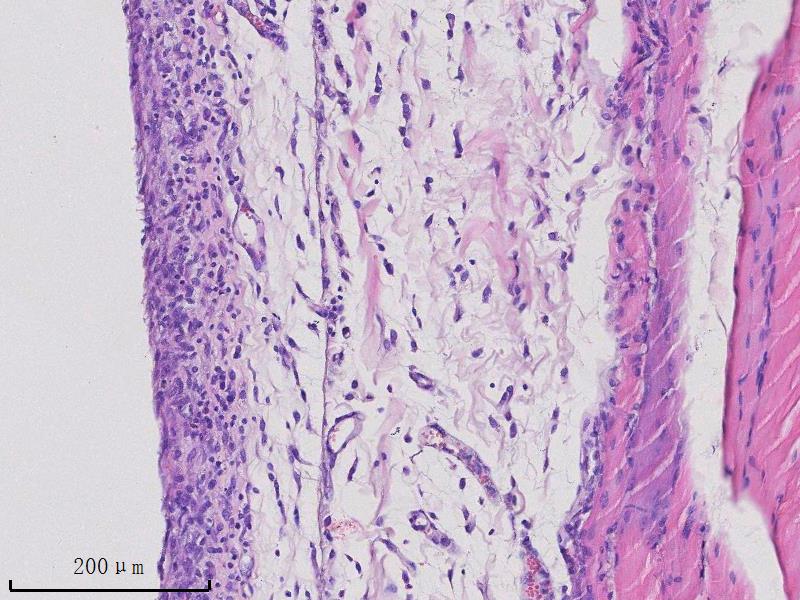

Supplement: S2 File — (ZIP) [file pone.0301540.s002.zip › raw data 2/HE/HE/44-10.jpg]

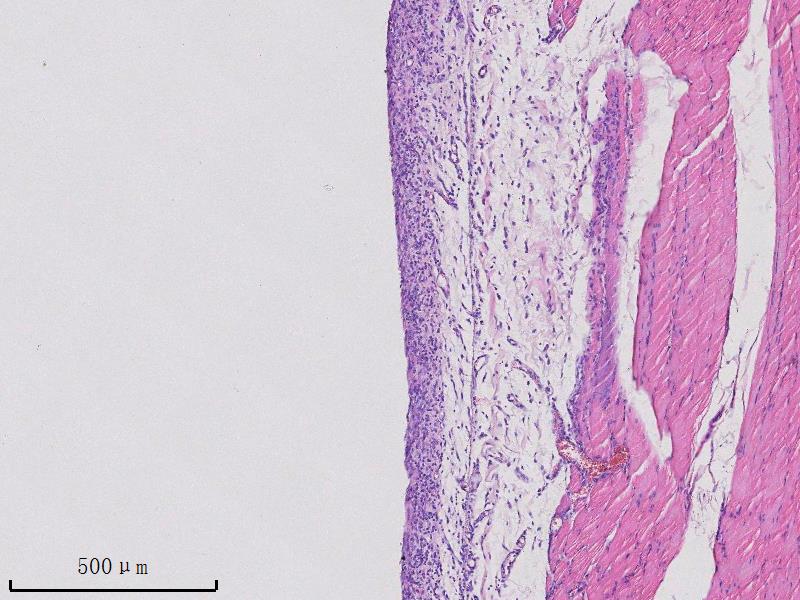

Supplement: S2 File — (ZIP) [file pone.0301540.s002.zip › raw data 2/HE/HE/44-4.jpg]

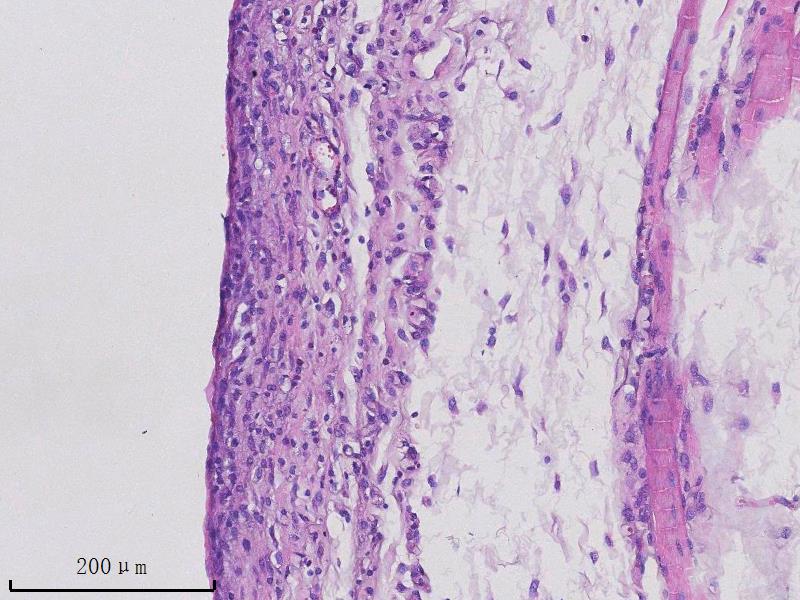

Supplement: S2 File — (ZIP) [file pone.0301540.s002.zip › raw data 2/HE/HE/51-10-1.jpg]

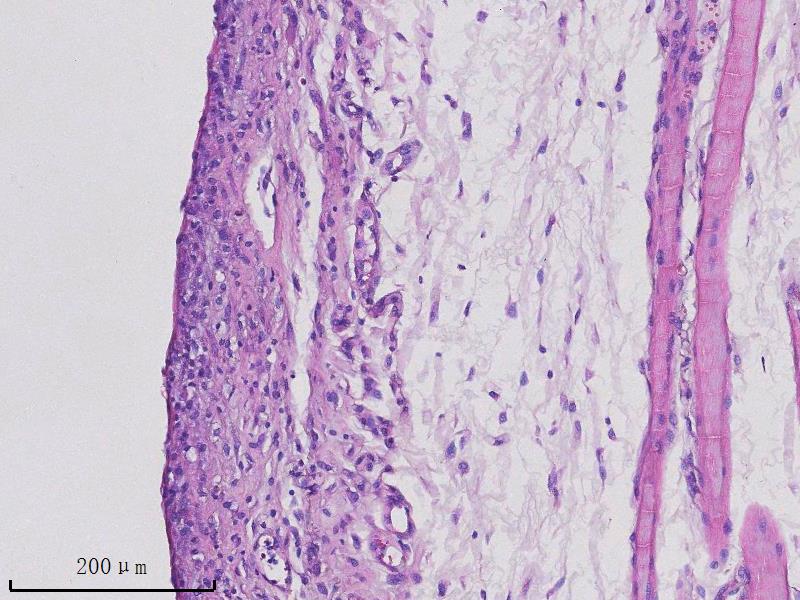

Supplement: S2 File — (ZIP) [file pone.0301540.s002.zip › raw data 2/HE/HE/51-10.jpg]

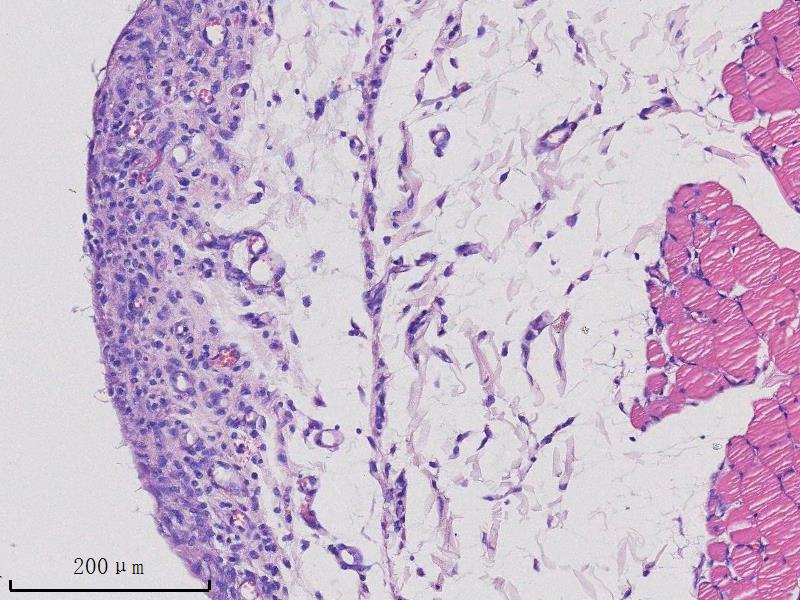

Supplement: S2 File — (ZIP) [file pone.0301540.s002.zip › raw data 2/HE/HE/54-10-1.jpg]

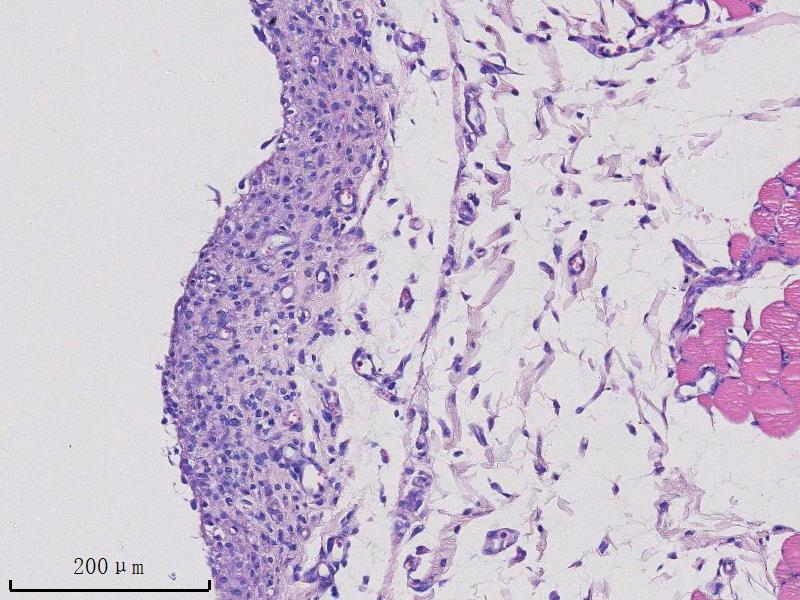

Supplement: S2 File — (ZIP) [file pone.0301540.s002.zip › raw data 2/HE/HE/54-10-2.jpg]

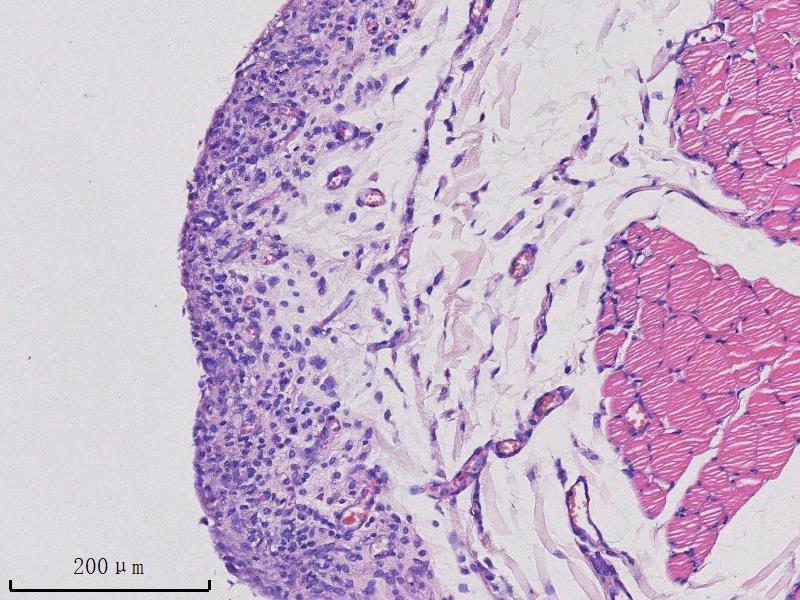

Supplement: S2 File — (ZIP) [file pone.0301540.s002.zip › raw data 2/HE/HE/54-10.jpg]

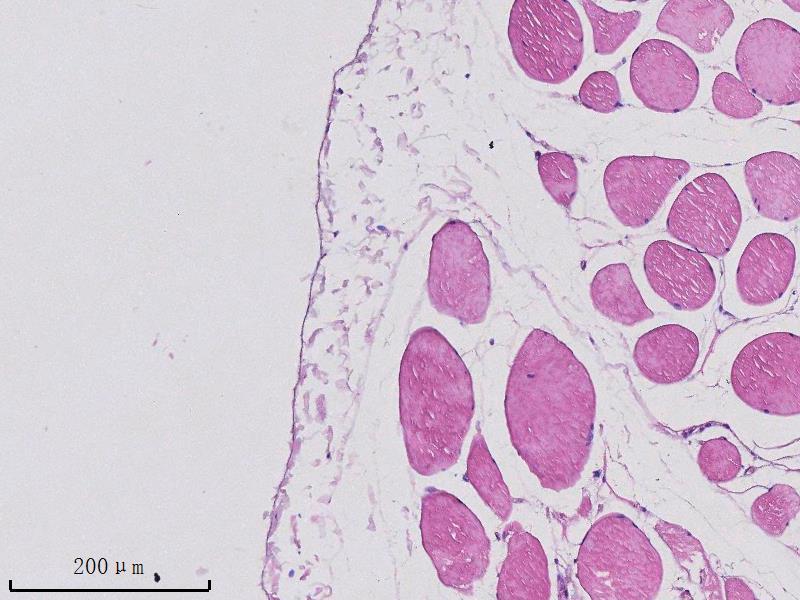

Supplement: S2 File — (ZIP) [file pone.0301540.s002.zip › raw data 2/HE/HE/7-10-1.jpg]

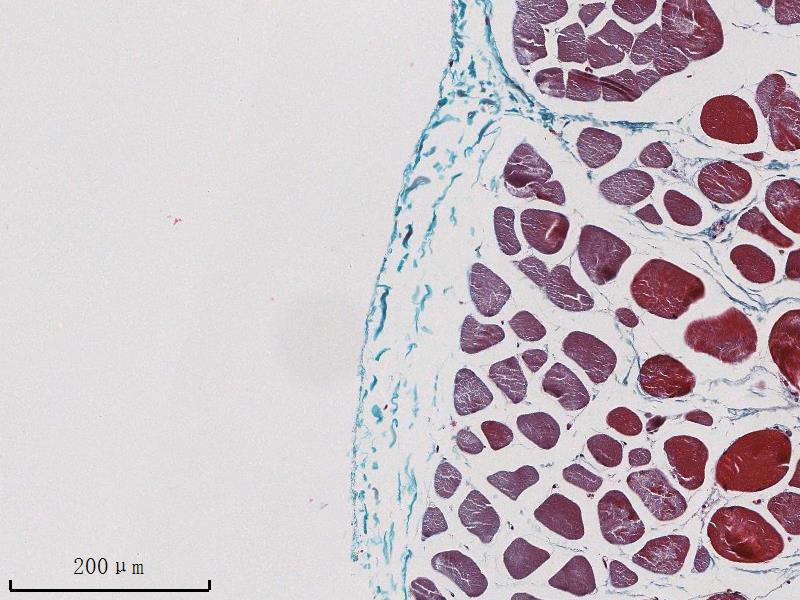

Supplement: S2 File — (ZIP) [file pone.0301540.s002.zip › raw data 2/MASSON/MASSON/masson/Control1.jpg]

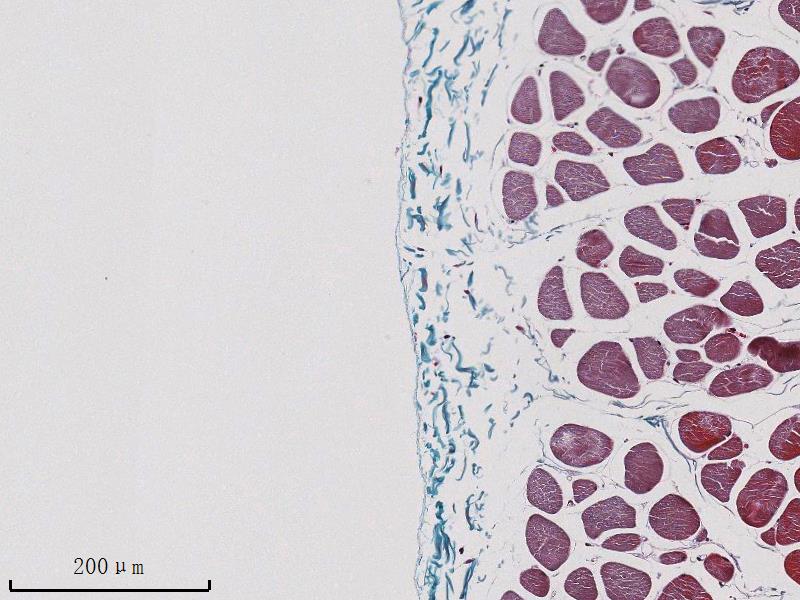

Supplement: S2 File — (ZIP) [file pone.0301540.s002.zip › raw data 2/MASSON/MASSON/masson/Control2.jpg]

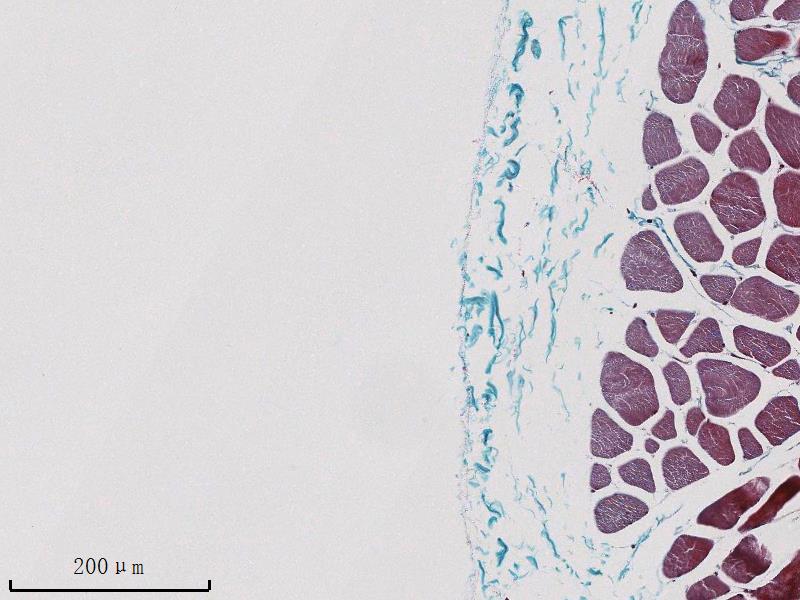

Supplement: S2 File — (ZIP) [file pone.0301540.s002.zip › raw data 2/MASSON/MASSON/masson/Control3.jpg]

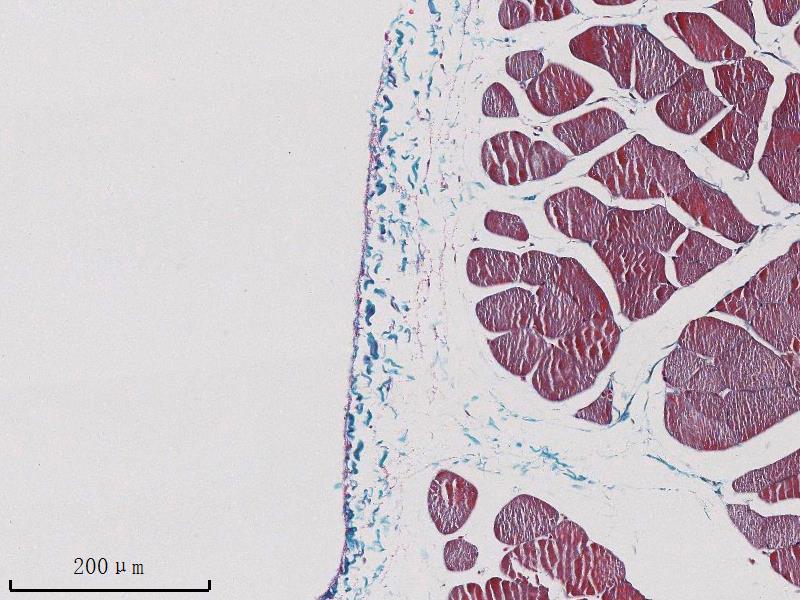

Supplement: S2 File — (ZIP) [file pone.0301540.s002.zip › raw data 2/MASSON/MASSON/masson/Control4.jpg]

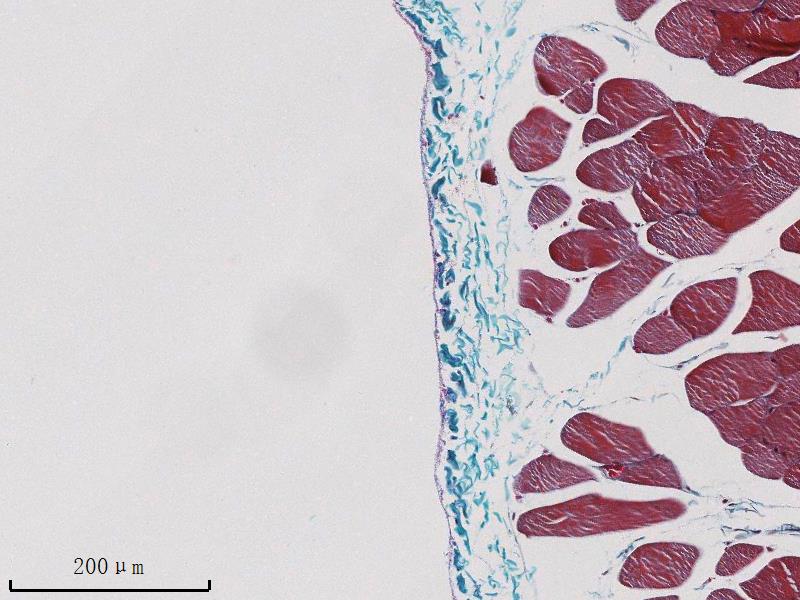

Supplement: S2 File — (ZIP) [file pone.0301540.s002.zip › raw data 2/MASSON/MASSON/masson/Control5.jpg]

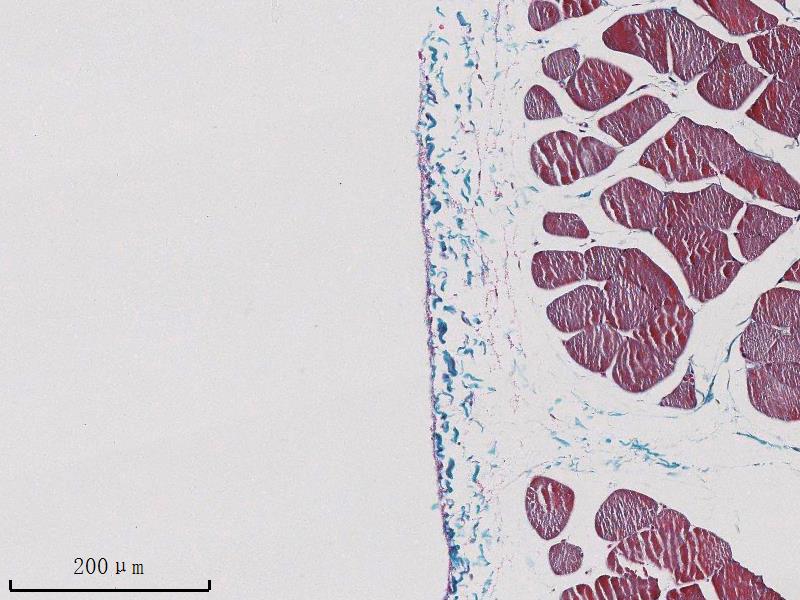

Supplement: S2 File — (ZIP) [file pone.0301540.s002.zip › raw data 2/MASSON/MASSON/masson/Control6.jpg]

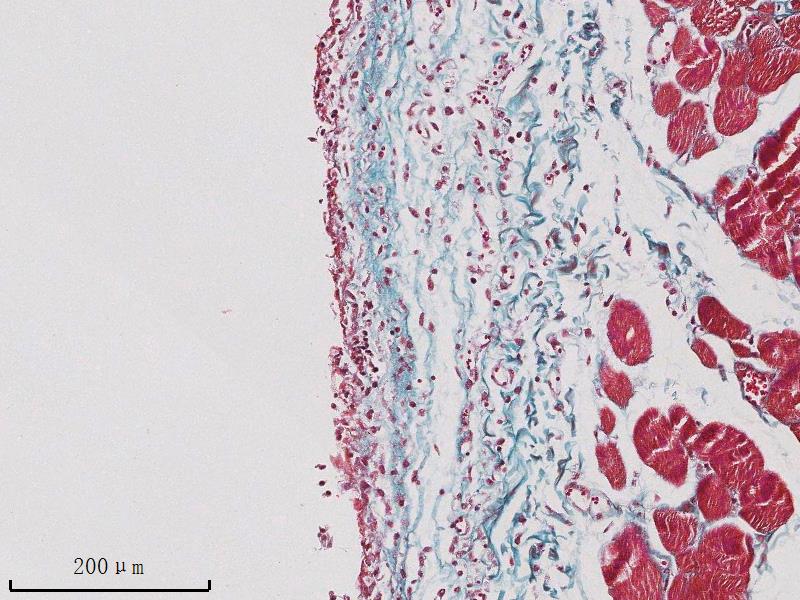

Supplement: S2 File — (ZIP) [file pone.0301540.s002.zip › raw data 2/MASSON/MASSON/masson/Losartan1.jpg]

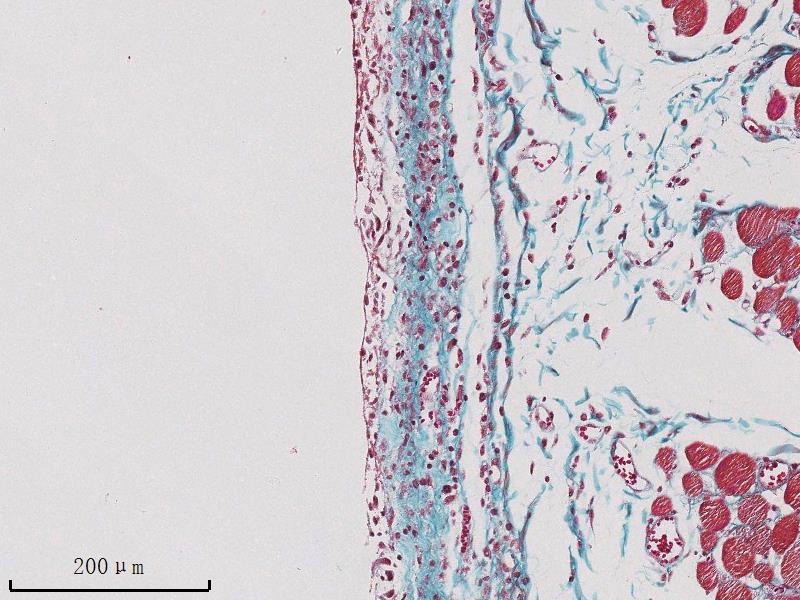

Supplement: S2 File — (ZIP) [file pone.0301540.s002.zip › raw data 2/MASSON/MASSON/masson/Losartan2.jpg]

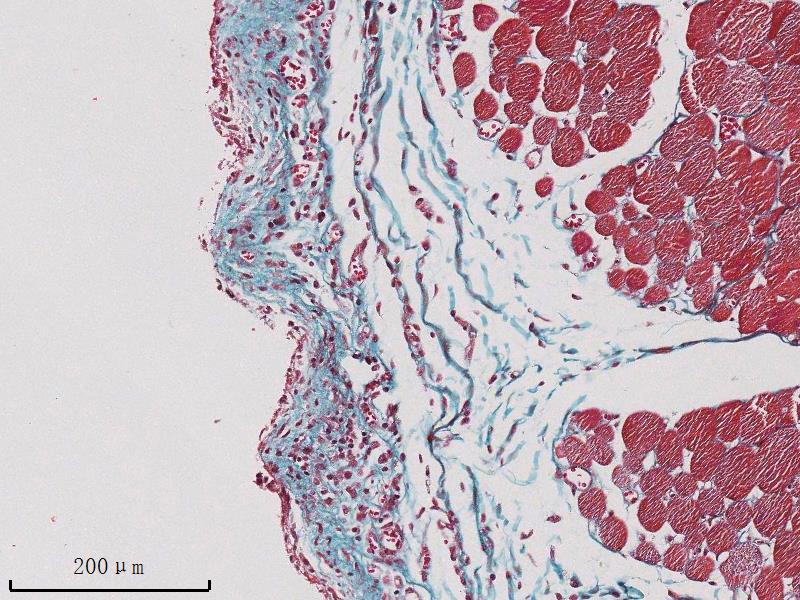

Supplement: S2 File — (ZIP) [file pone.0301540.s002.zip › raw data 2/MASSON/MASSON/masson/Losartan3.jpg]

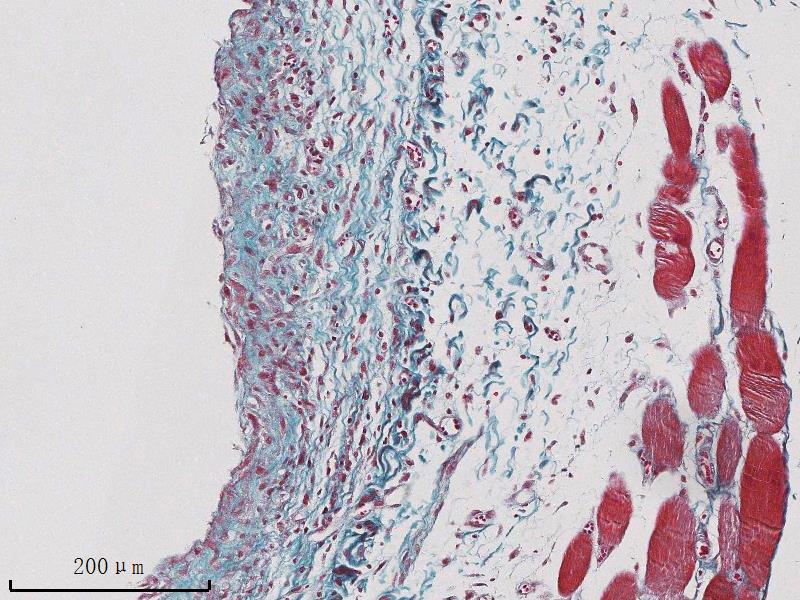

Supplement: S2 File — (ZIP) [file pone.0301540.s002.zip › raw data 2/MASSON/MASSON/masson/Losartan4.jpg]

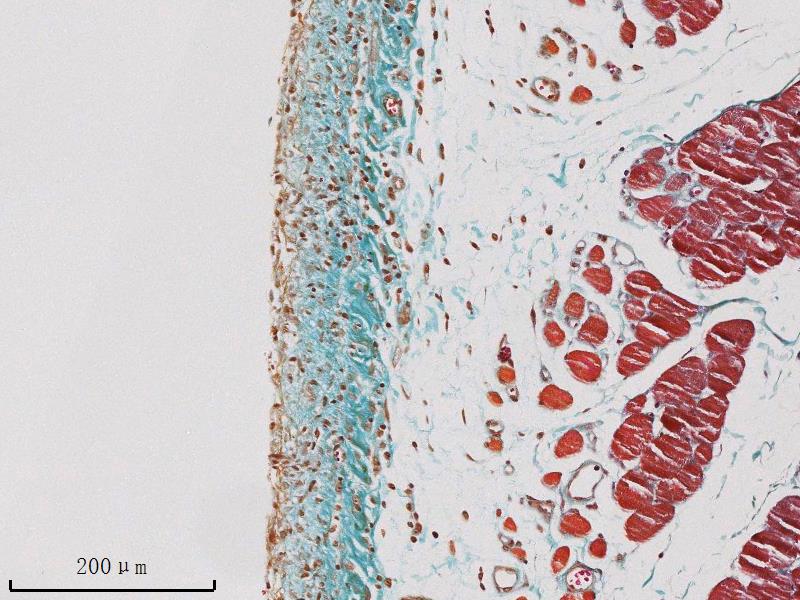

Supplement: S2 File — (ZIP) [file pone.0301540.s002.zip › raw data 2/MASSON/MASSON/masson/Losartan5.jpg]

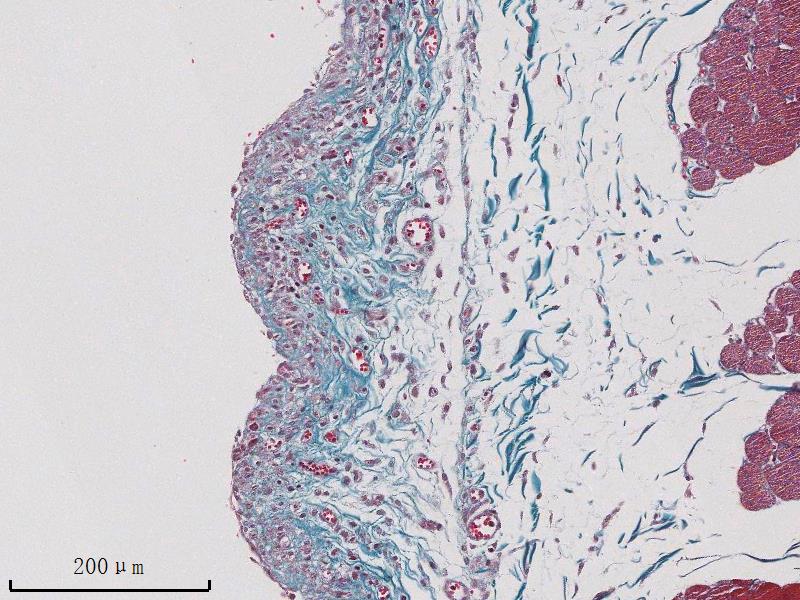

Supplement: S2 File — (ZIP) [file pone.0301540.s002.zip › raw data 2/MASSON/MASSON/masson/Losartan6.jpg]

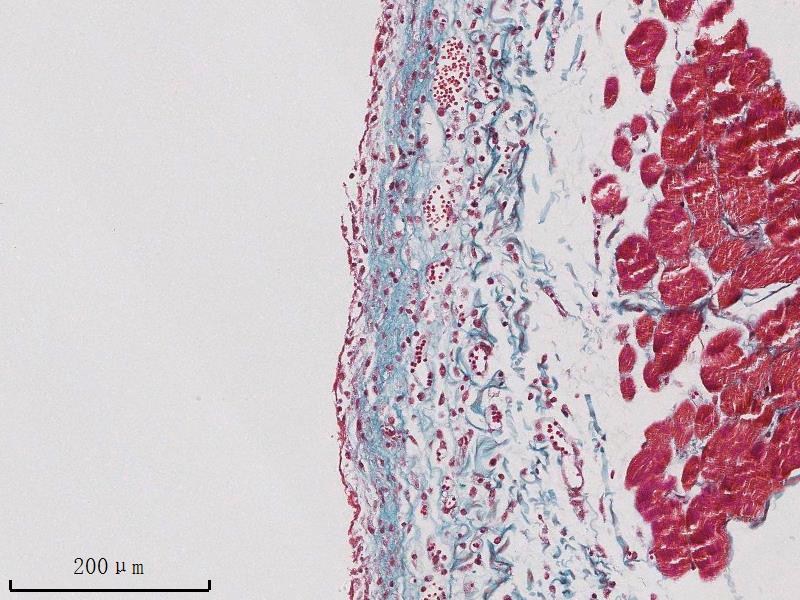

Supplement: S2 File — (ZIP) [file pone.0301540.s002.zip › raw data 2/MASSON/MASSON/masson/PF1.jpg]

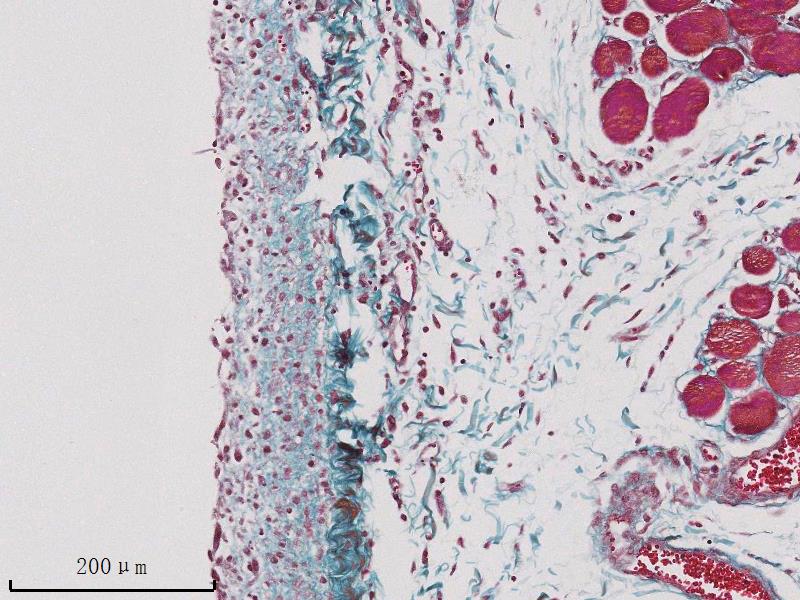

Supplement: S2 File — (ZIP) [file pone.0301540.s002.zip › raw data 2/MASSON/MASSON/masson/PF2.jpg]

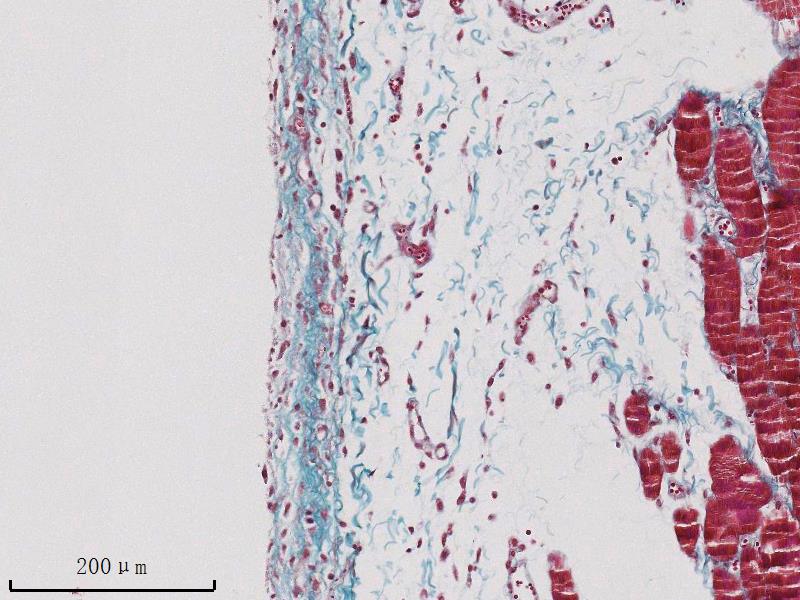

Supplement: S2 File — (ZIP) [file pone.0301540.s002.zip › raw data 2/MASSON/MASSON/masson/PF3.jpg]

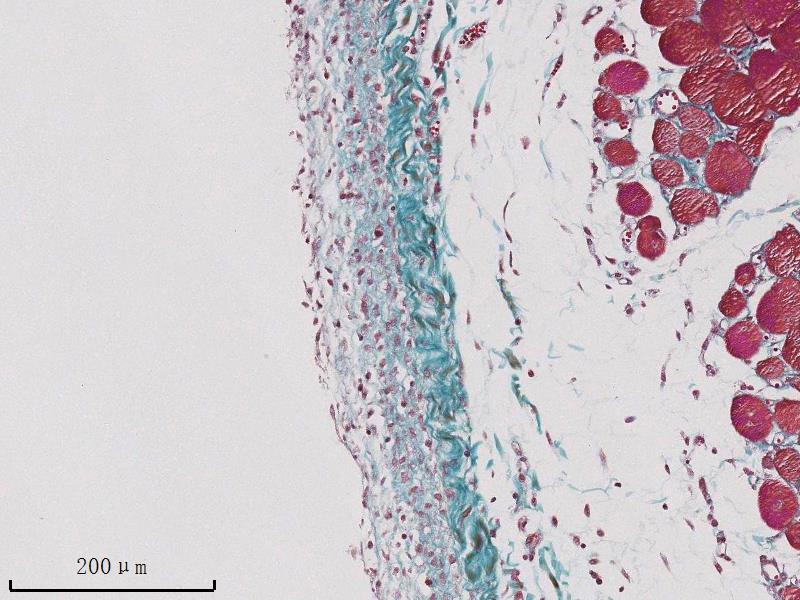

Supplement: S2 File — (ZIP) [file pone.0301540.s002.zip › raw data 2/MASSON/MASSON/masson/PF4.jpg]

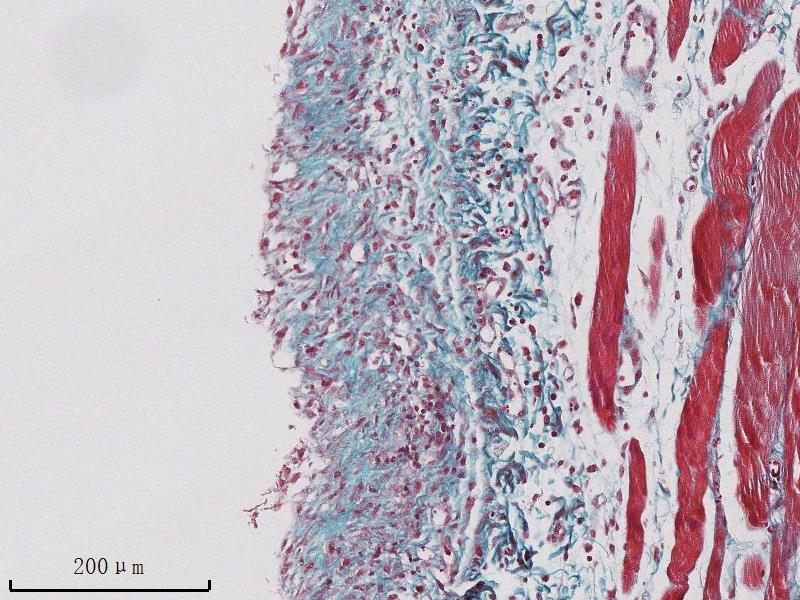

Supplement: S2 File — (ZIP) [file pone.0301540.s002.zip › raw data 2/MASSON/MASSON/masson/PF5.jpg]

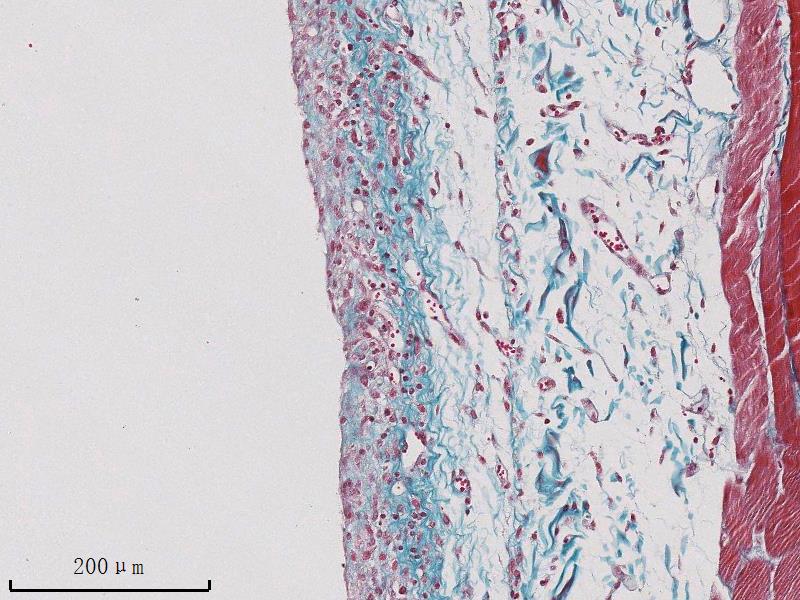

Supplement: S2 File — (ZIP) [file pone.0301540.s002.zip › raw data 2/MASSON/MASSON/masson/PF6.jpg]

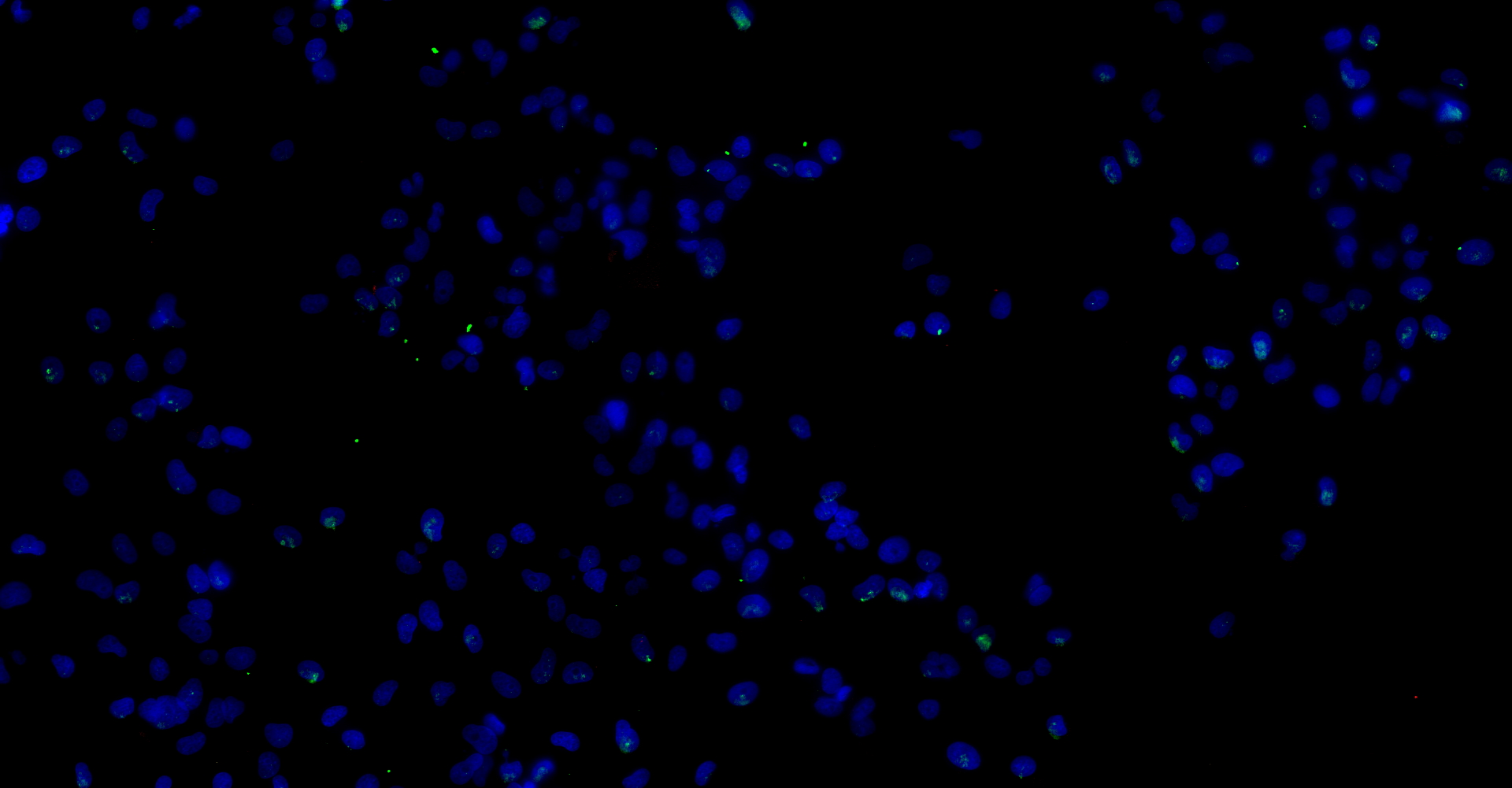

Supplement: S3 File — (ZIP) [file pone.0301540.s003.zip › S3 raw date 3/Fluorescence in situ hybridisation/zutu/a-sma-smad2/cont/AN 全 1.jpg]

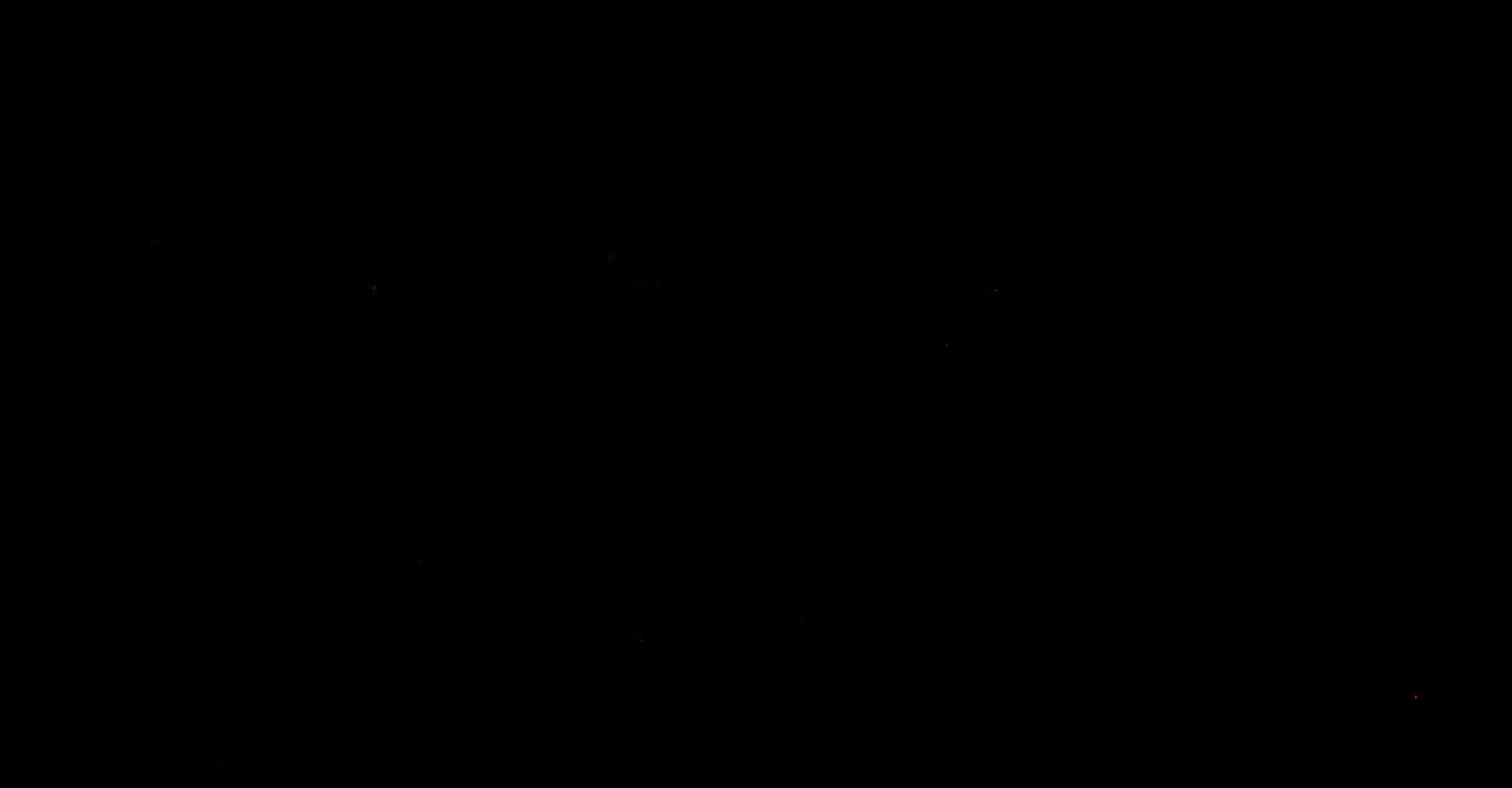

Supplement: S3 File — (ZIP) [file pone.0301540.s003.zip › S3 raw date 3/Fluorescence in situ hybridisation/zutu/a-sma-smad2/cont/AN 红 1.jpg]

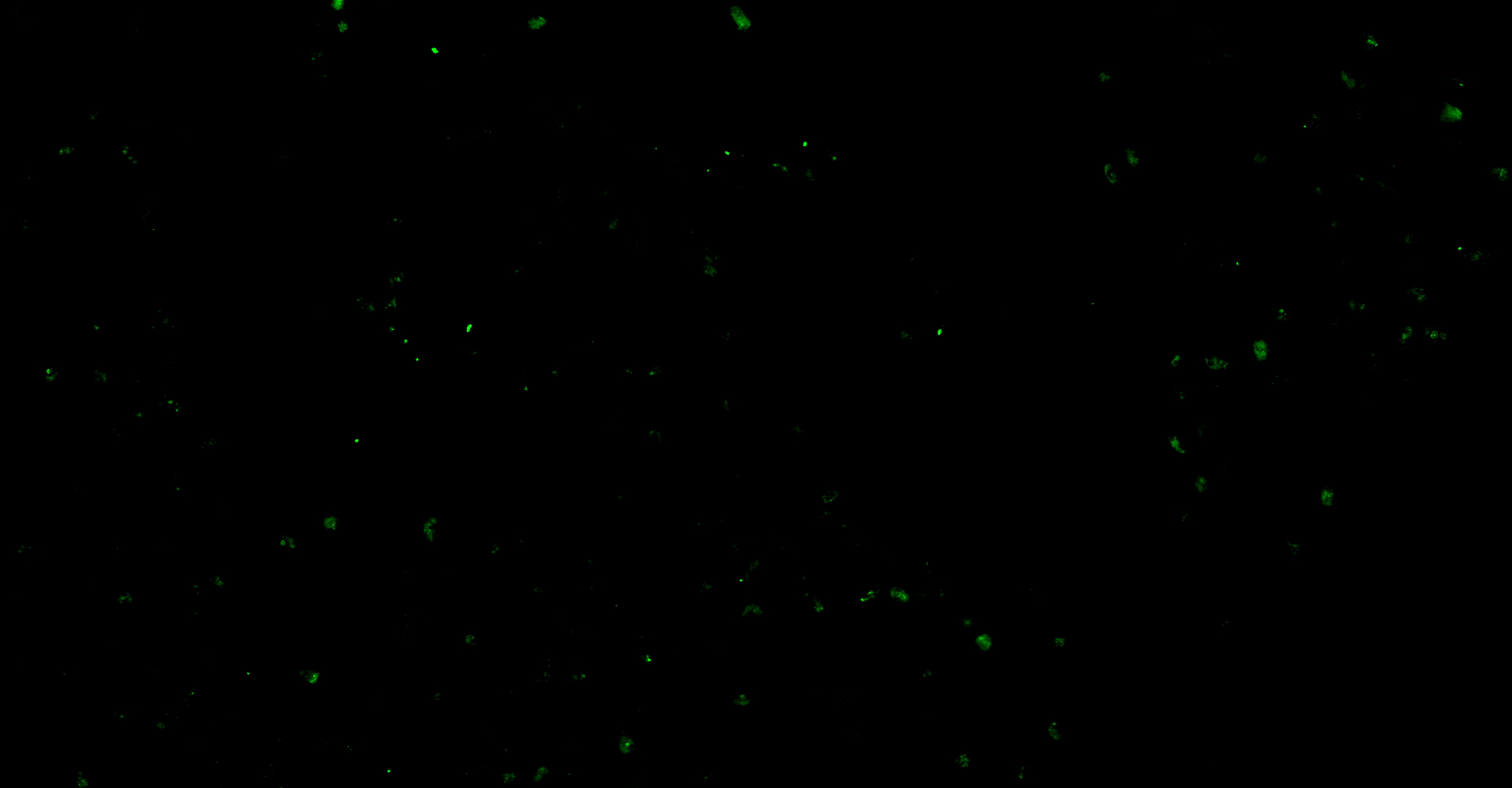

Supplement: S3 File — (ZIP) [file pone.0301540.s003.zip › S3 raw date 3/Fluorescence in situ hybridisation/zutu/a-sma-smad2/cont/AN 绿 1.jpg]

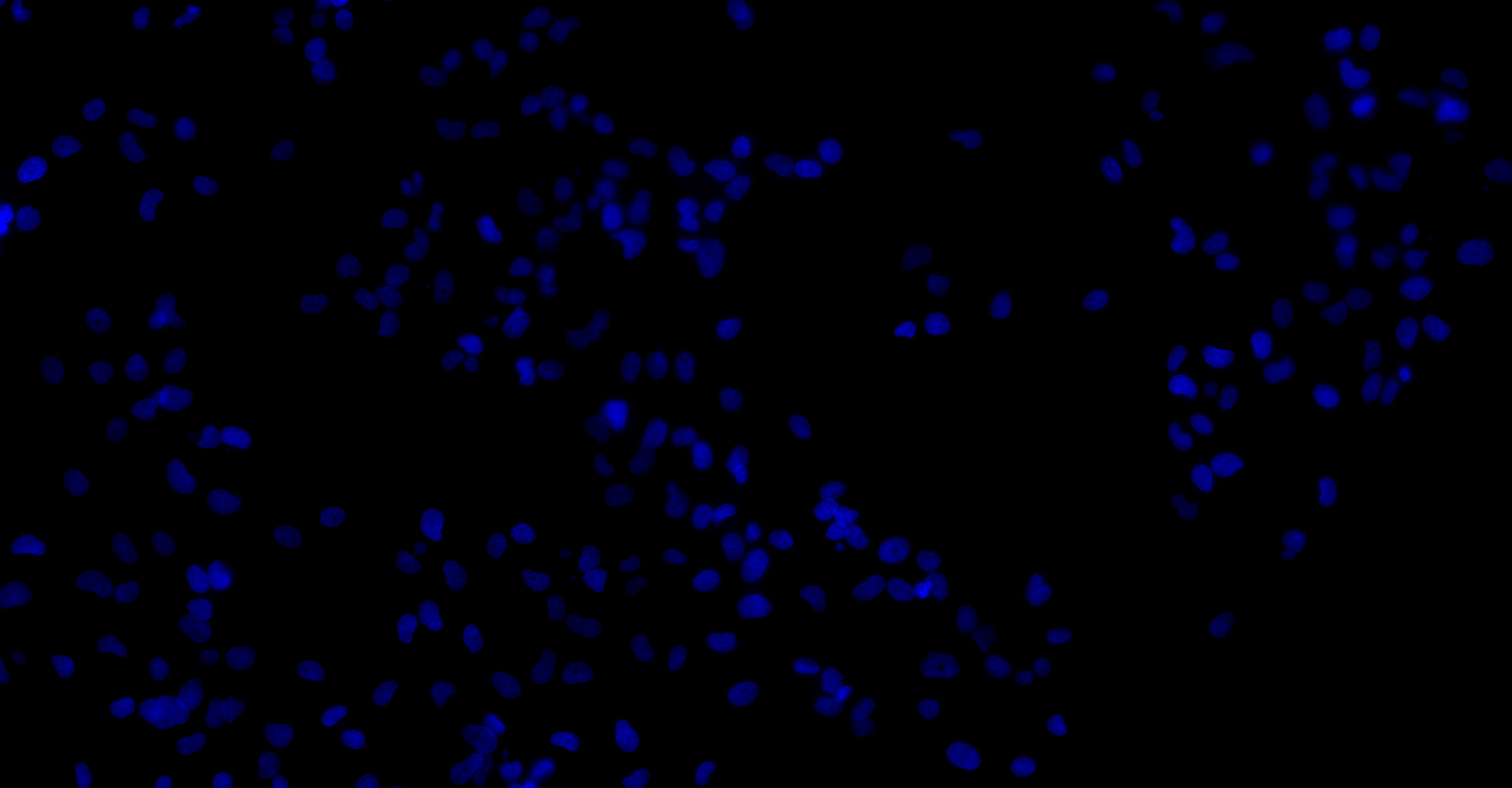

Supplement: S3 File — (ZIP) [file pone.0301540.s003.zip › S3 raw date 3/Fluorescence in situ hybridisation/zutu/a-sma-smad2/cont/AN 蓝 1.jpg]

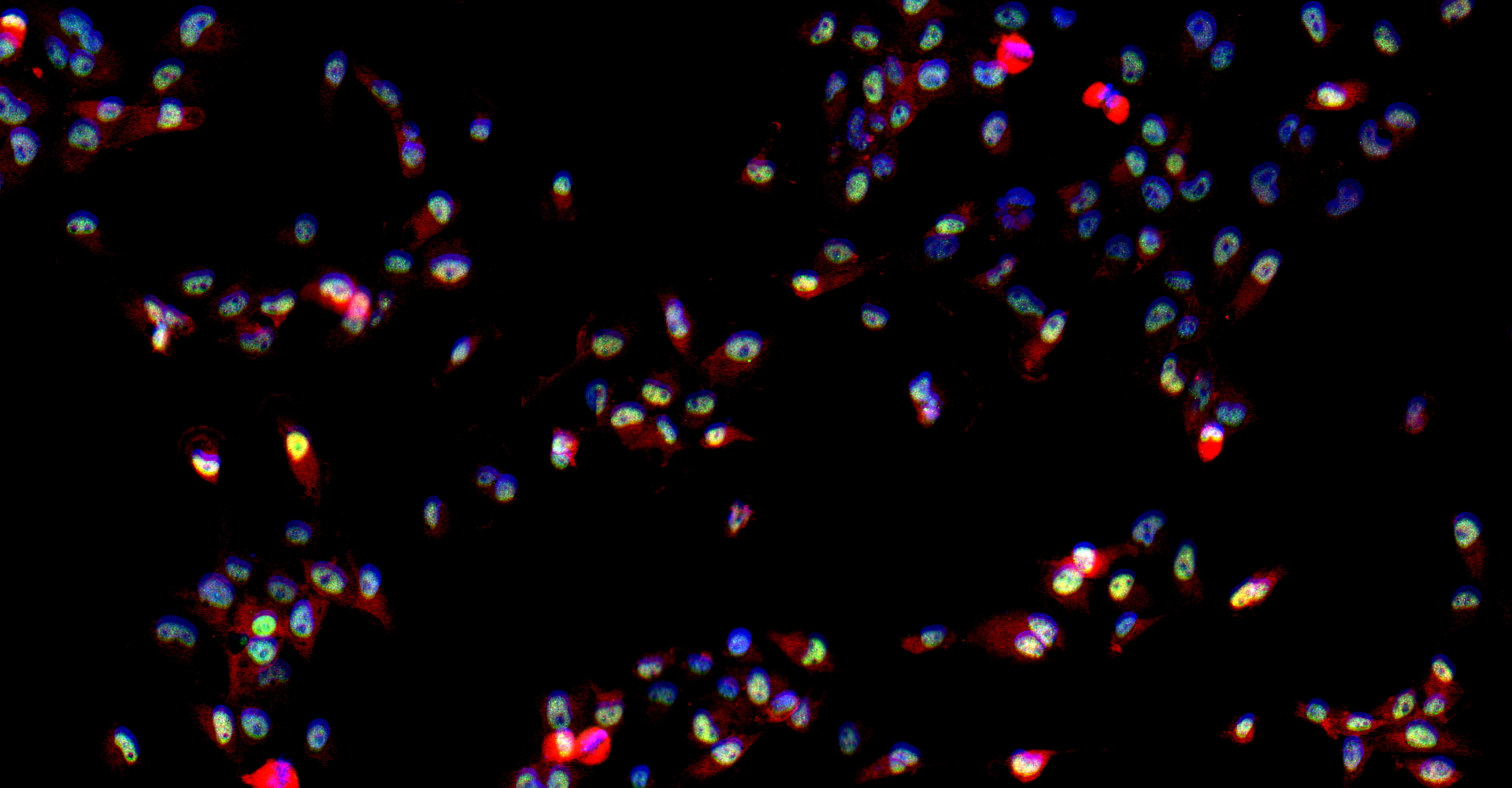

Supplement: S3 File — (ZIP) [file pone.0301540.s003.zip › S3 raw date 3/Fluorescence in situ hybridisation/zutu/a-sma-smad2/in/B2M 全 2.jpg]

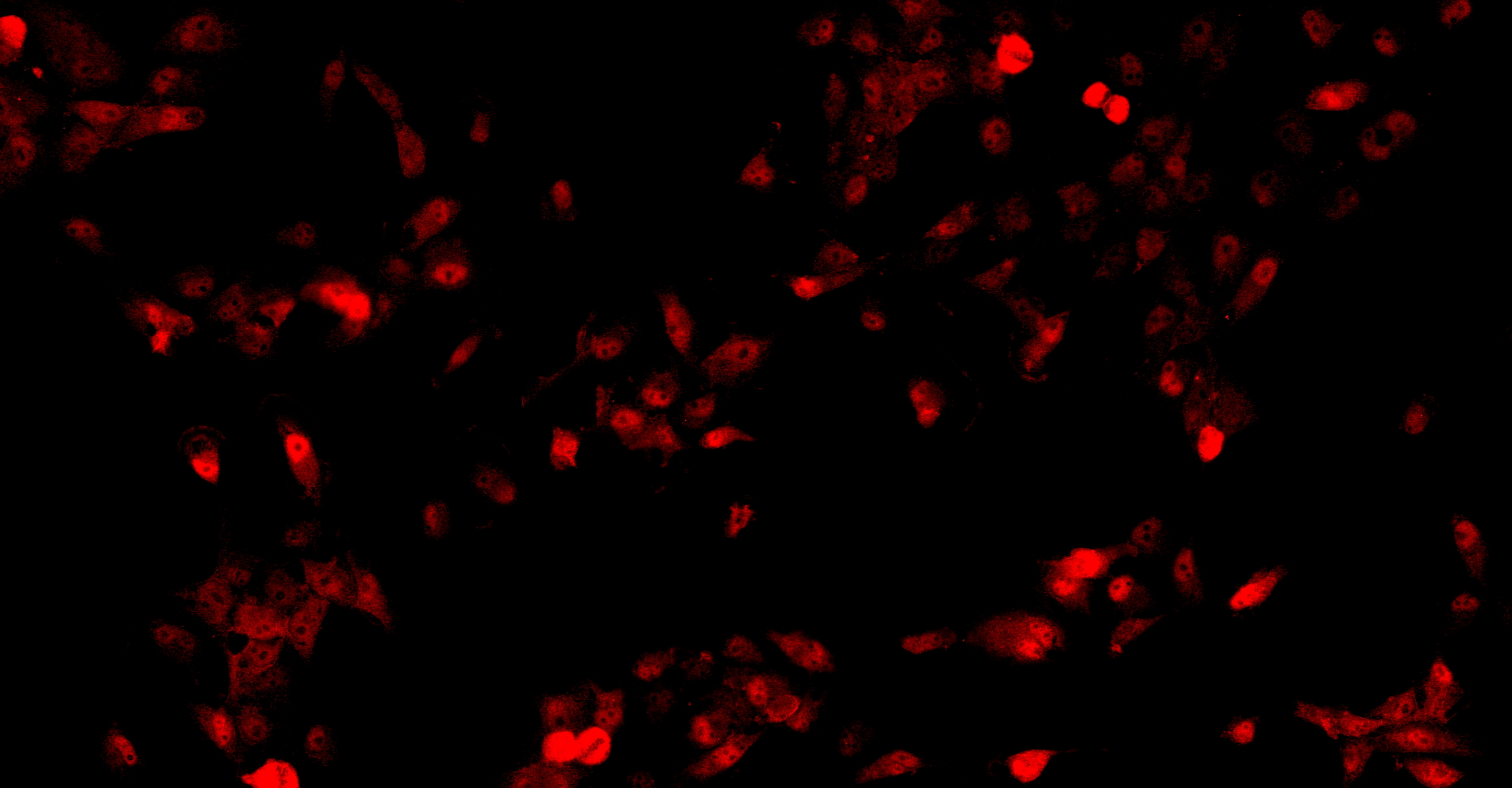

Supplement: S3 File — (ZIP) [file pone.0301540.s003.zip › S3 raw date 3/Fluorescence in situ hybridisation/zutu/a-sma-smad2/in/B2M 红 2.jpg]

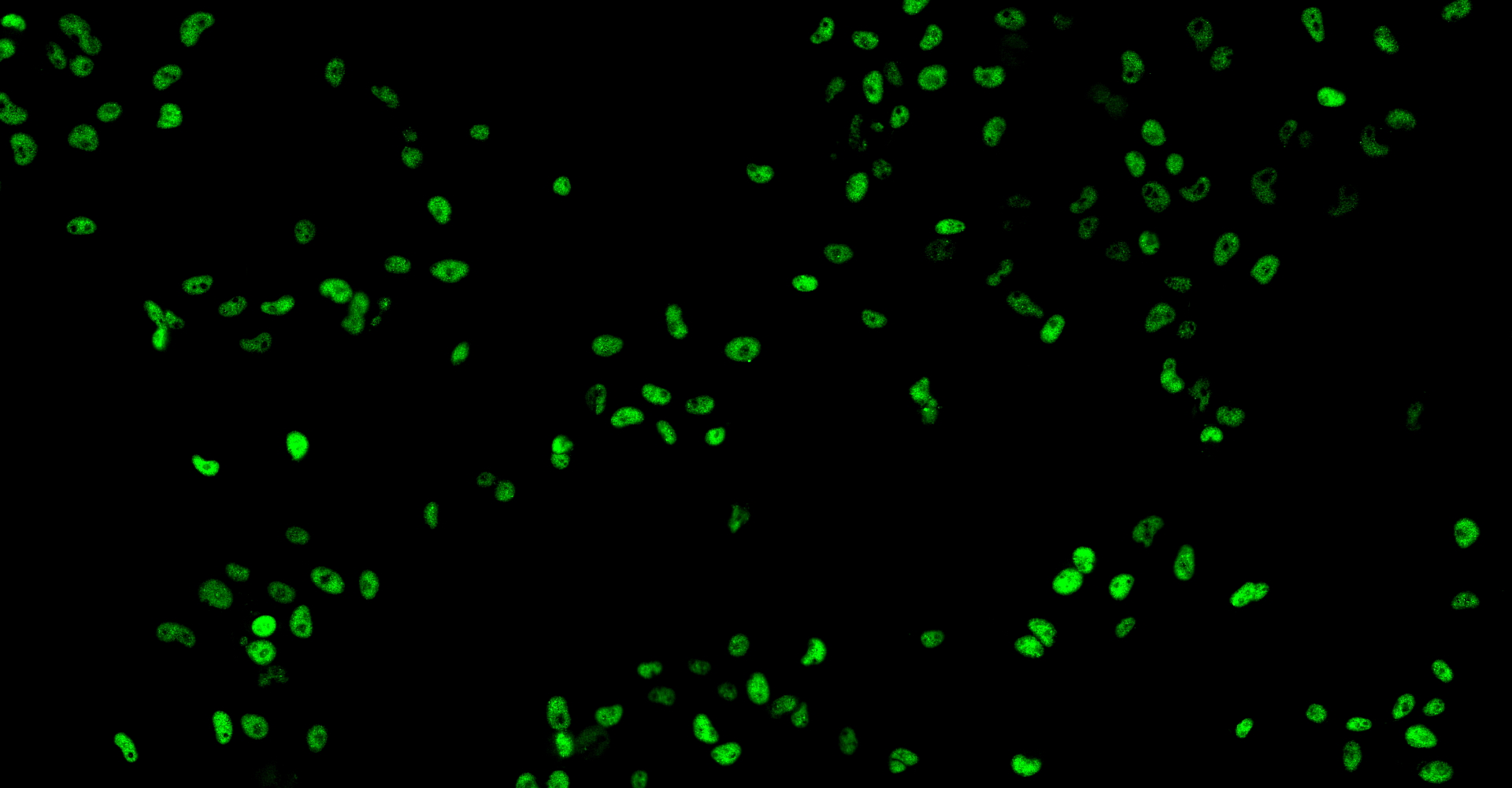

Supplement: S3 File — (ZIP) [file pone.0301540.s003.zip › S3 raw date 3/Fluorescence in situ hybridisation/zutu/a-sma-smad2/in/B2M 绿 2.jpg]

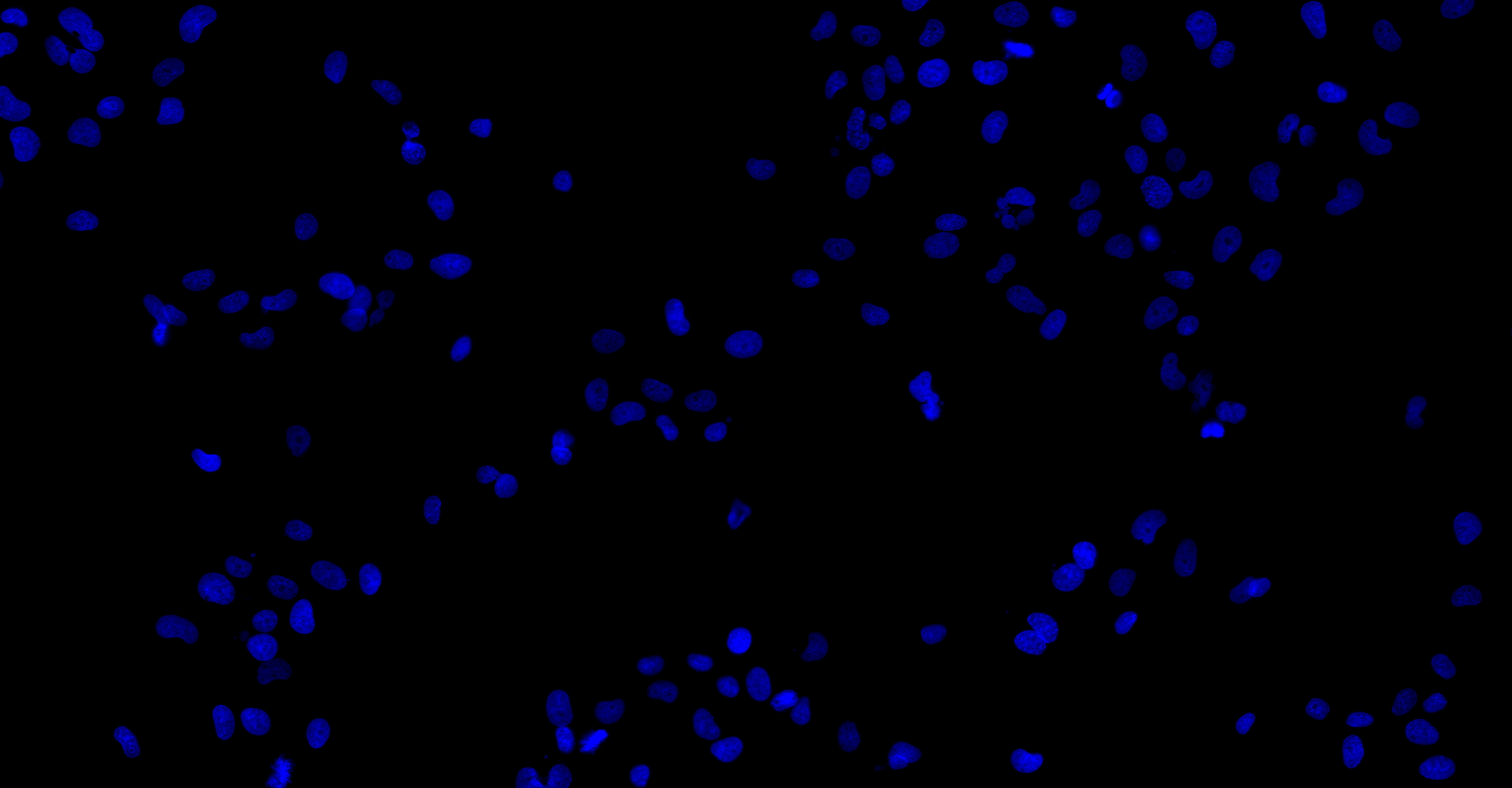

Supplement: S3 File — (ZIP) [file pone.0301540.s003.zip › S3 raw date 3/Fluorescence in situ hybridisation/zutu/a-sma-smad2/in/B2M 蓝 2.jpg]

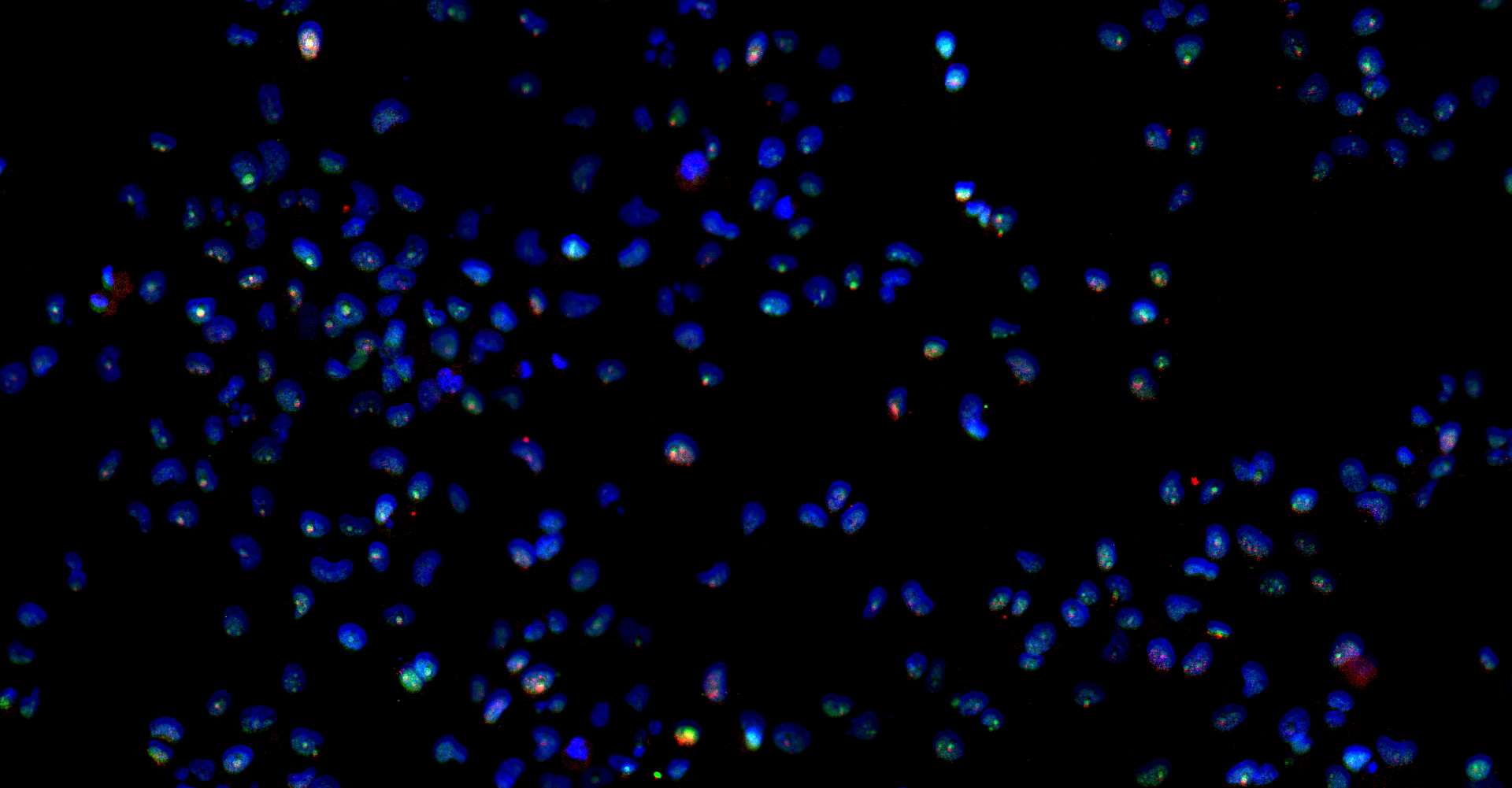

Supplement: S3 File — (ZIP) [file pone.0301540.s003.zip › S3 raw date 3/Fluorescence in situ hybridisation/zutu/a-sma-smad2/mi/A2D 全 2.jpg]

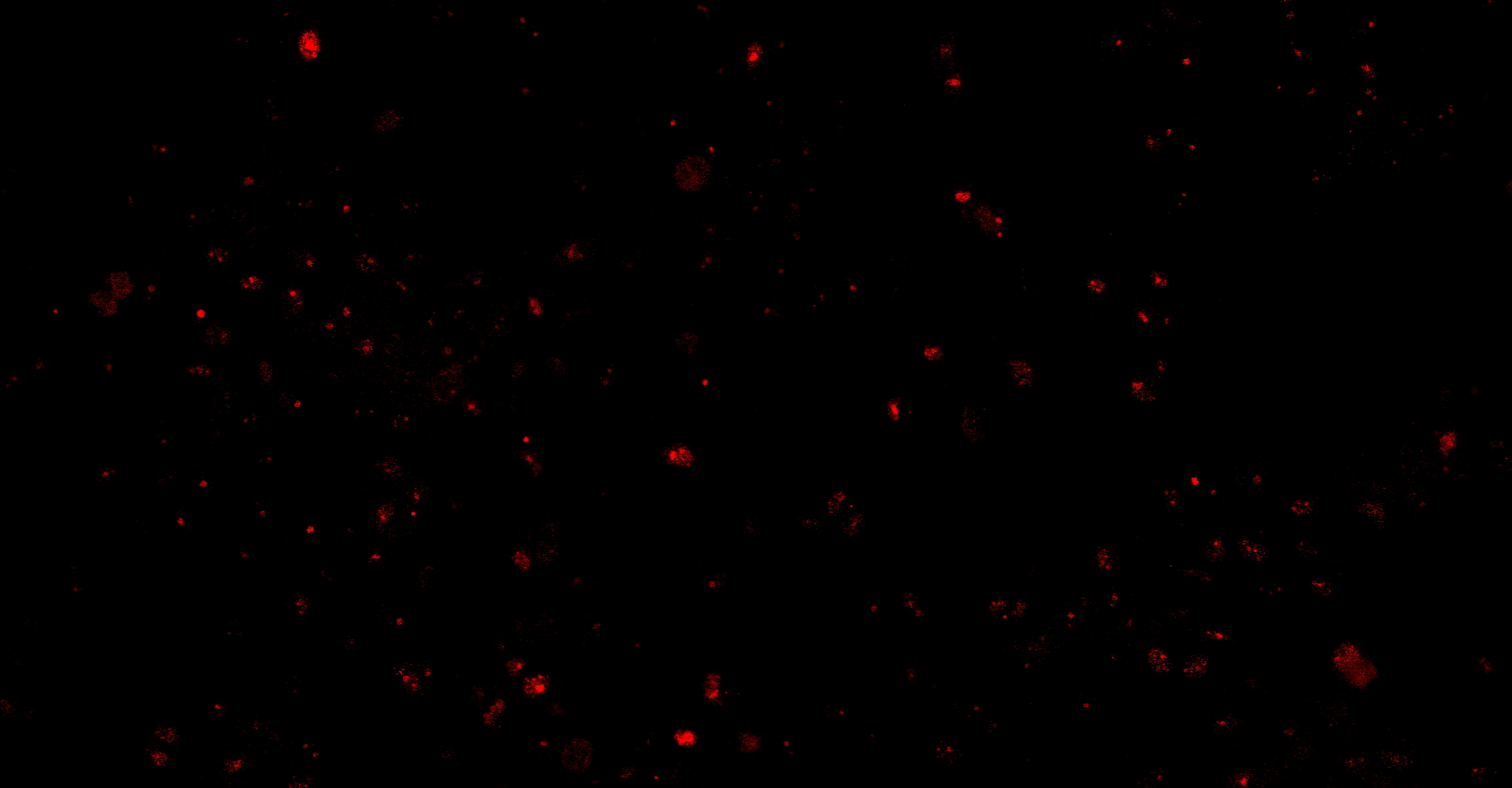

Supplement: S3 File — (ZIP) [file pone.0301540.s003.zip › S3 raw date 3/Fluorescence in situ hybridisation/zutu/a-sma-smad2/mi/A2D 红 2.jpg]

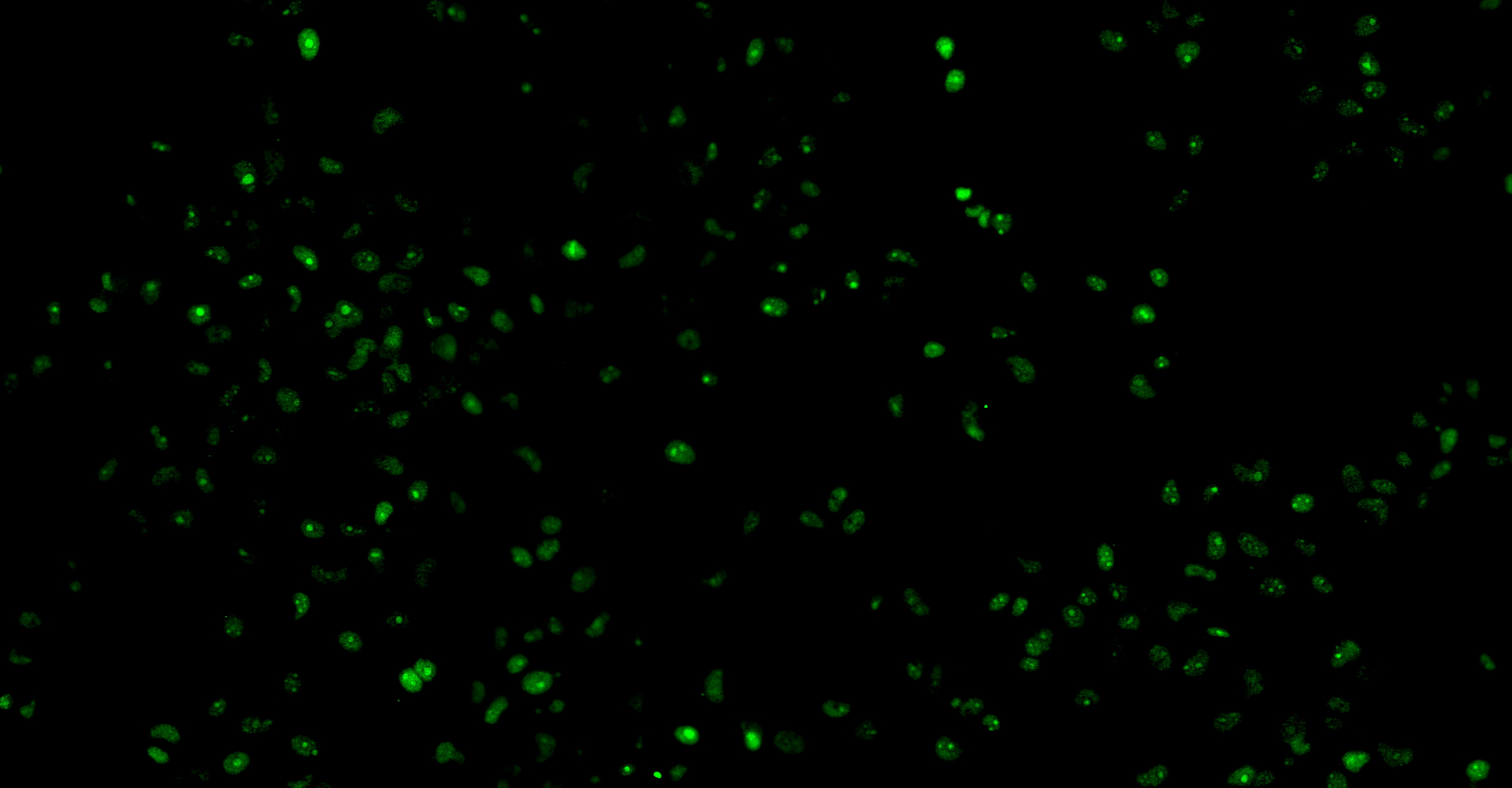

Supplement: S3 File — (ZIP) [file pone.0301540.s003.zip › S3 raw date 3/Fluorescence in situ hybridisation/zutu/a-sma-smad2/mi/A2D 绿 2.jpg]

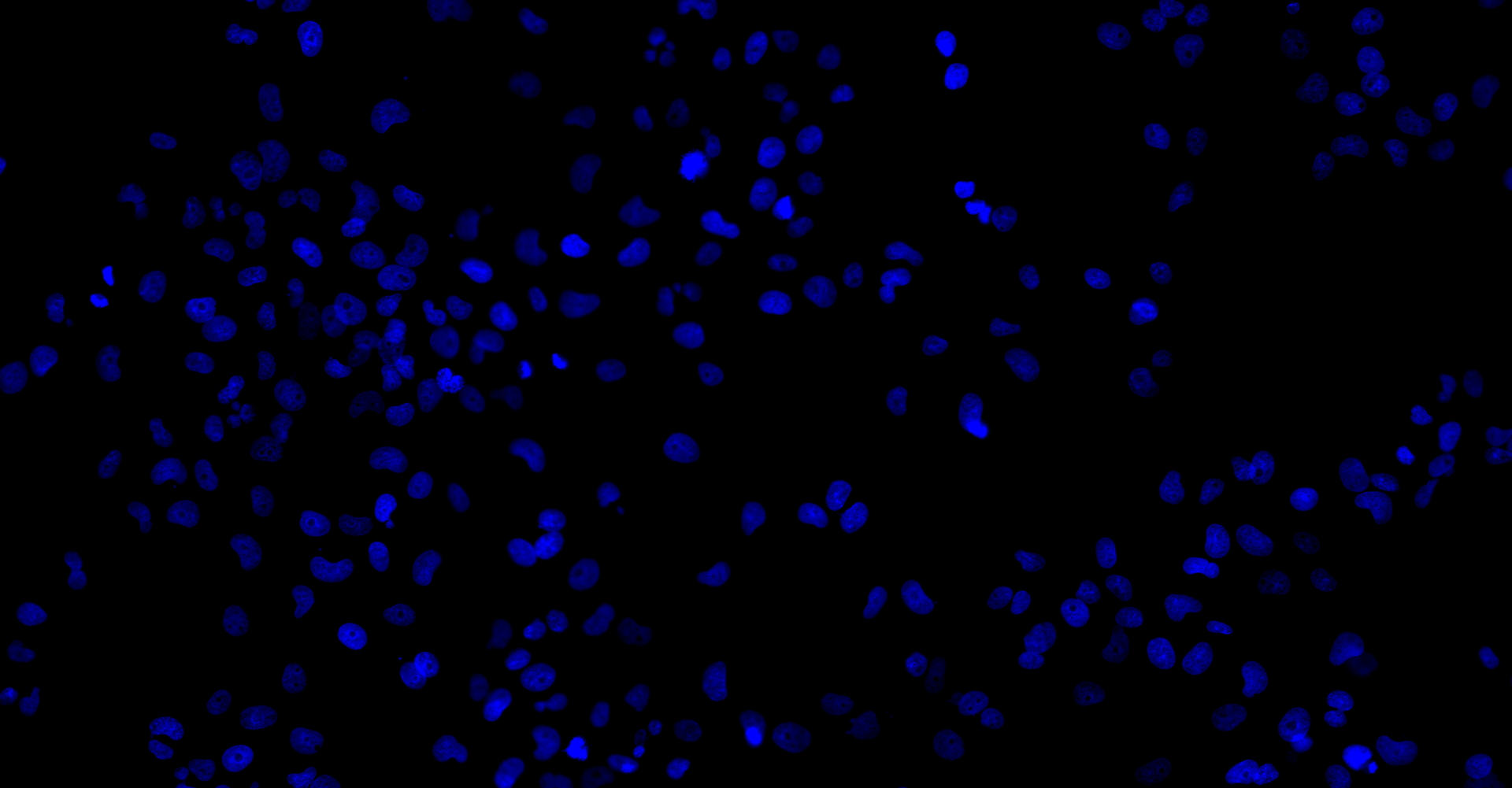

Supplement: S3 File — (ZIP) [file pone.0301540.s003.zip › S3 raw date 3/Fluorescence in situ hybridisation/zutu/a-sma-smad2/mi/A2D 蓝 2.jpg]

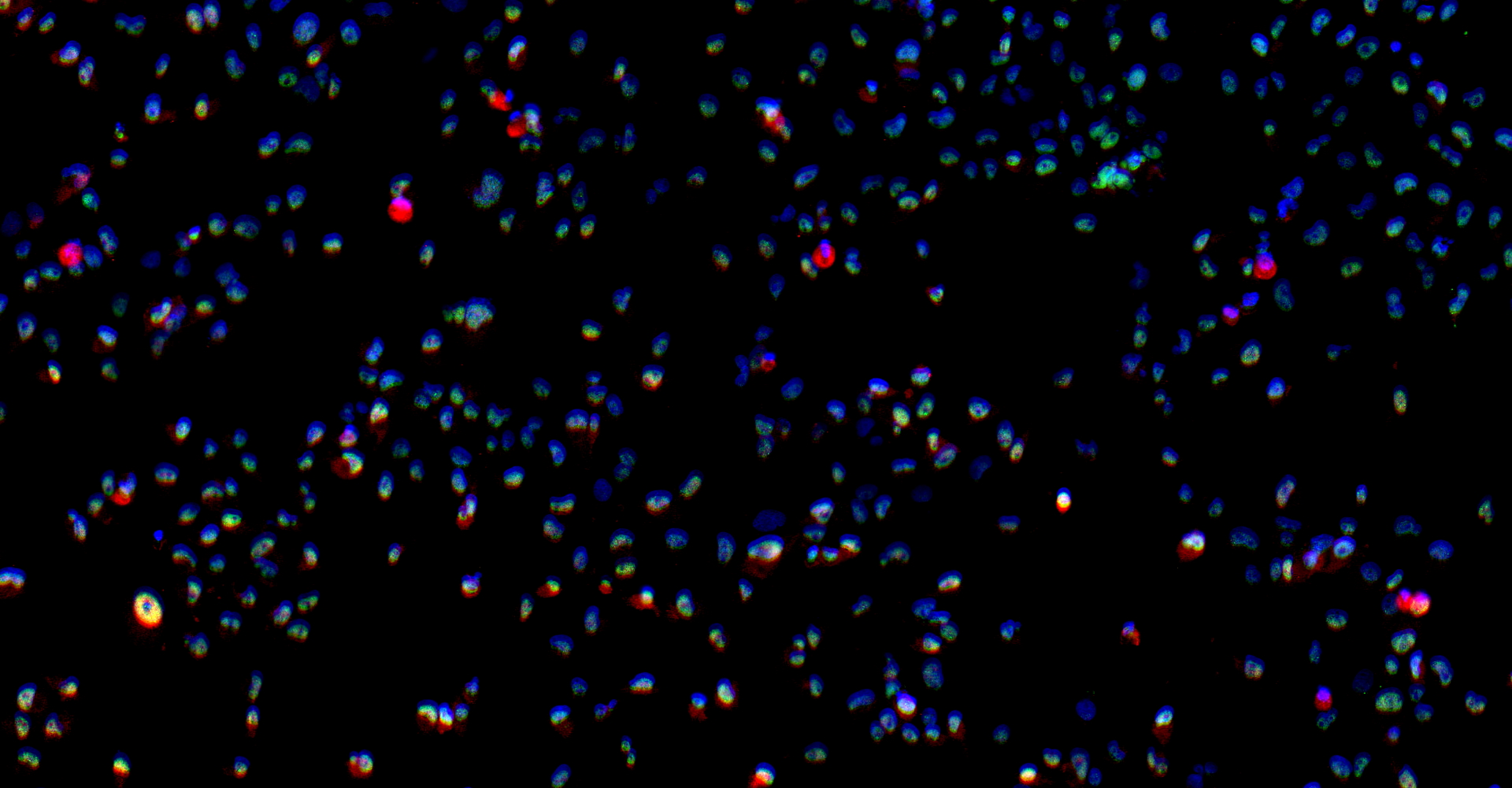

Supplement: S3 File — (ZIP) [file pone.0301540.s003.zip › S3 raw date 3/Fluorescence in situ hybridisation/zutu/a-sma-smad2/tgf/B2D 全 1.jpg]

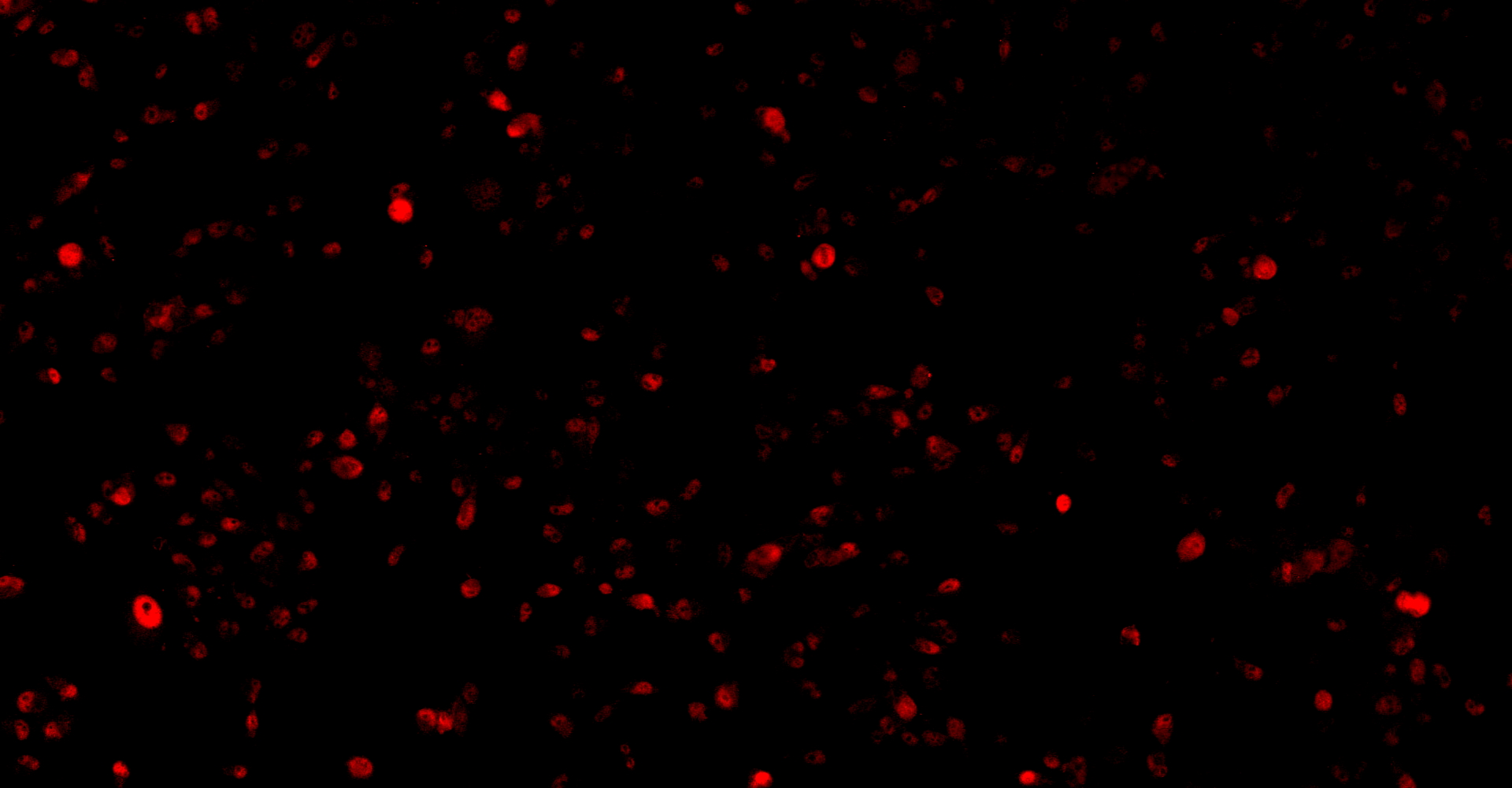

Supplement: S3 File — (ZIP) [file pone.0301540.s003.zip › S3 raw date 3/Fluorescence in situ hybridisation/zutu/a-sma-smad2/tgf/B2D 红 1.jpg]

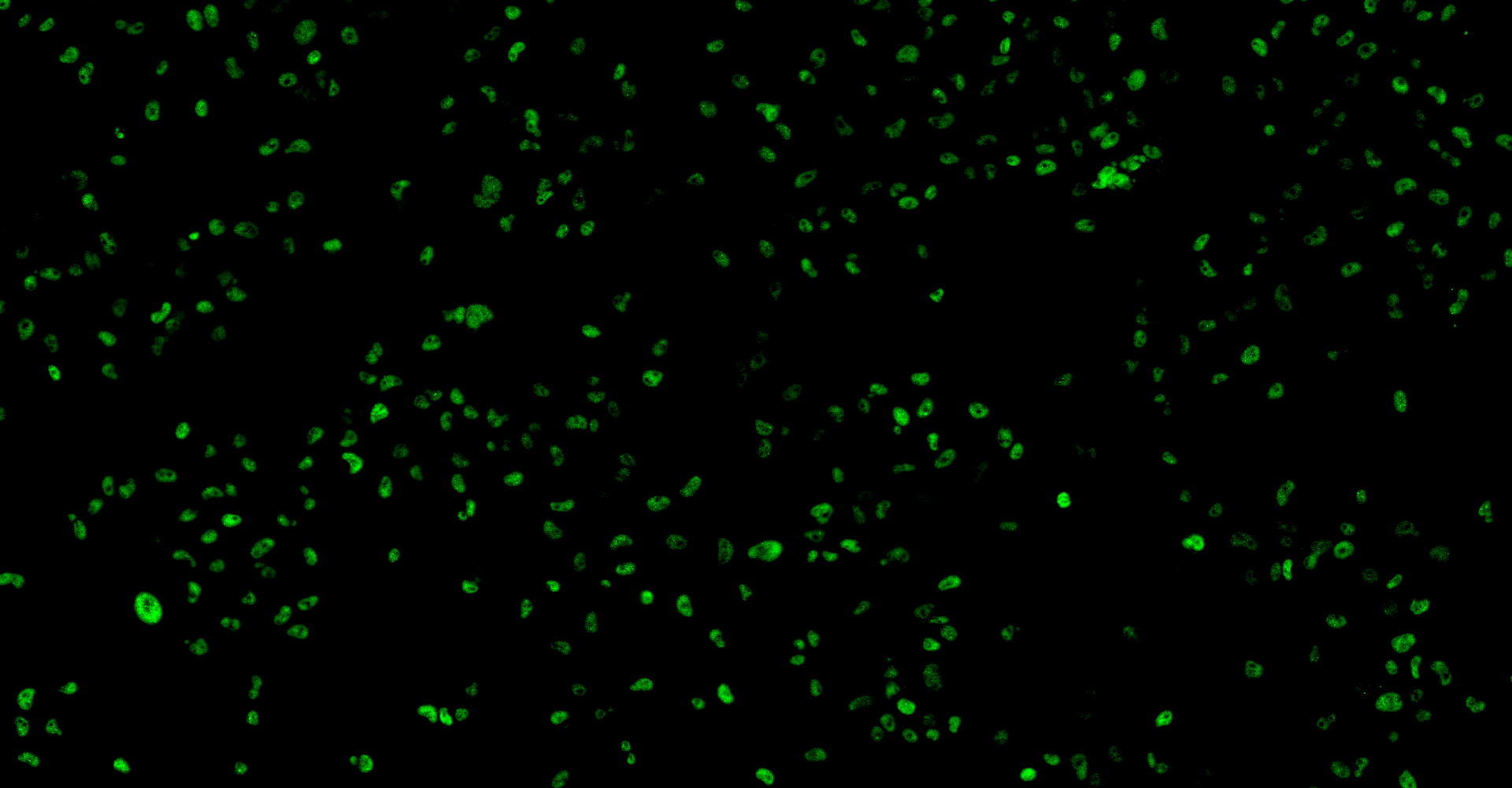

Supplement: S3 File — (ZIP) [file pone.0301540.s003.zip › S3 raw date 3/Fluorescence in situ hybridisation/zutu/a-sma-smad2/tgf/B2D 绿 1.jpg]

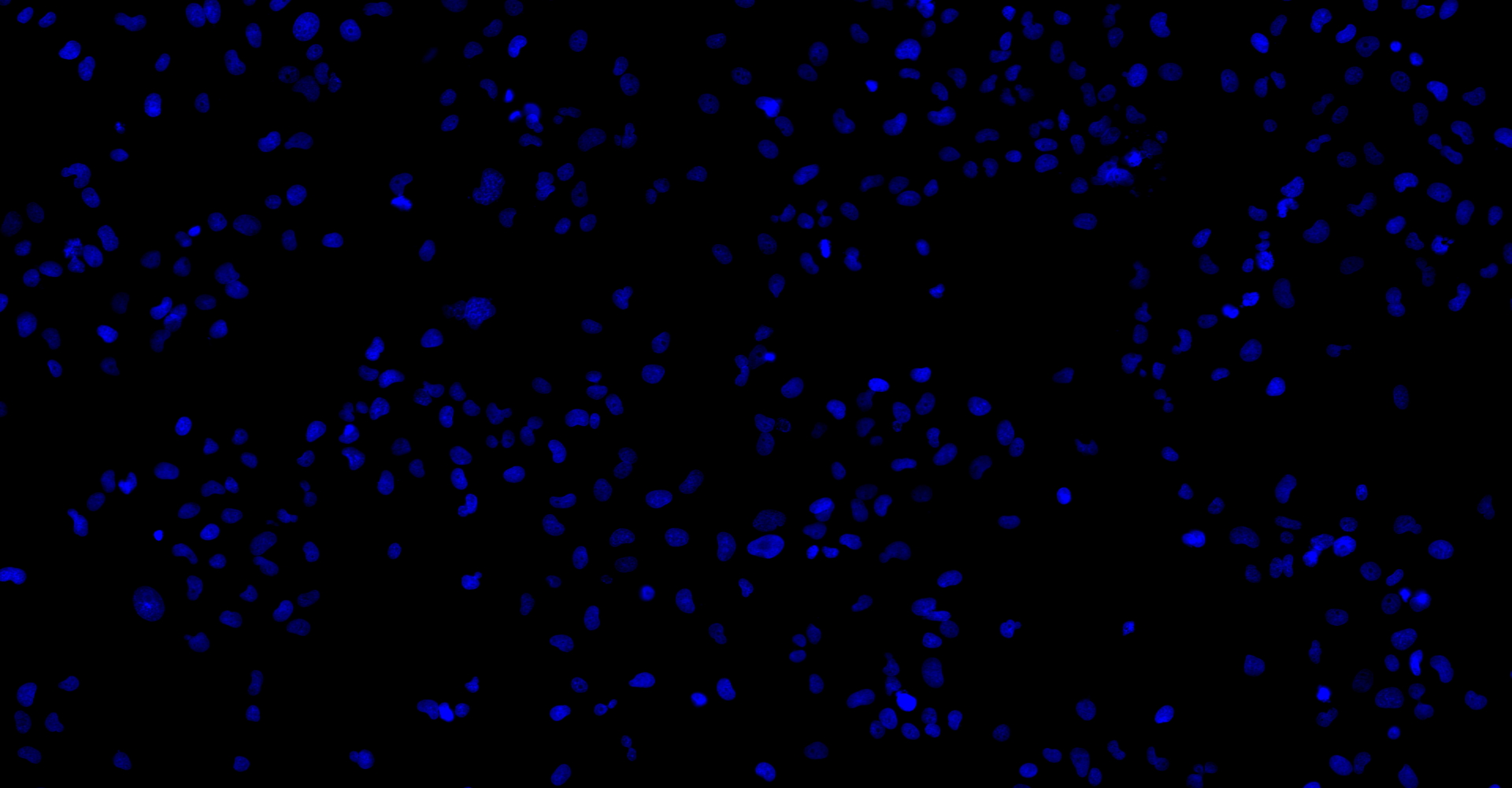

Supplement: S3 File — (ZIP) [file pone.0301540.s003.zip › S3 raw date 3/Fluorescence in situ hybridisation/zutu/a-sma-smad2/tgf/B2D 蓝 1.jpg]

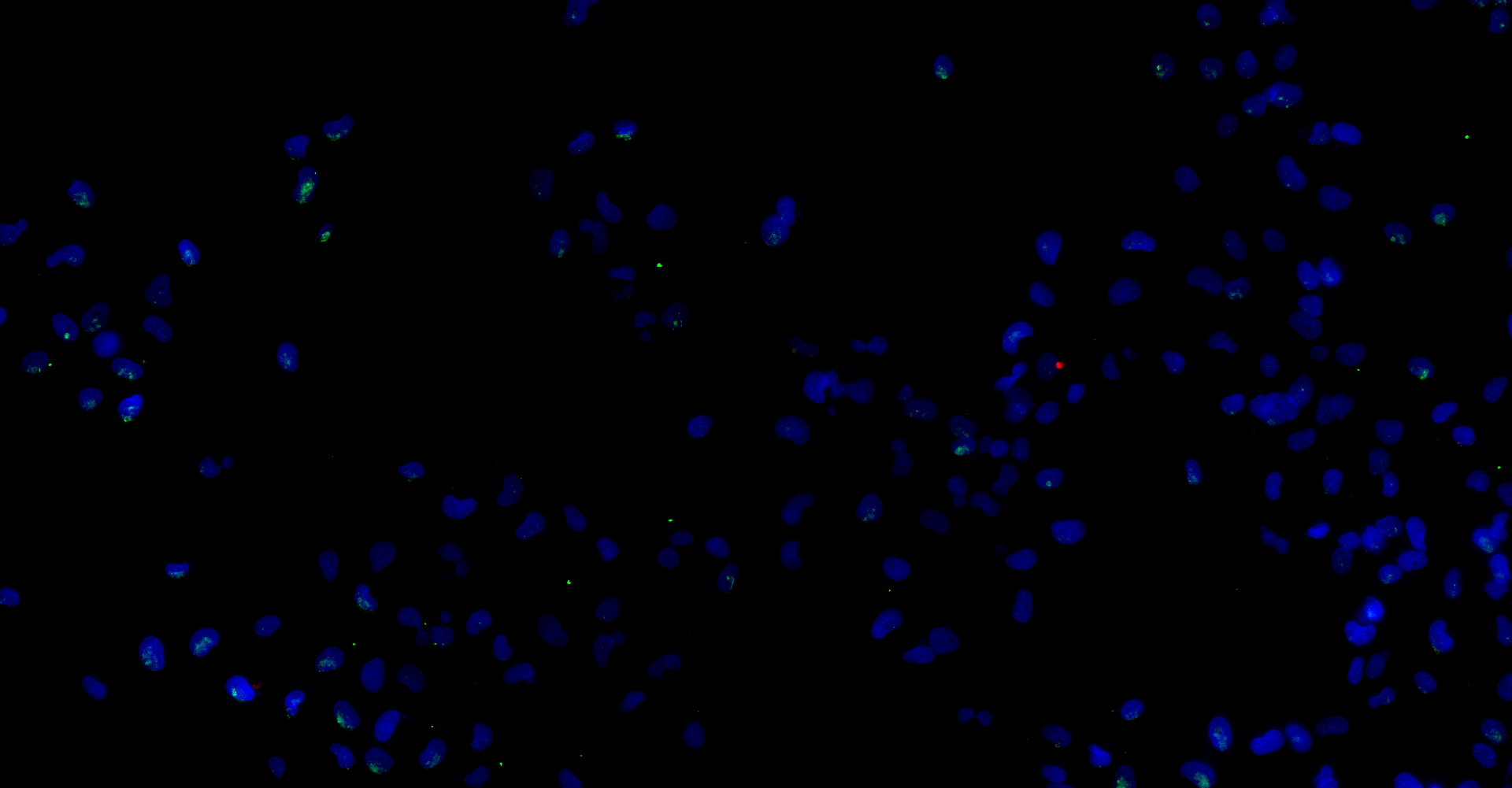

Supplement: S3 File — (ZIP) [file pone.0301540.s003.zip › S3 raw date 3/Fluorescence in situ hybridisation/zutu/tgf-smad3/cont/AN 全 2.jpg]

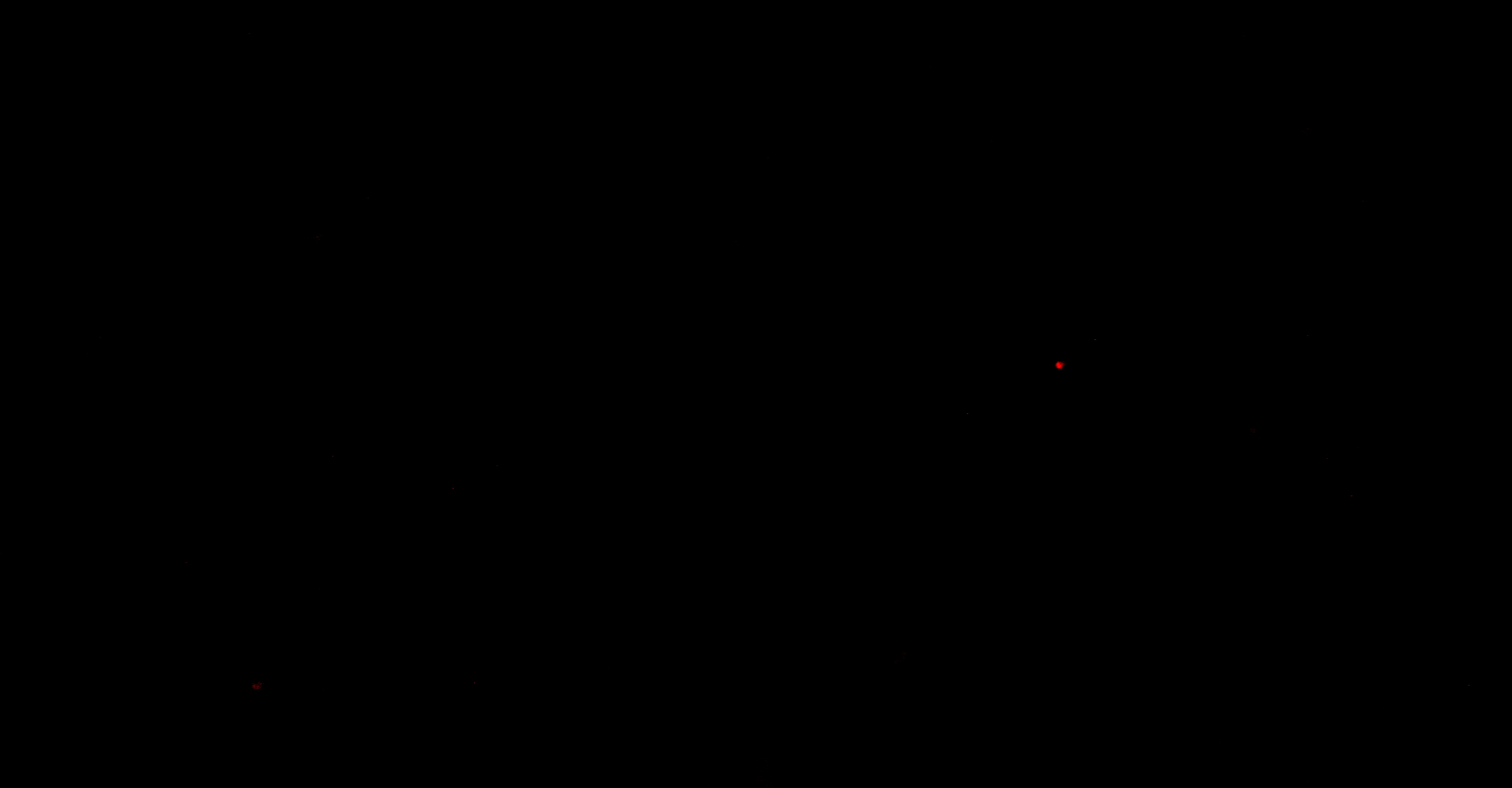

Supplement: S3 File — (ZIP) [file pone.0301540.s003.zip › S3 raw date 3/Fluorescence in situ hybridisation/zutu/tgf-smad3/cont/AN 红 2.jpg]

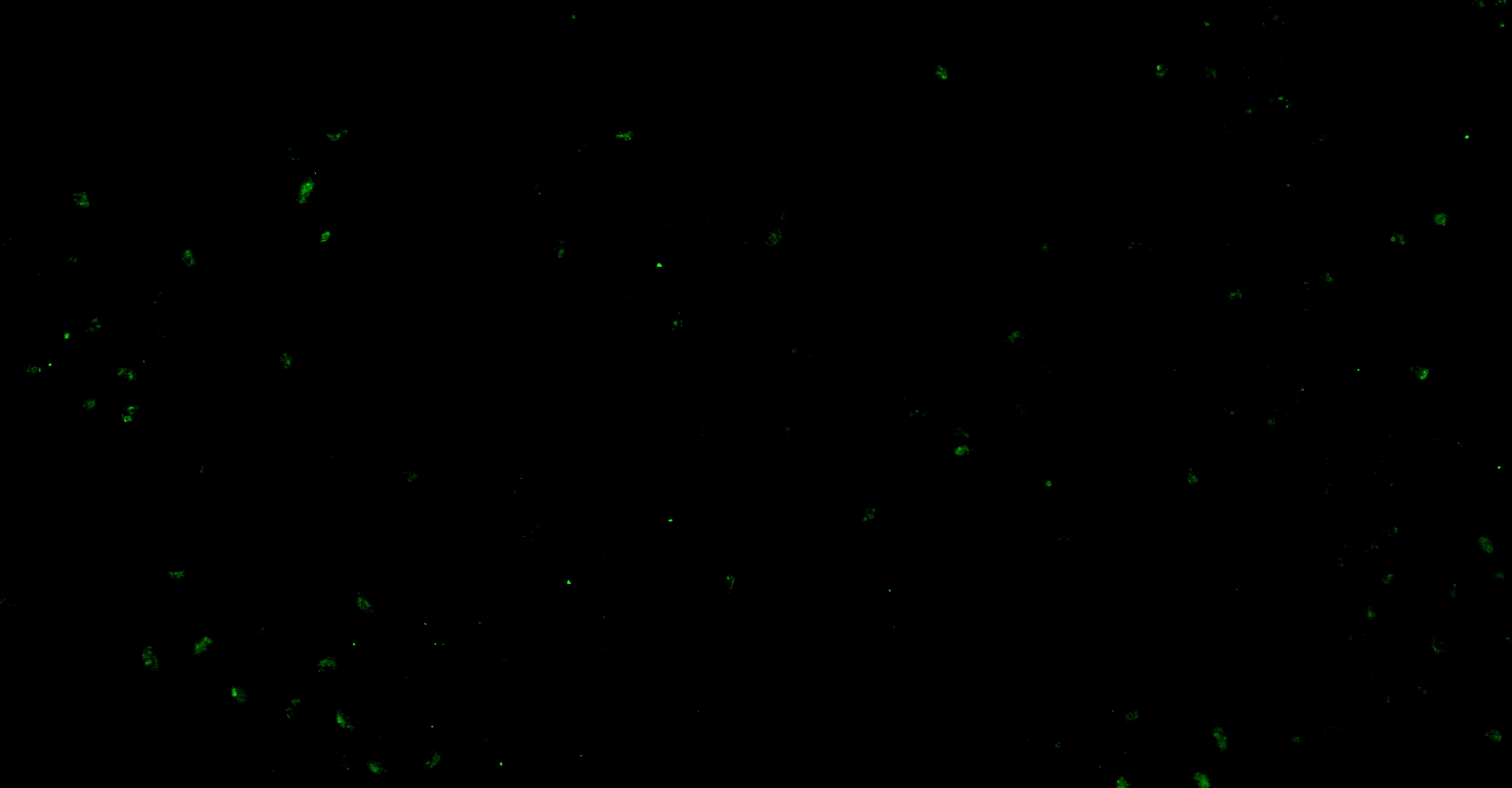

Supplement: S3 File — (ZIP) [file pone.0301540.s003.zip › S3 raw date 3/Fluorescence in situ hybridisation/zutu/tgf-smad3/cont/AN 绿 2.jpg]

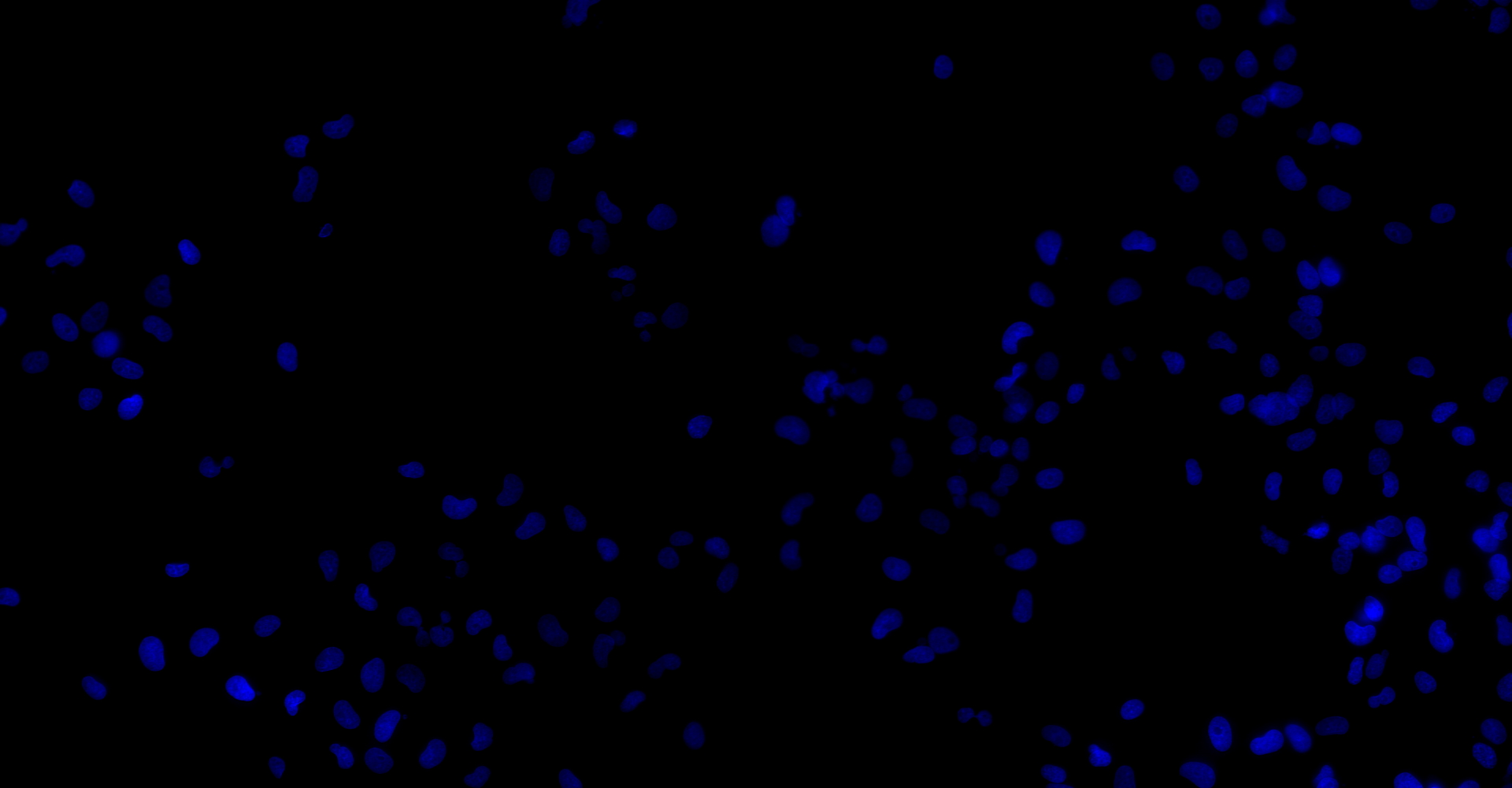

Supplement: S3 File — (ZIP) [file pone.0301540.s003.zip › S3 raw date 3/Fluorescence in situ hybridisation/zutu/tgf-smad3/cont/AN 蓝 2.jpg]

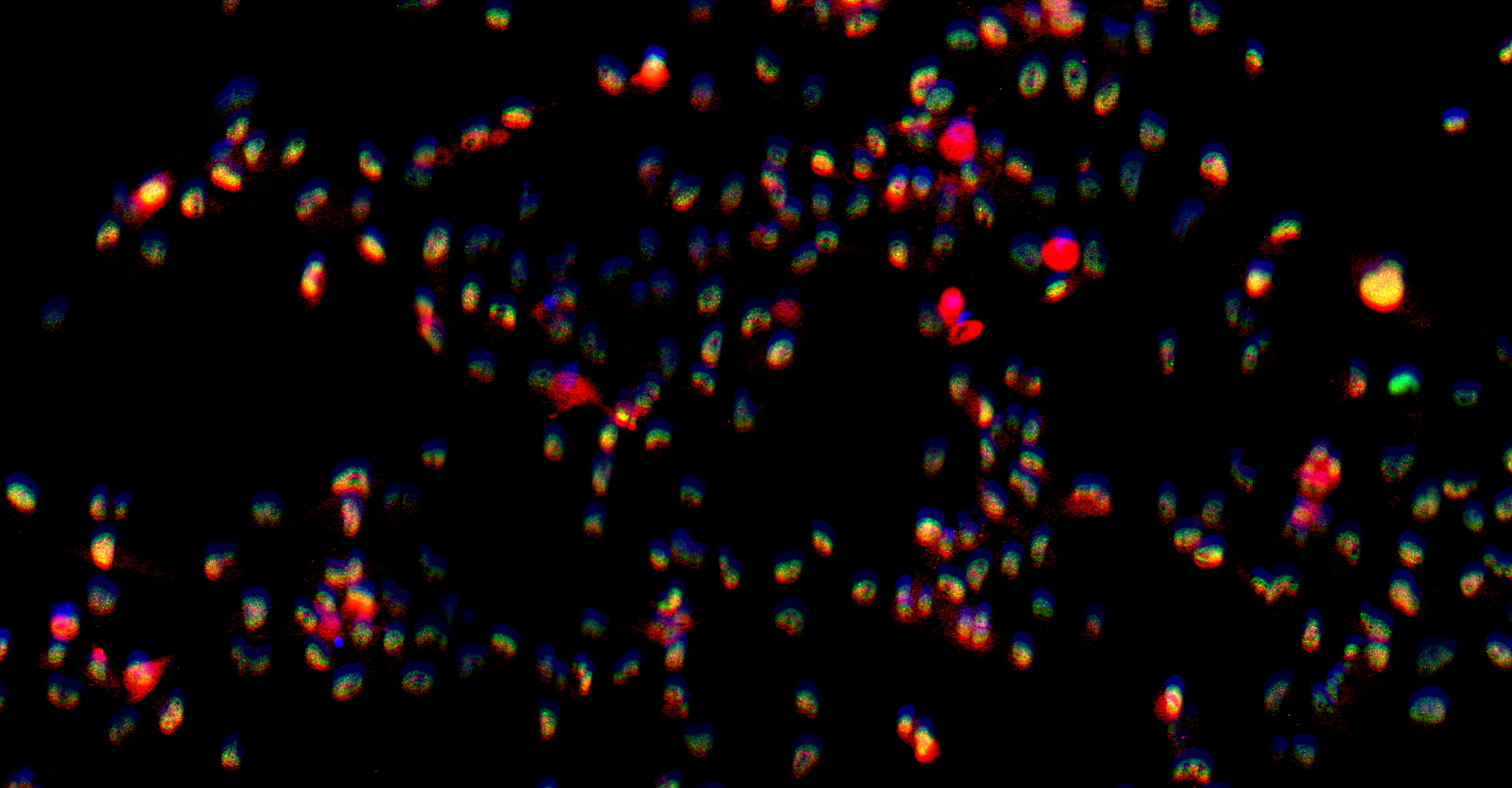

Supplement: S3 File — (ZIP) [file pone.0301540.s003.zip › S3 raw date 3/Fluorescence in situ hybridisation/zutu/tgf-smad3/in/B3D 全 3.jpg]

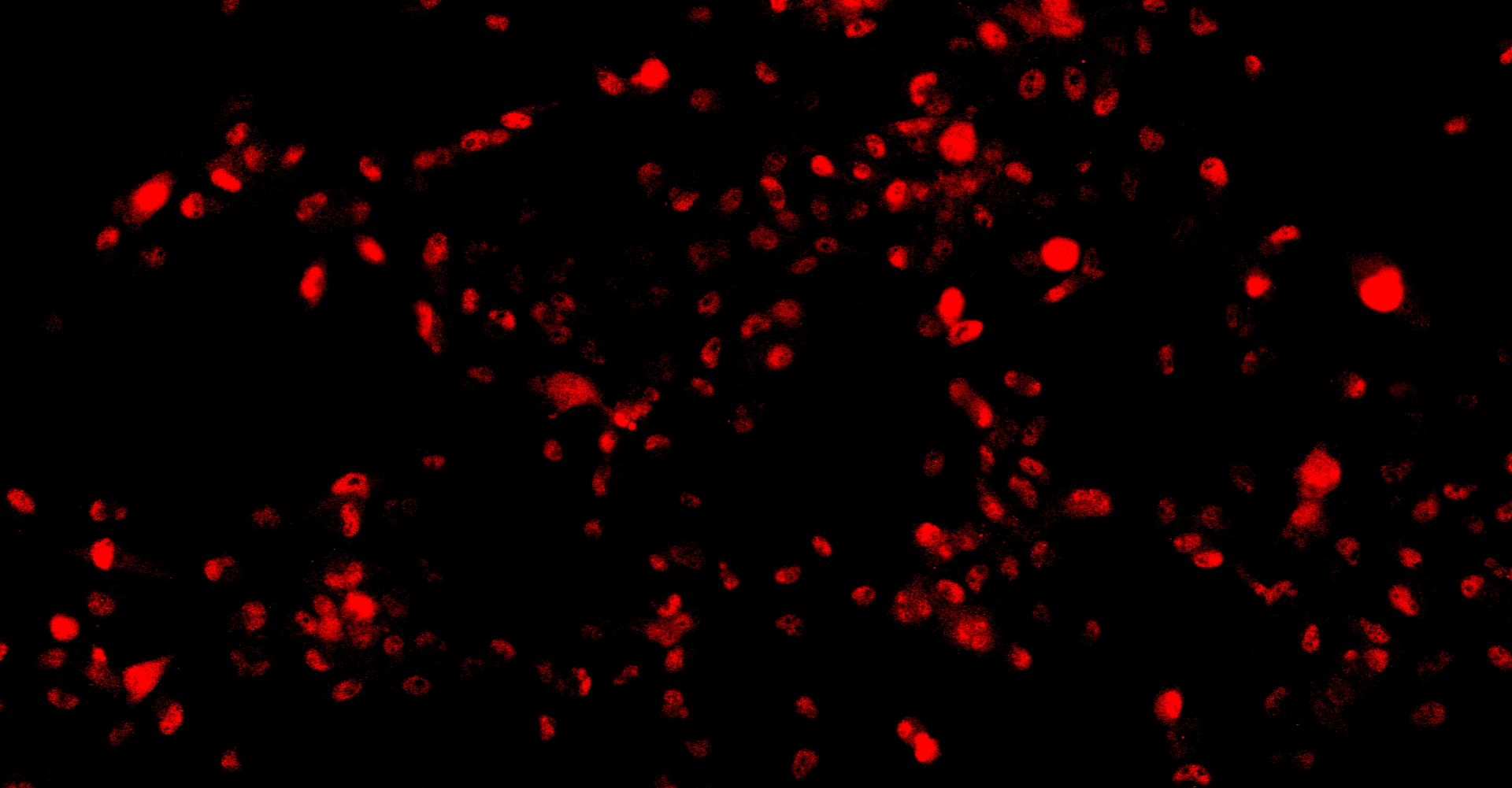

Supplement: S3 File — (ZIP) [file pone.0301540.s003.zip › S3 raw date 3/Fluorescence in situ hybridisation/zutu/tgf-smad3/in/B3D 红 3.jpg]

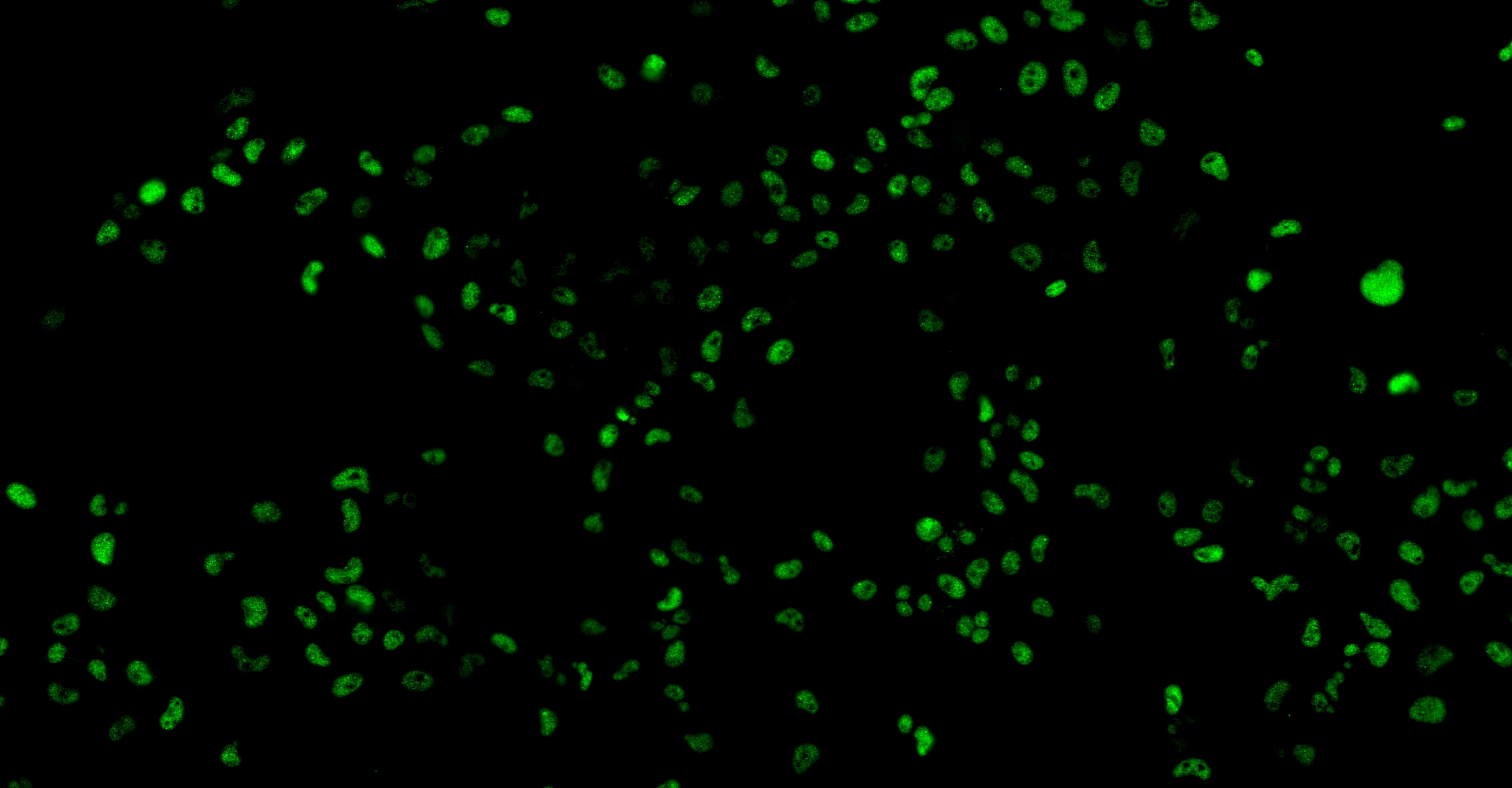

Supplement: S3 File — (ZIP) [file pone.0301540.s003.zip › S3 raw date 3/Fluorescence in situ hybridisation/zutu/tgf-smad3/in/B3D 绿 3.jpg]

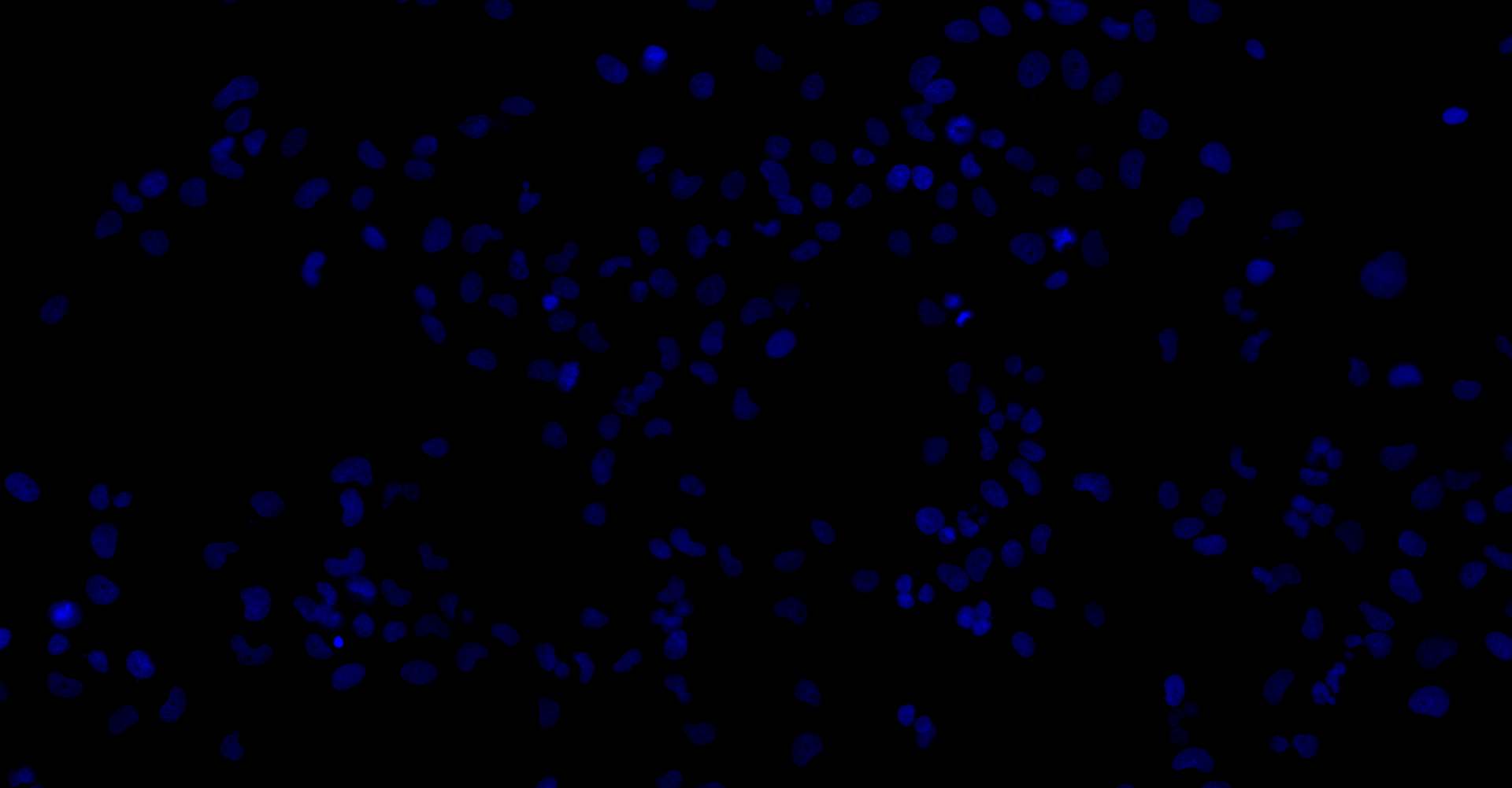

Supplement: S3 File — (ZIP) [file pone.0301540.s003.zip › S3 raw date 3/Fluorescence in situ hybridisation/zutu/tgf-smad3/in/B3D 蓝 3.jpg]

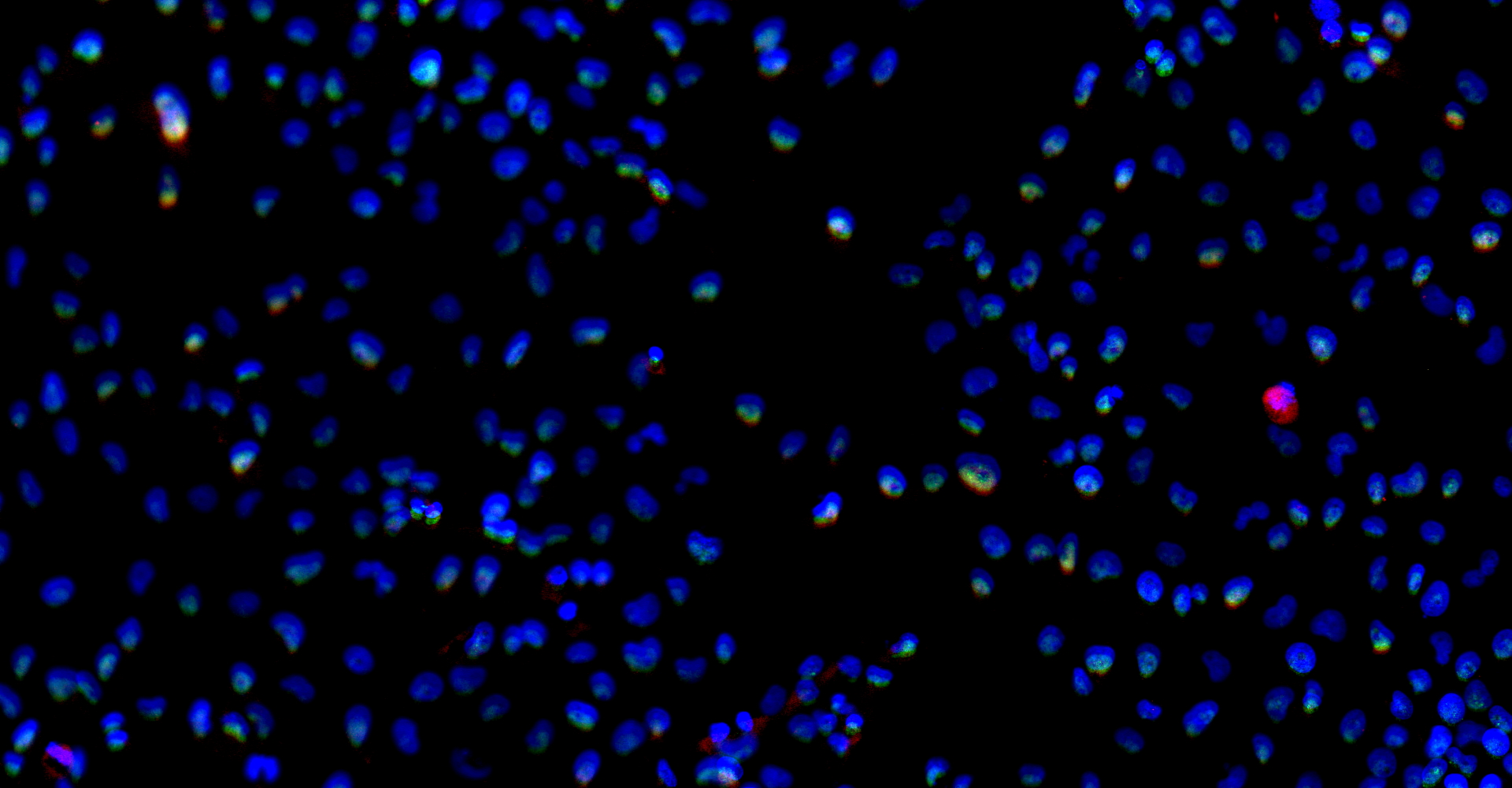

Supplement: S3 File — (ZIP) [file pone.0301540.s003.zip › S3 raw date 3/Fluorescence in situ hybridisation/zutu/tgf-smad3/mi/B1D 全 2.jpg]

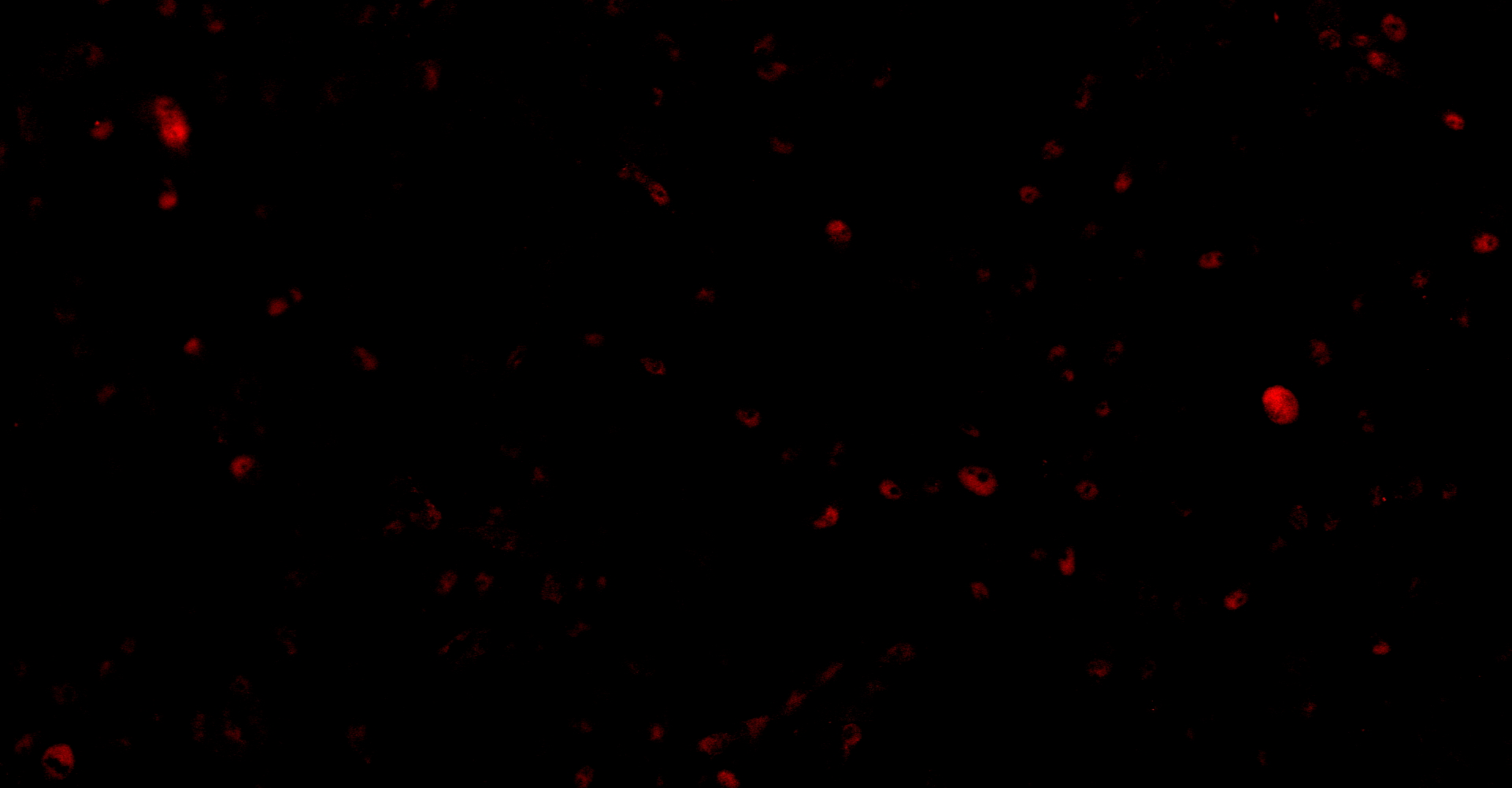

Supplement: S3 File — (ZIP) [file pone.0301540.s003.zip › S3 raw date 3/Fluorescence in situ hybridisation/zutu/tgf-smad3/mi/B1D 红 2.jpg]

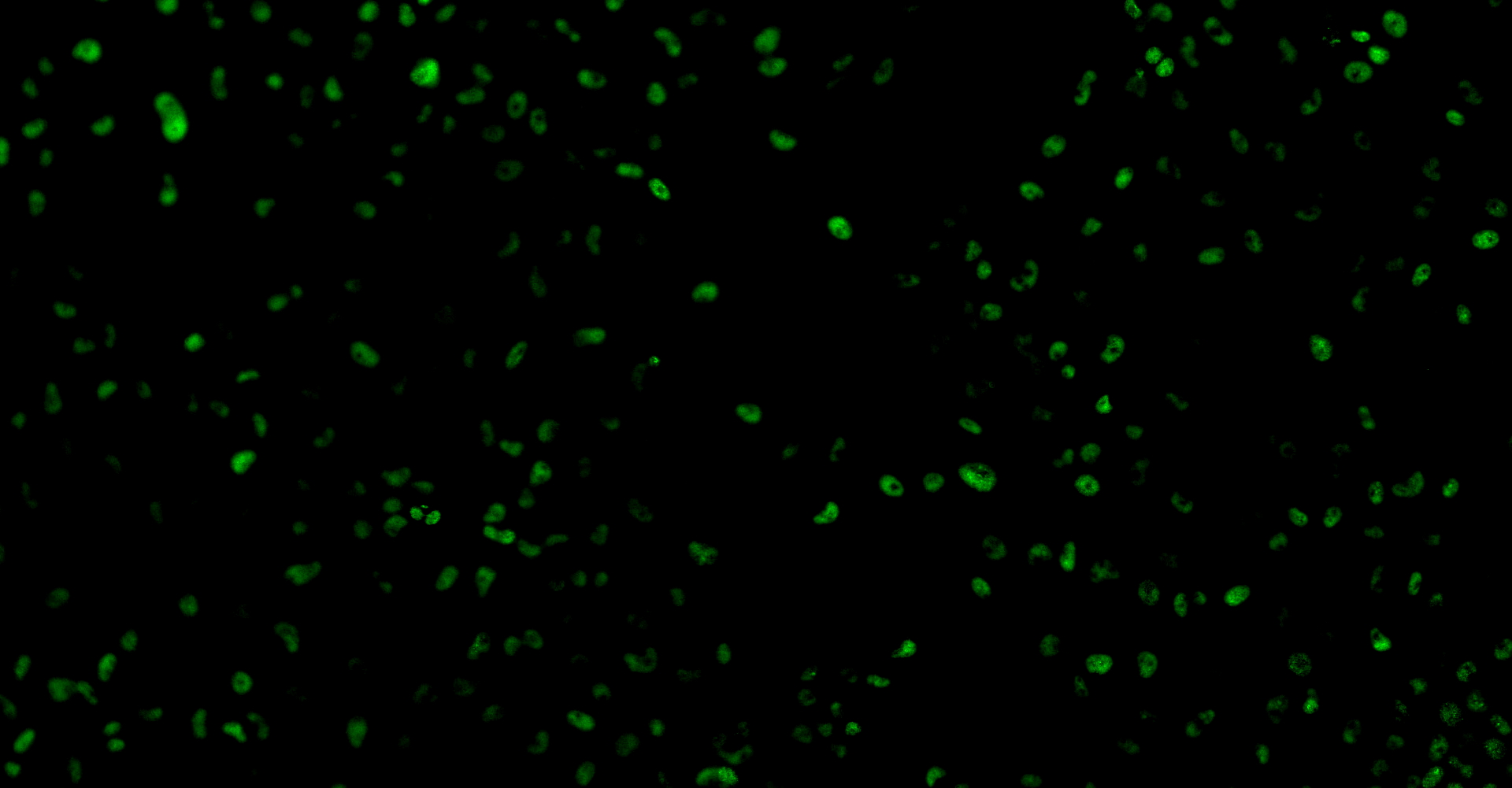

Supplement: S3 File — (ZIP) [file pone.0301540.s003.zip › S3 raw date 3/Fluorescence in situ hybridisation/zutu/tgf-smad3/mi/B1D 绿 2.jpg]

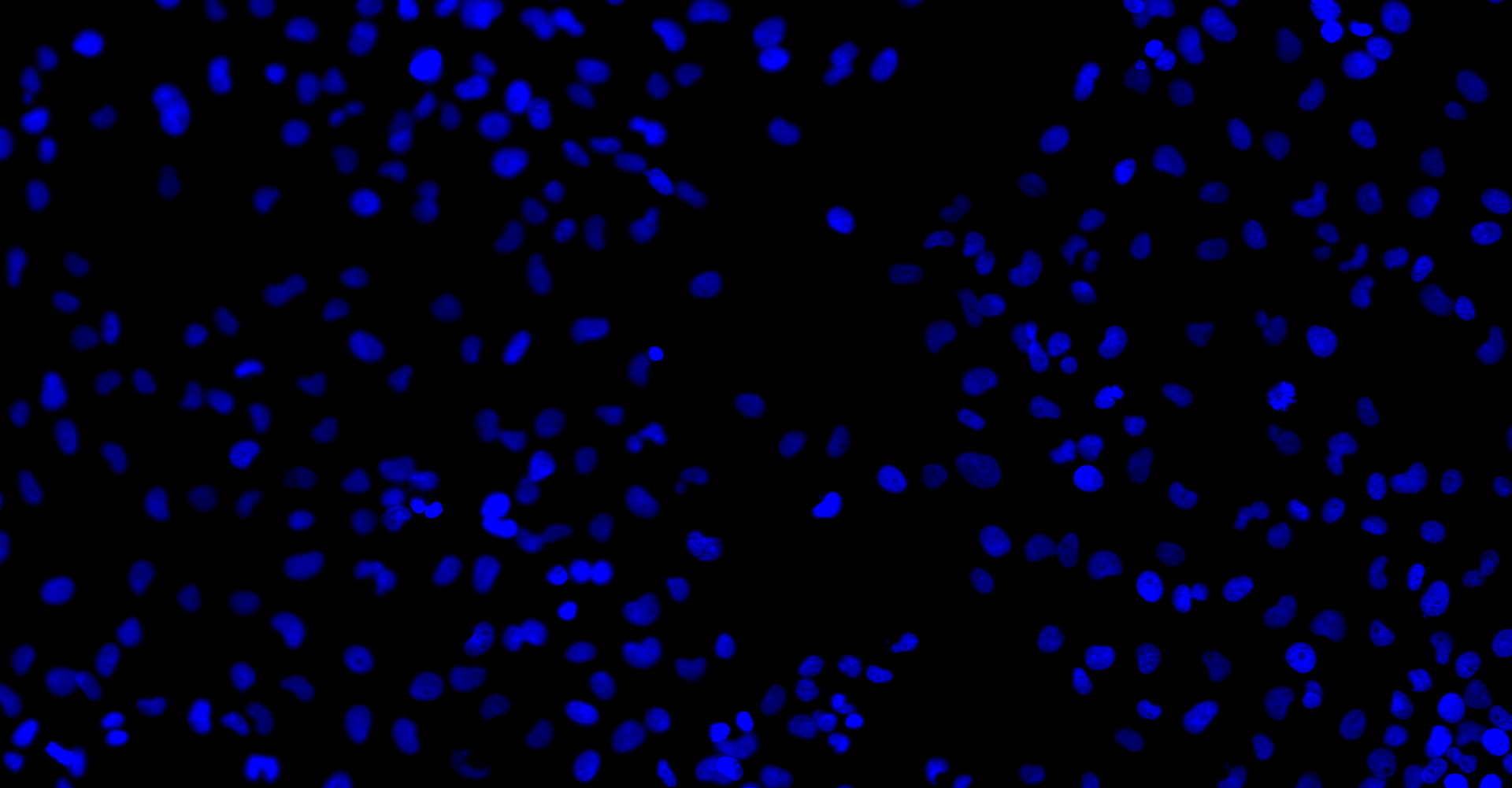

Supplement: S3 File — (ZIP) [file pone.0301540.s003.zip › S3 raw date 3/Fluorescence in situ hybridisation/zutu/tgf-smad3/mi/B1D 蓝 2.jpg]

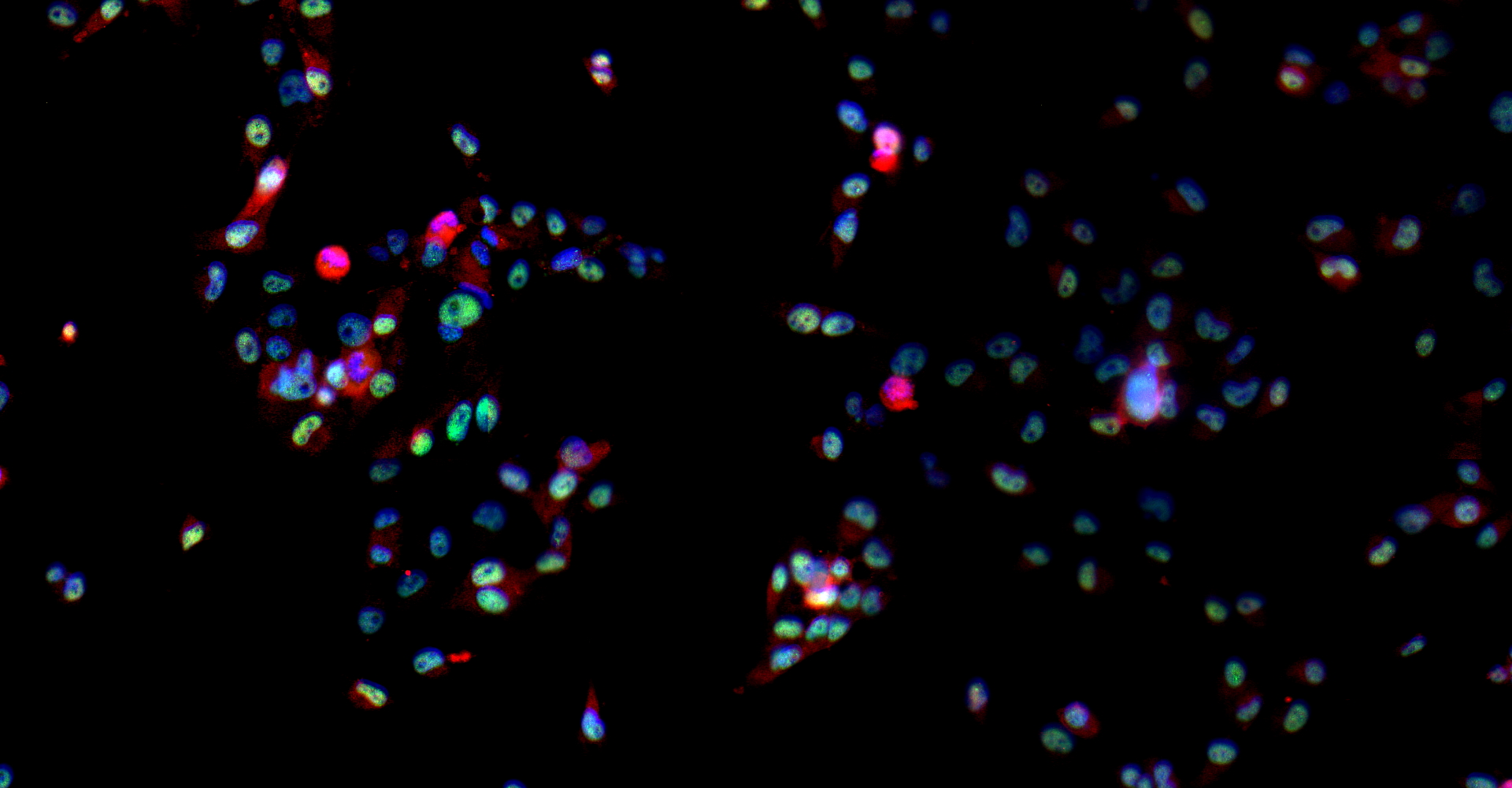

Supplement: S3 File — (ZIP) [file pone.0301540.s003.zip › S3 raw date 3/Fluorescence in situ hybridisation/zutu/tgf-smad3/tgf/B2M 全 3.jpg]

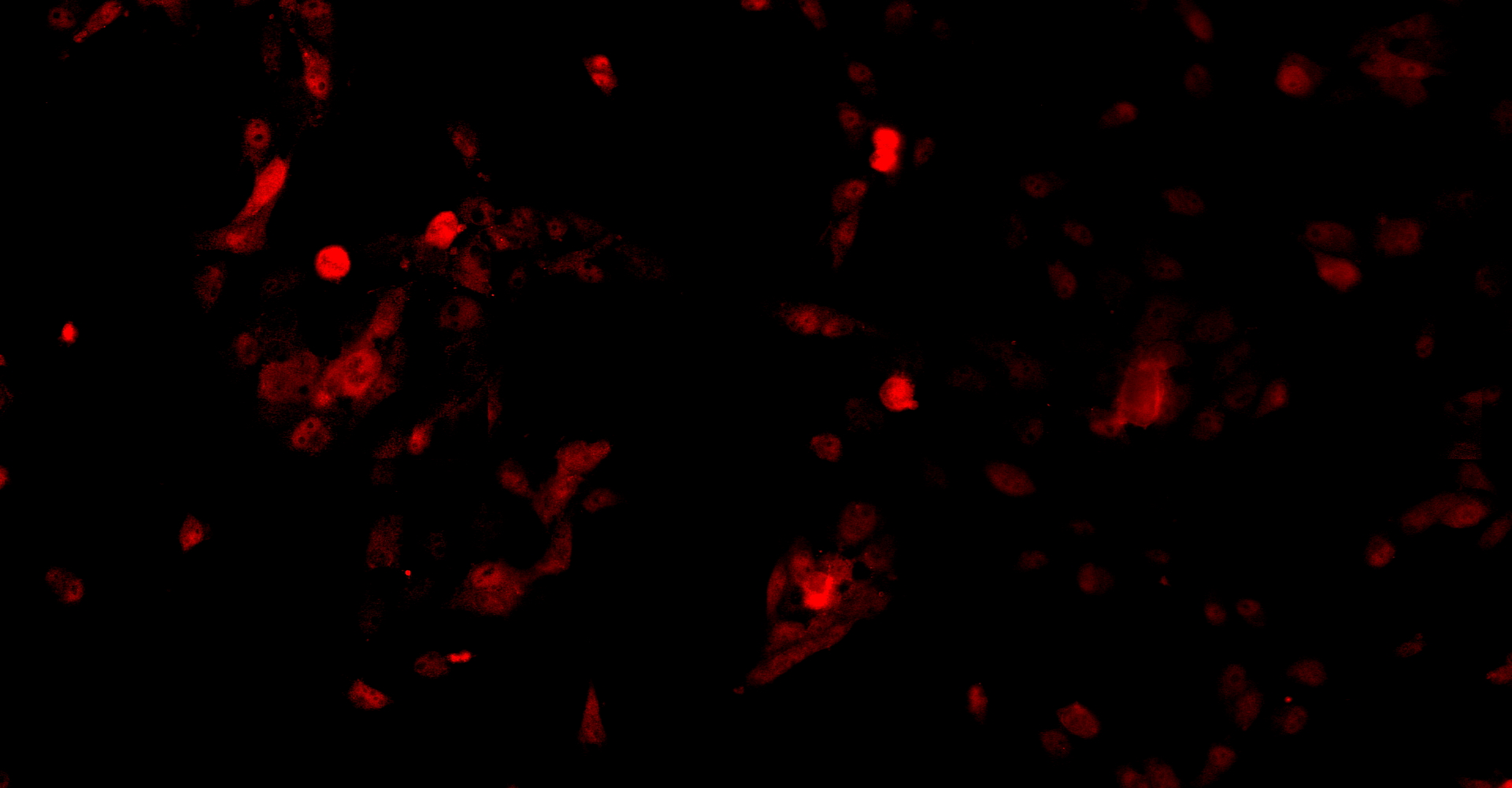

Supplement: S3 File — (ZIP) [file pone.0301540.s003.zip › S3 raw date 3/Fluorescence in situ hybridisation/zutu/tgf-smad3/tgf/B2M 红 3.jpg]

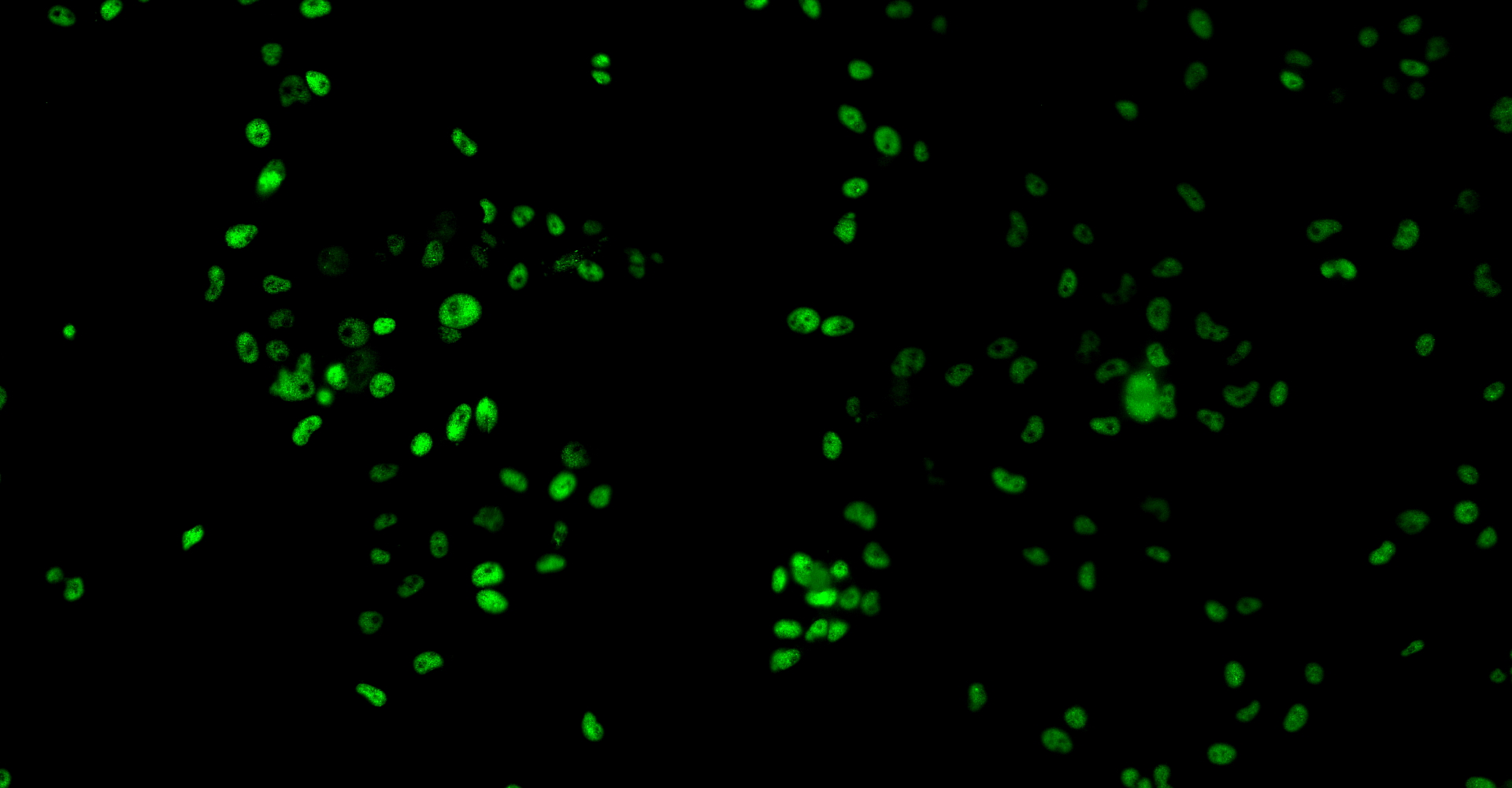

Supplement: S3 File — (ZIP) [file pone.0301540.s003.zip › S3 raw date 3/Fluorescence in situ hybridisation/zutu/tgf-smad3/tgf/B2M 绿 3.jpg]

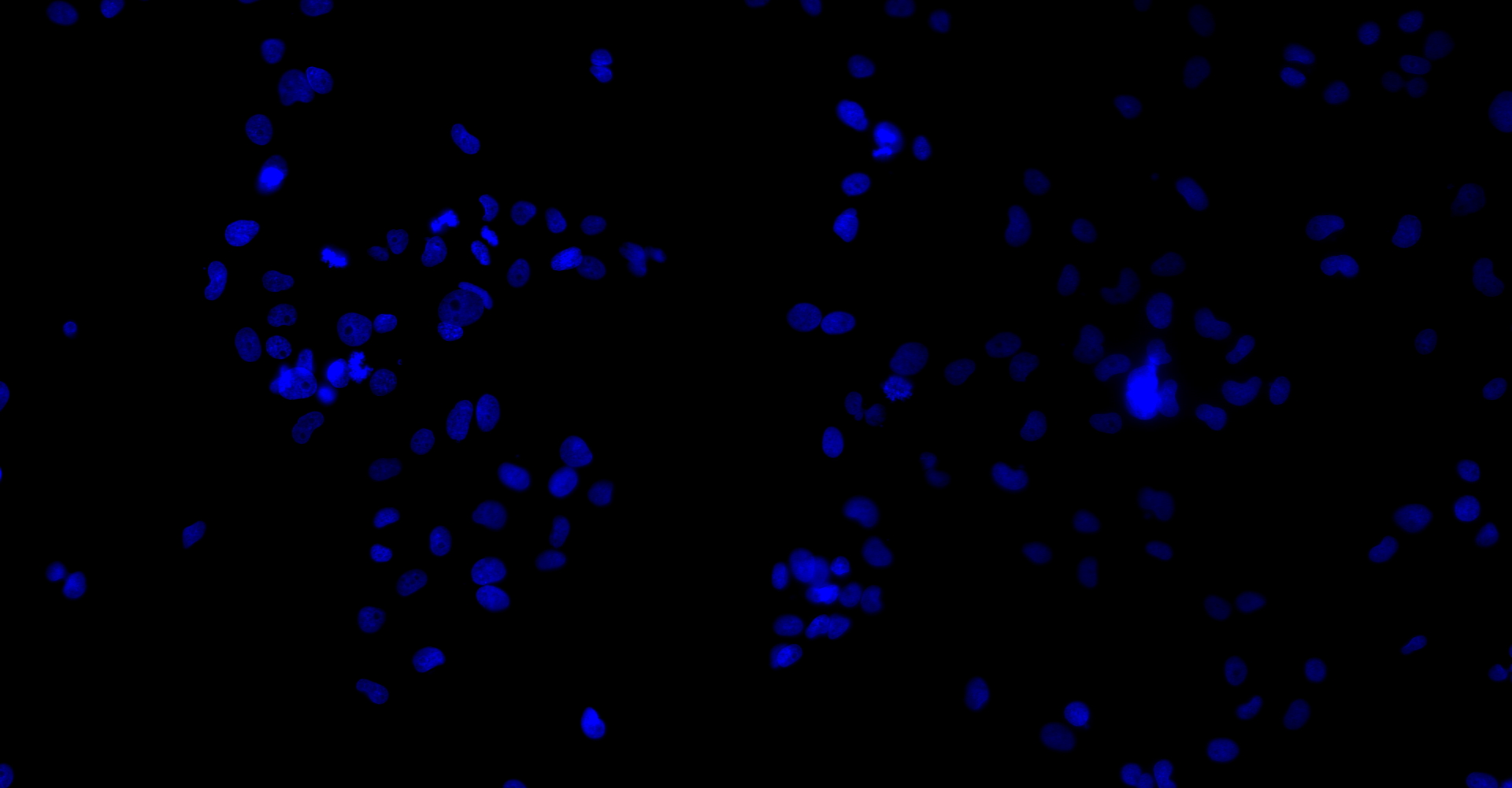

Supplement: S3 File — (ZIP) [file pone.0301540.s003.zip › S3 raw date 3/Fluorescence in situ hybridisation/zutu/tgf-smad3/tgf/B2M 蓝 3.jpg]

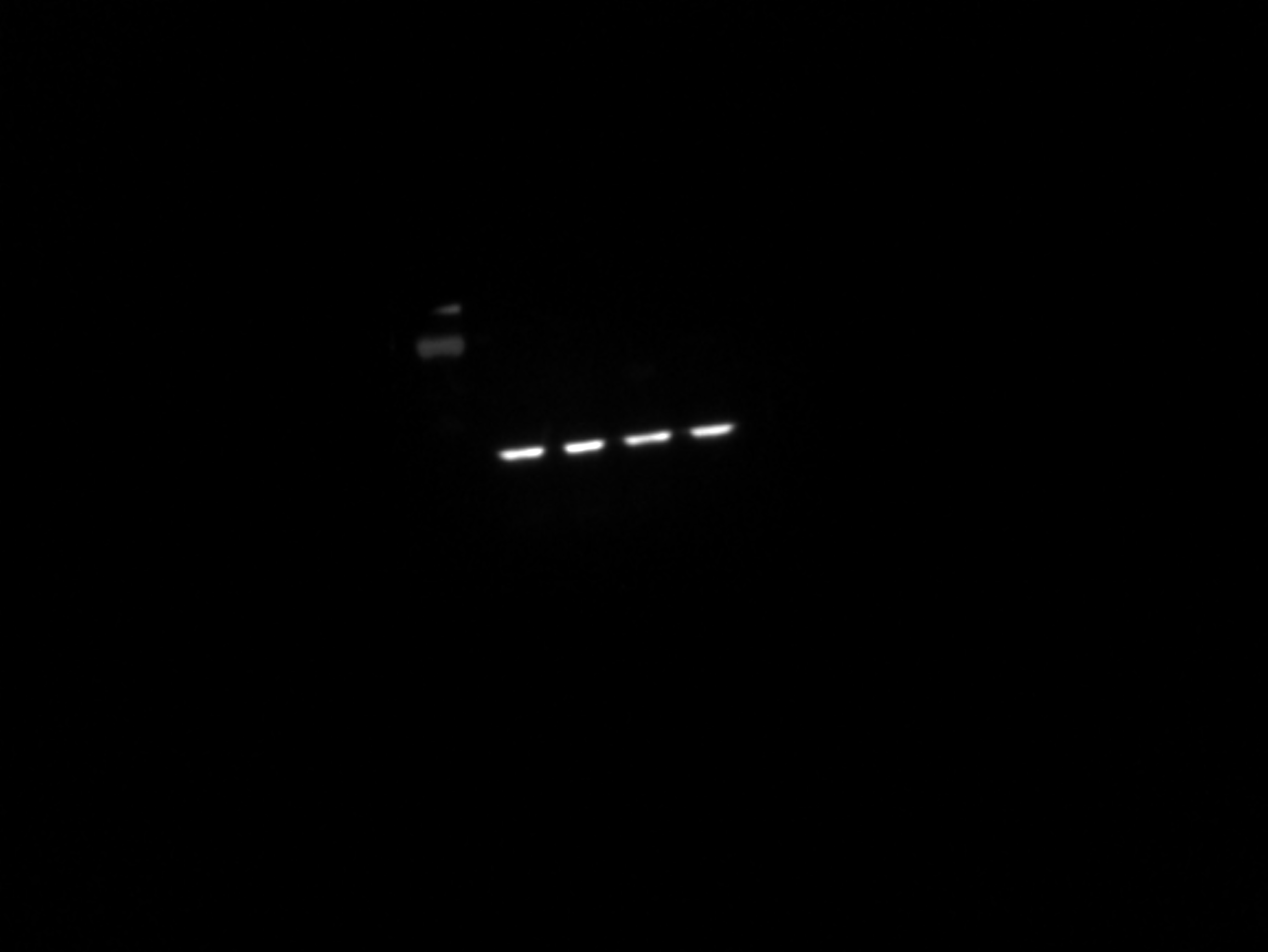

Supplement: S4 File — (ZIP) [file pone.0301540.s004.zip › S4 raw date 4/WB/TGF/pictur/1/NEICAN/2024-01-12-135636-image3-sub0-As-Displayed.tif]

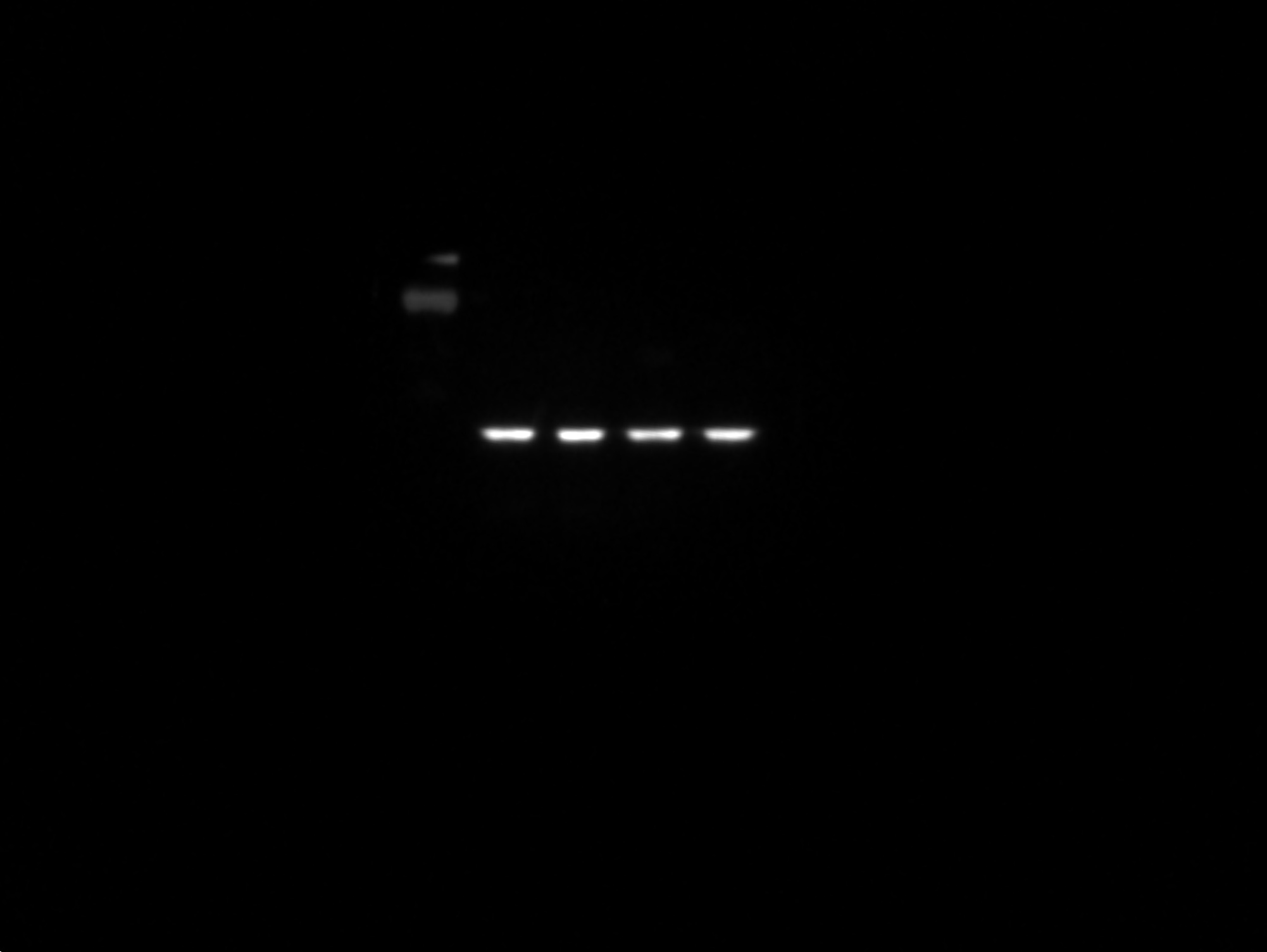

Supplement: S4 File — (ZIP) [file pone.0301540.s004.zip › S4 raw date 4/WB/TGF/pictur/1/NEICAN/2024-01-12-135636-image3-sub0-As-Displayed_副本.jpg]

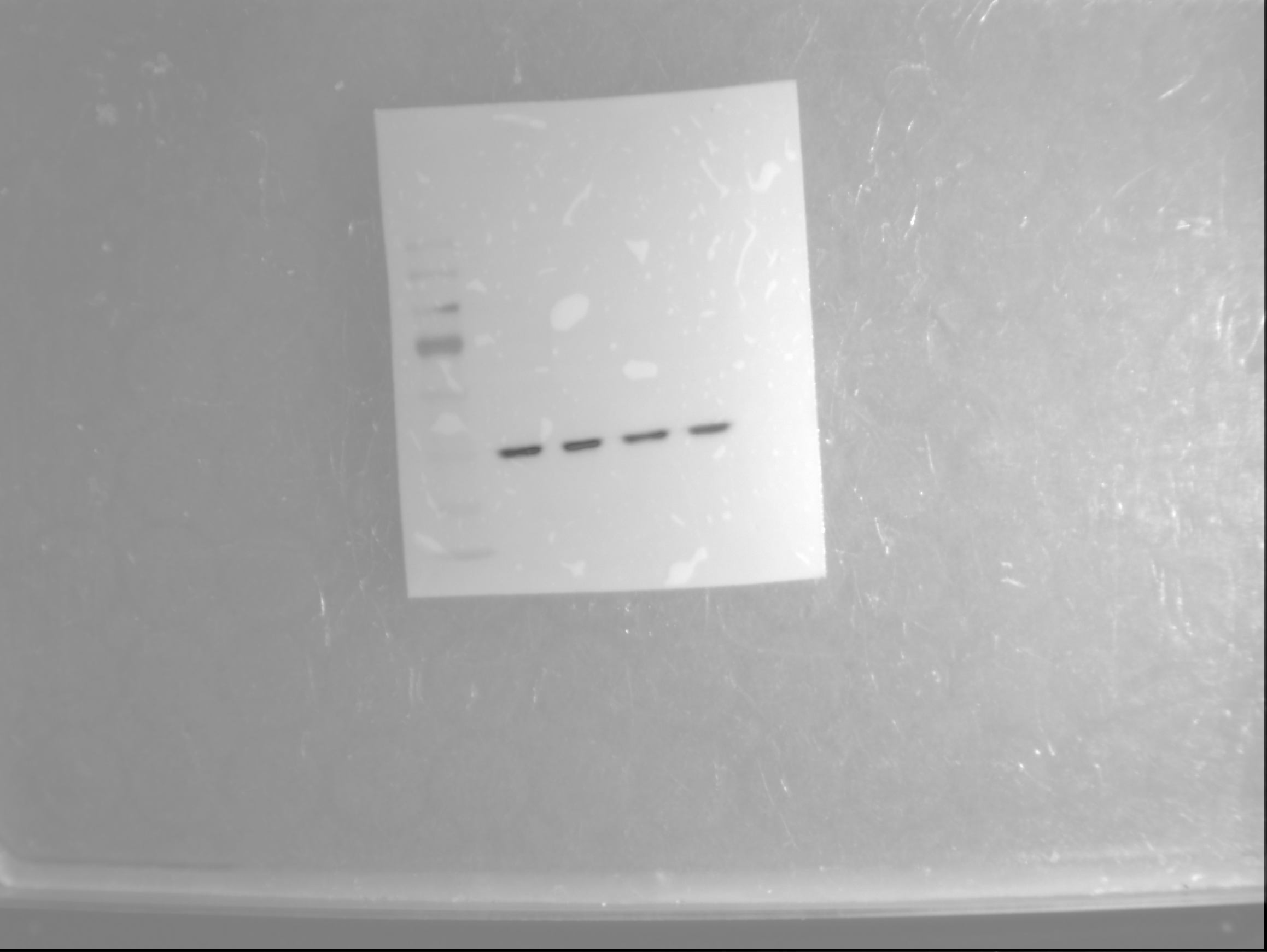

Supplement: S4 File — (ZIP) [file pone.0301540.s004.zip › S4 raw date 4/WB/TGF/pictur/1/NEICAN/2024-01-12-135636-image3.tif]

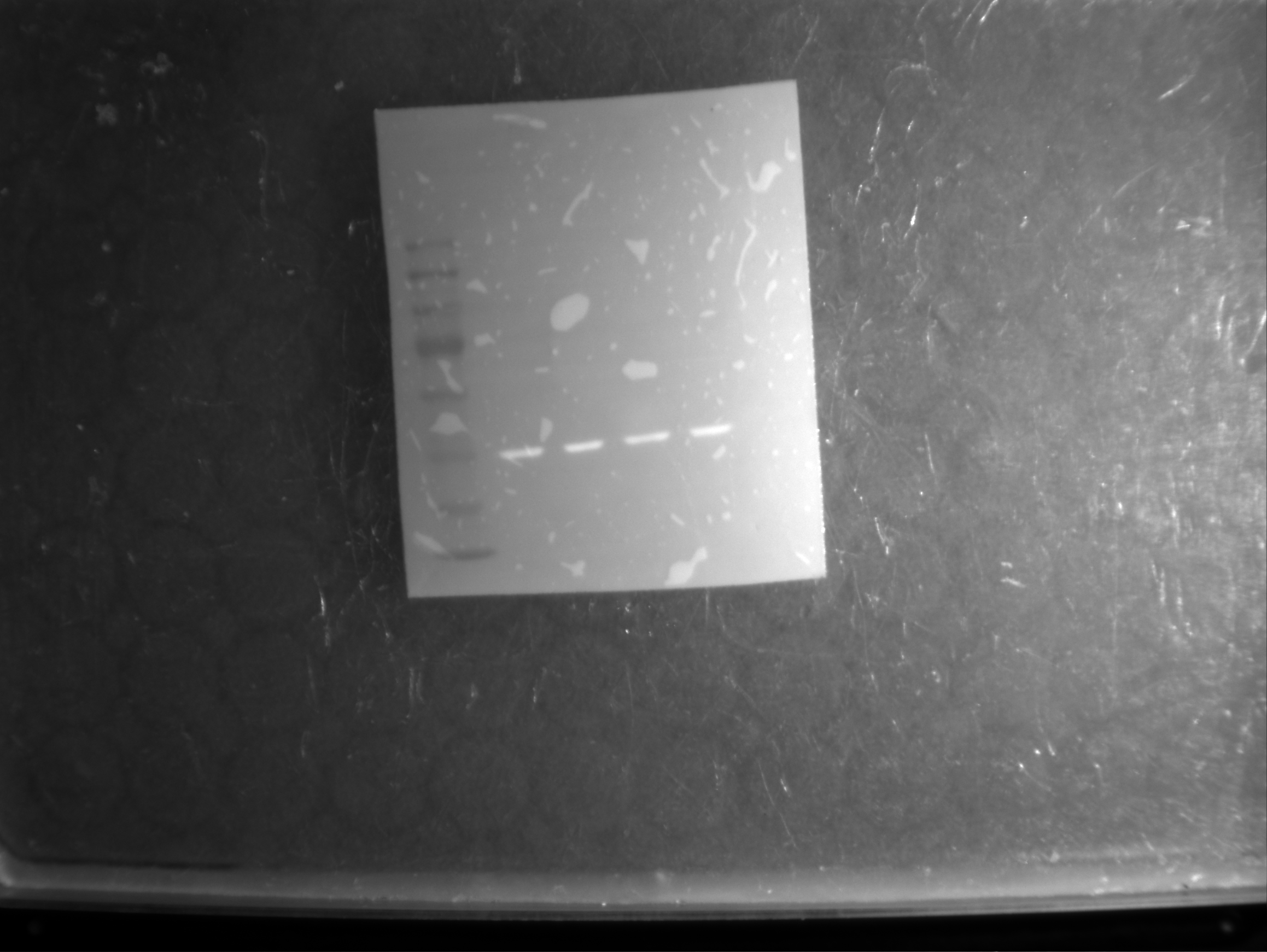

Supplement: S4 File — (ZIP) [file pone.0301540.s004.zip › S4 raw date 4/WB/TGF/pictur/1/NEICAN/2024-01-12-135640-image3-sub1-As-Displayed.tif]

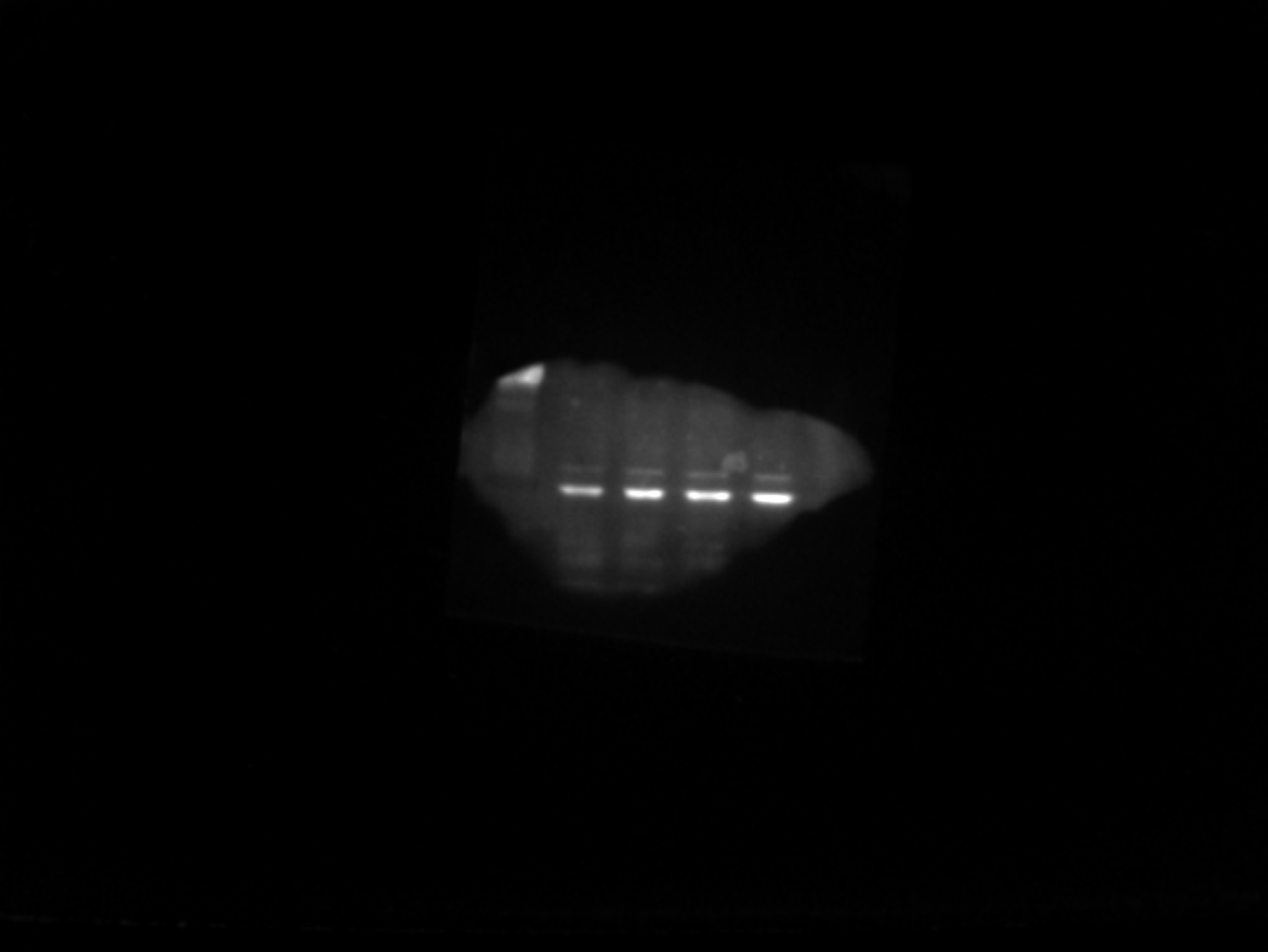

Supplement: S4 File — (ZIP) [file pone.0301540.s004.zip › S4 raw date 4/WB/TGF/pictur/1/tgf/2024-01-13-165043-image1-sub0-As-Displayed.tif]

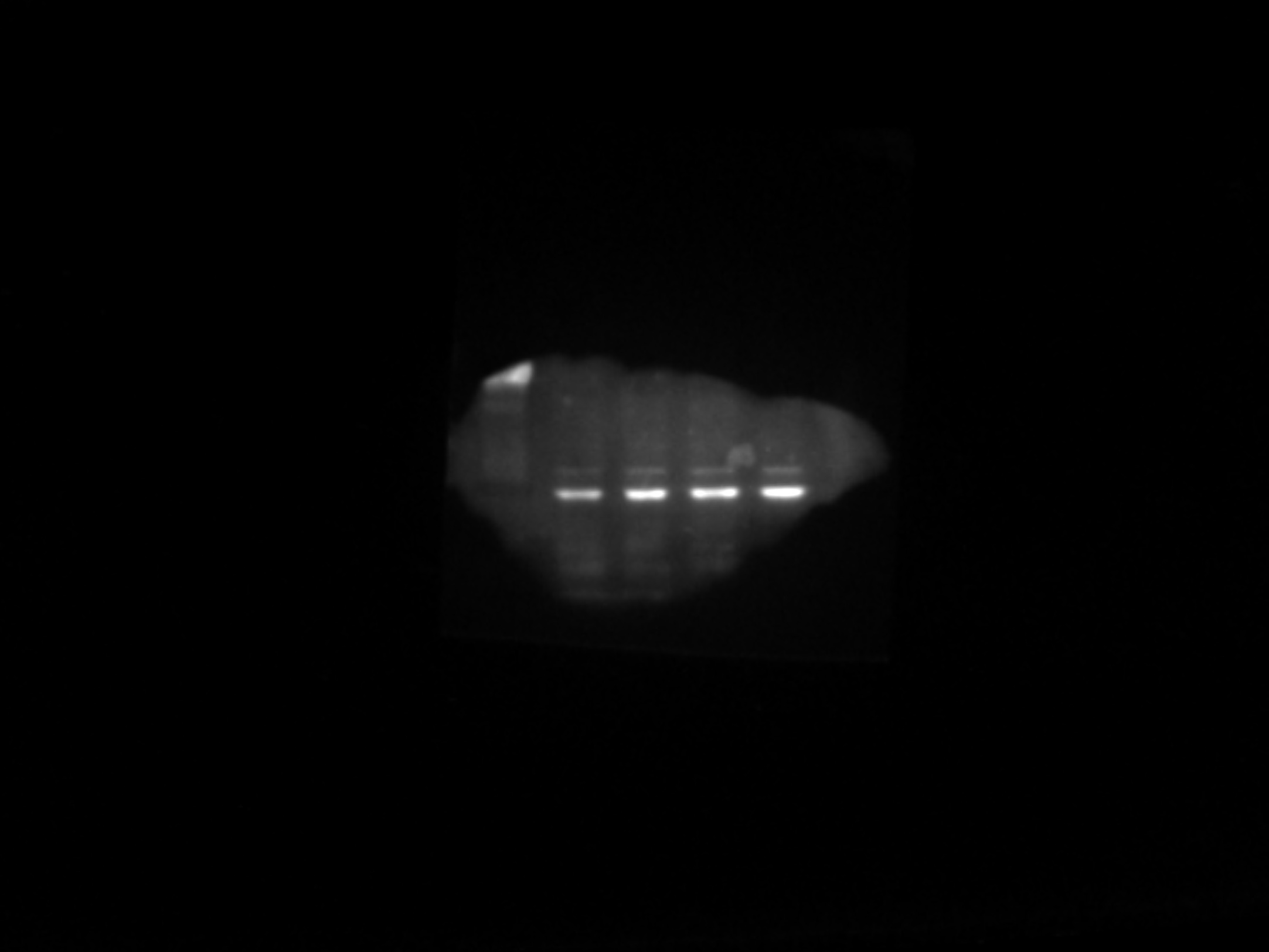

Supplement: S4 File — (ZIP) [file pone.0301540.s004.zip › S4 raw date 4/WB/TGF/pictur/1/tgf/2024-01-13-165043-image1-sub0-As-Displayed_副本.jpg]

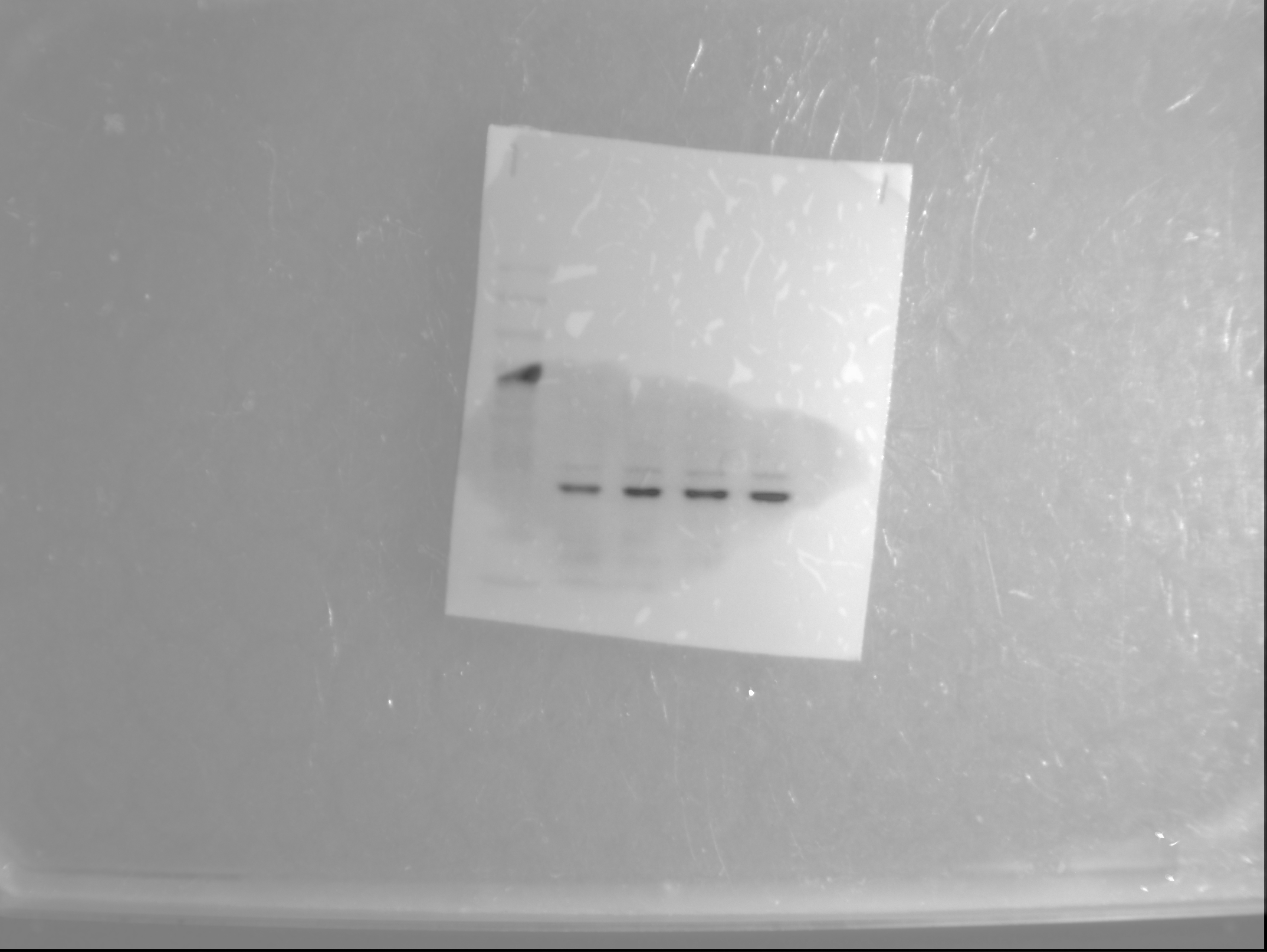

Supplement: S4 File — (ZIP) [file pone.0301540.s004.zip › S4 raw date 4/WB/TGF/pictur/1/tgf/2024-01-13-165043-image1.tif]

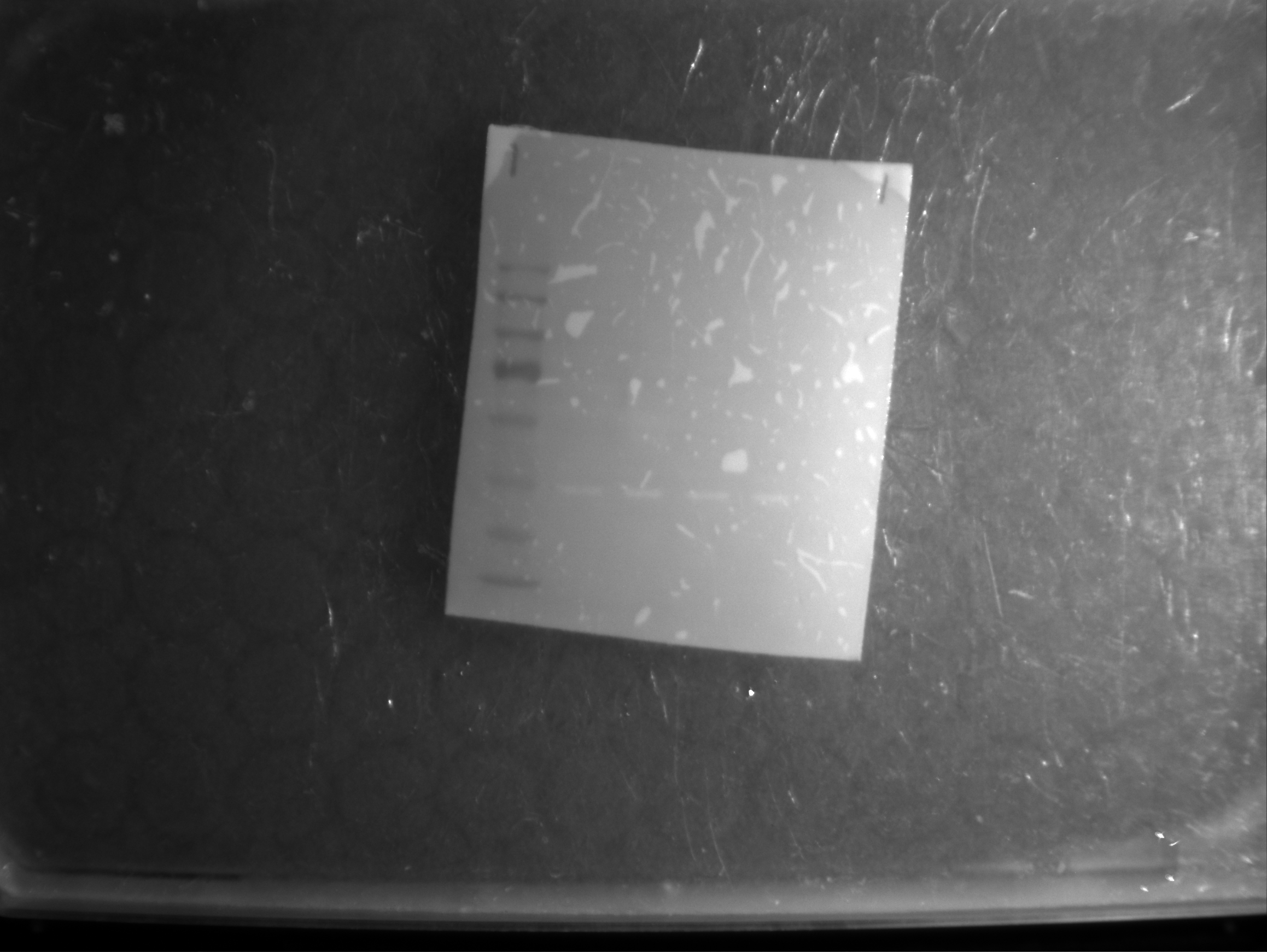

Supplement: S4 File — (ZIP) [file pone.0301540.s004.zip › S4 raw date 4/WB/TGF/pictur/1/tgf/2024-01-13-165047-image1-sub1-As-Displayed.tif]

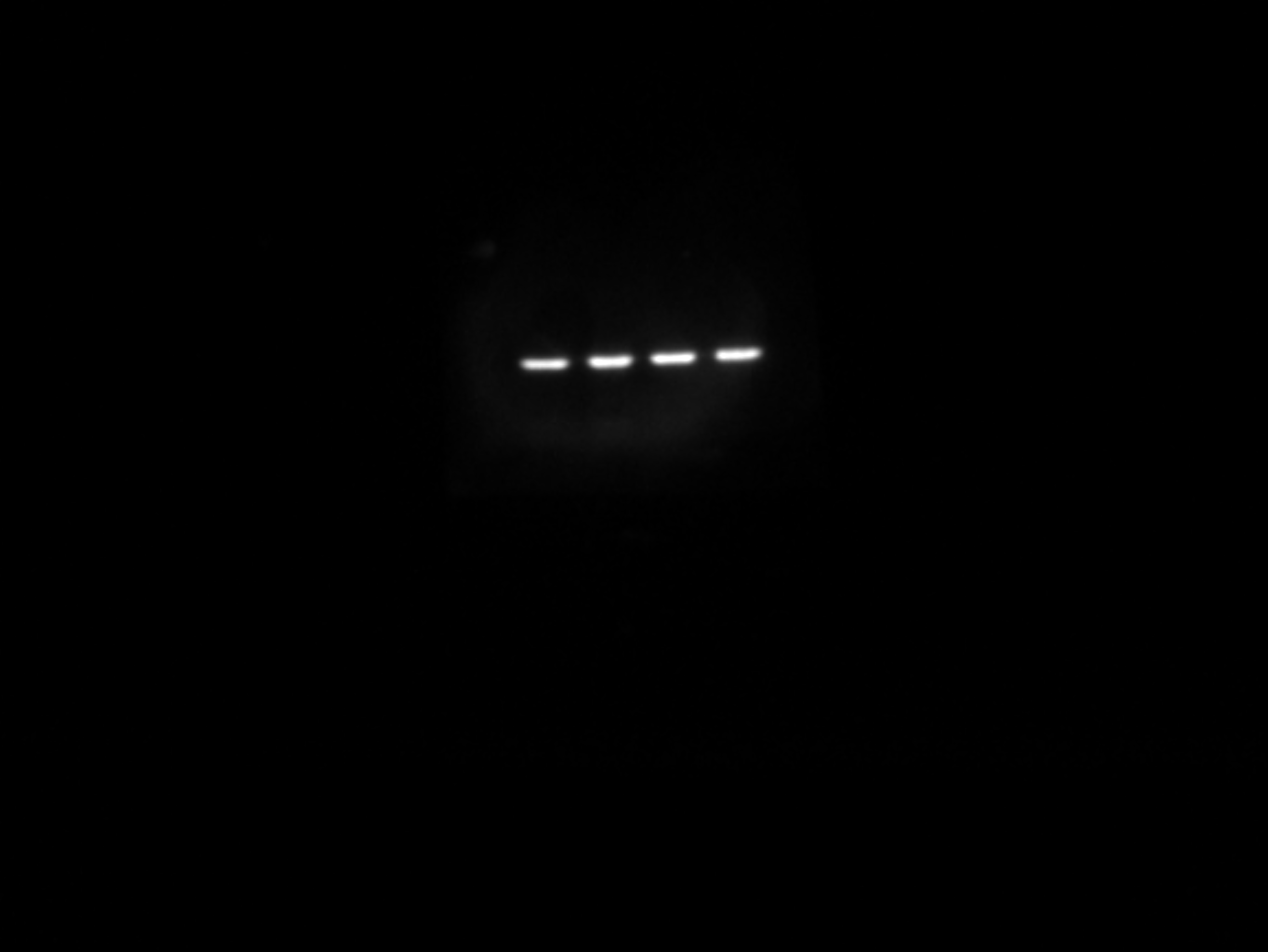

Supplement: S4 File — (ZIP) [file pone.0301540.s004.zip › S4 raw date 4/WB/TGF/pictur/3/neican/2024-01-14-203602-image11-sub0-As-Displayed.tif]

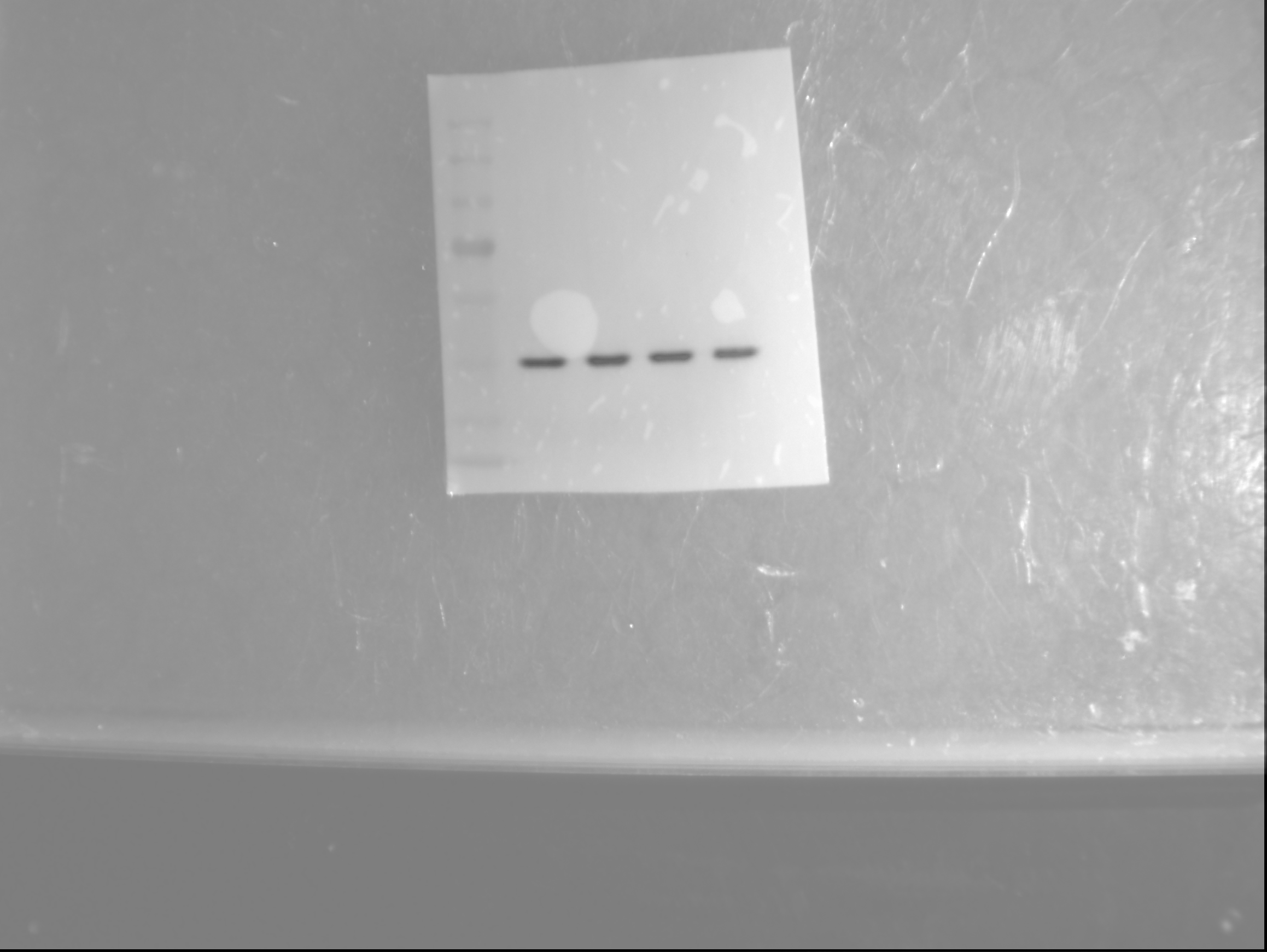

Supplement: S4 File — (ZIP) [file pone.0301540.s004.zip › S4 raw date 4/WB/TGF/pictur/3/neican/2024-01-14-203602-image11.tif]

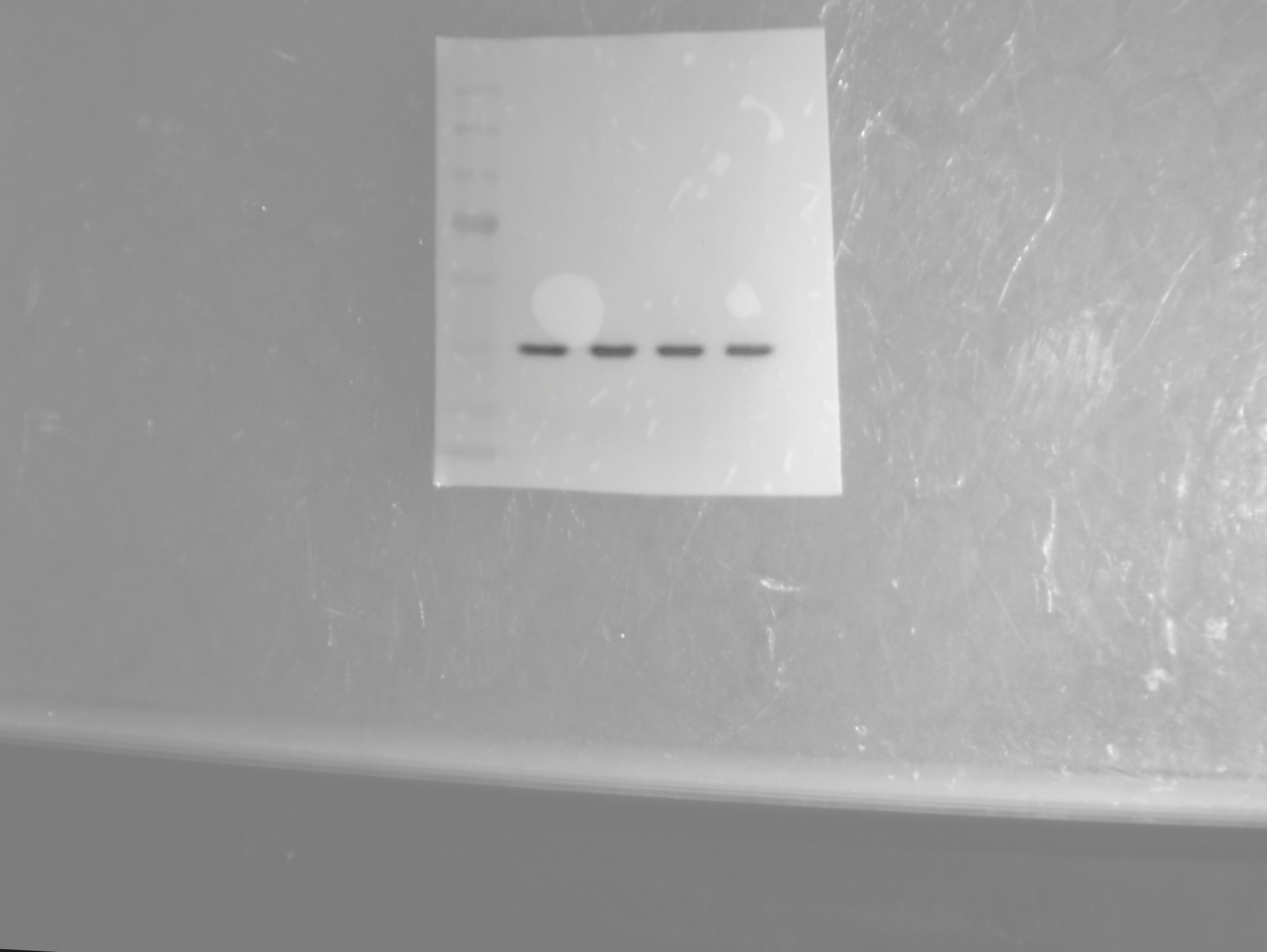

Supplement: S4 File — (ZIP) [file pone.0301540.s004.zip › S4 raw date 4/WB/TGF/pictur/3/neican/2024-01-14-203602-image11_副本.jpg]

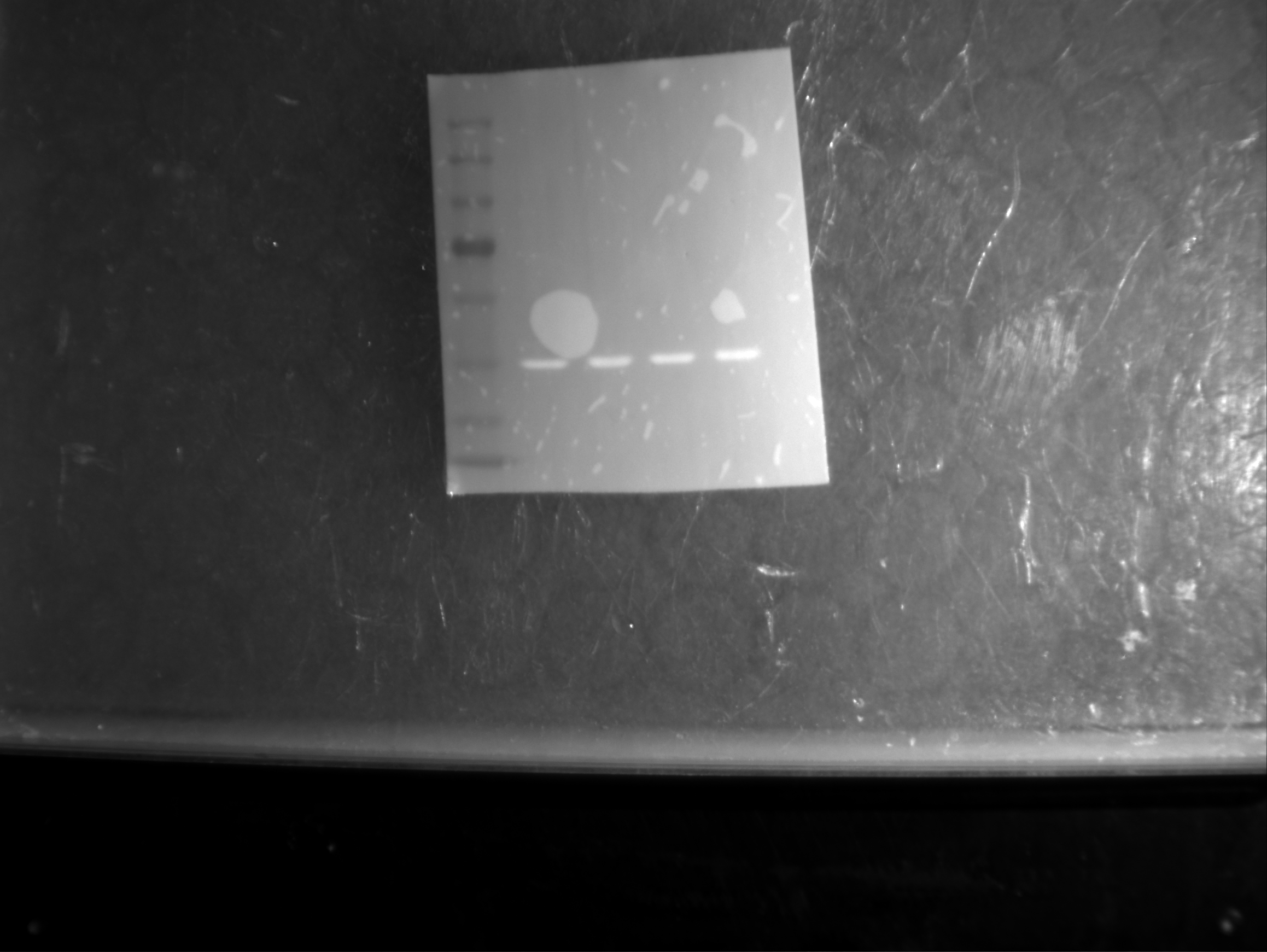

Supplement: S4 File — (ZIP) [file pone.0301540.s004.zip › S4 raw date 4/WB/TGF/pictur/3/neican/2024-01-14-203606-image11-sub1-As-Displayed.tif]

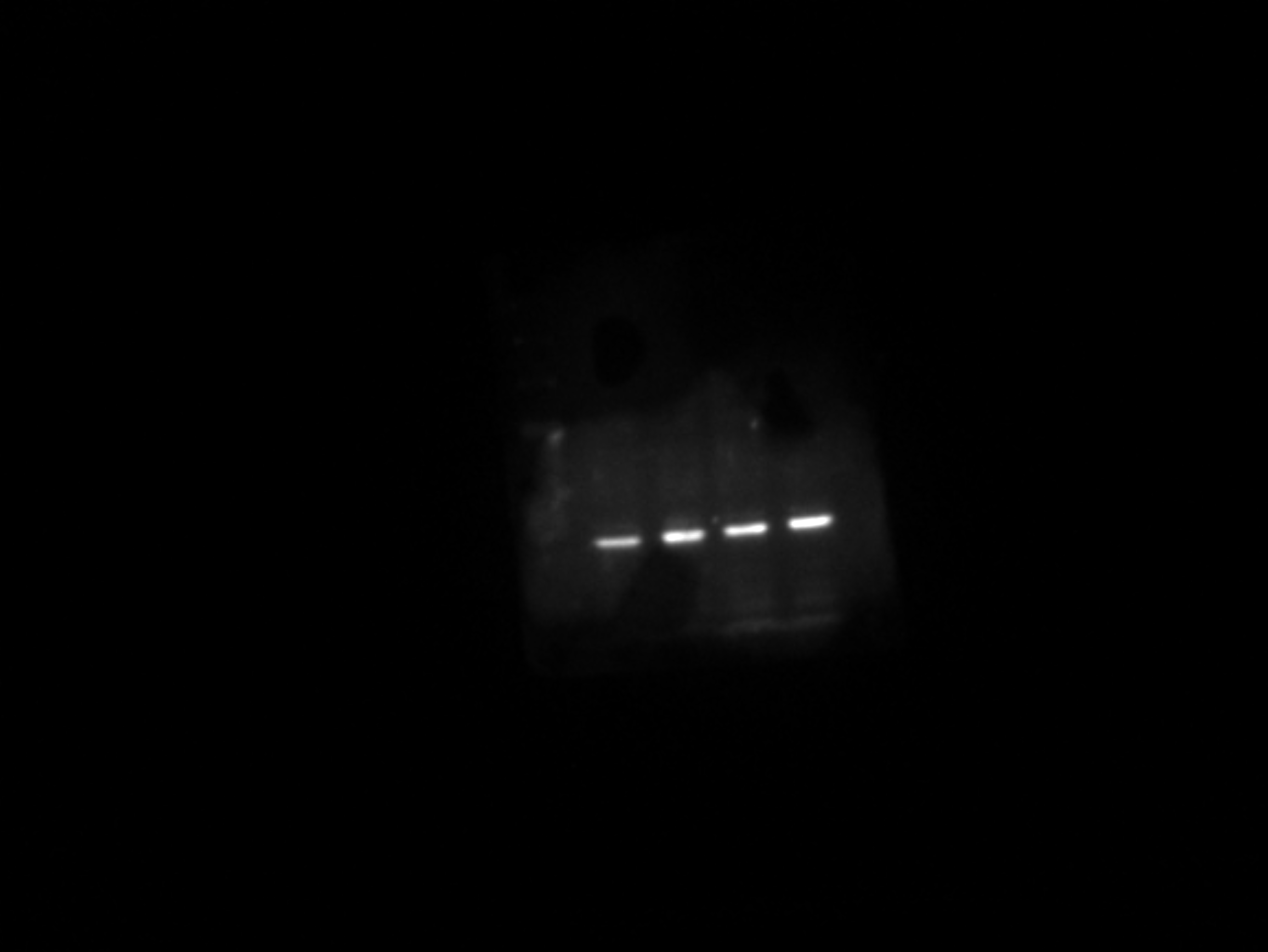

Supplement: S4 File — (ZIP) [file pone.0301540.s004.zip › S4 raw date 4/WB/TGF/pictur/3/tgf/2024-01-15-234232-image5-sub0-As-Displayed.tif]

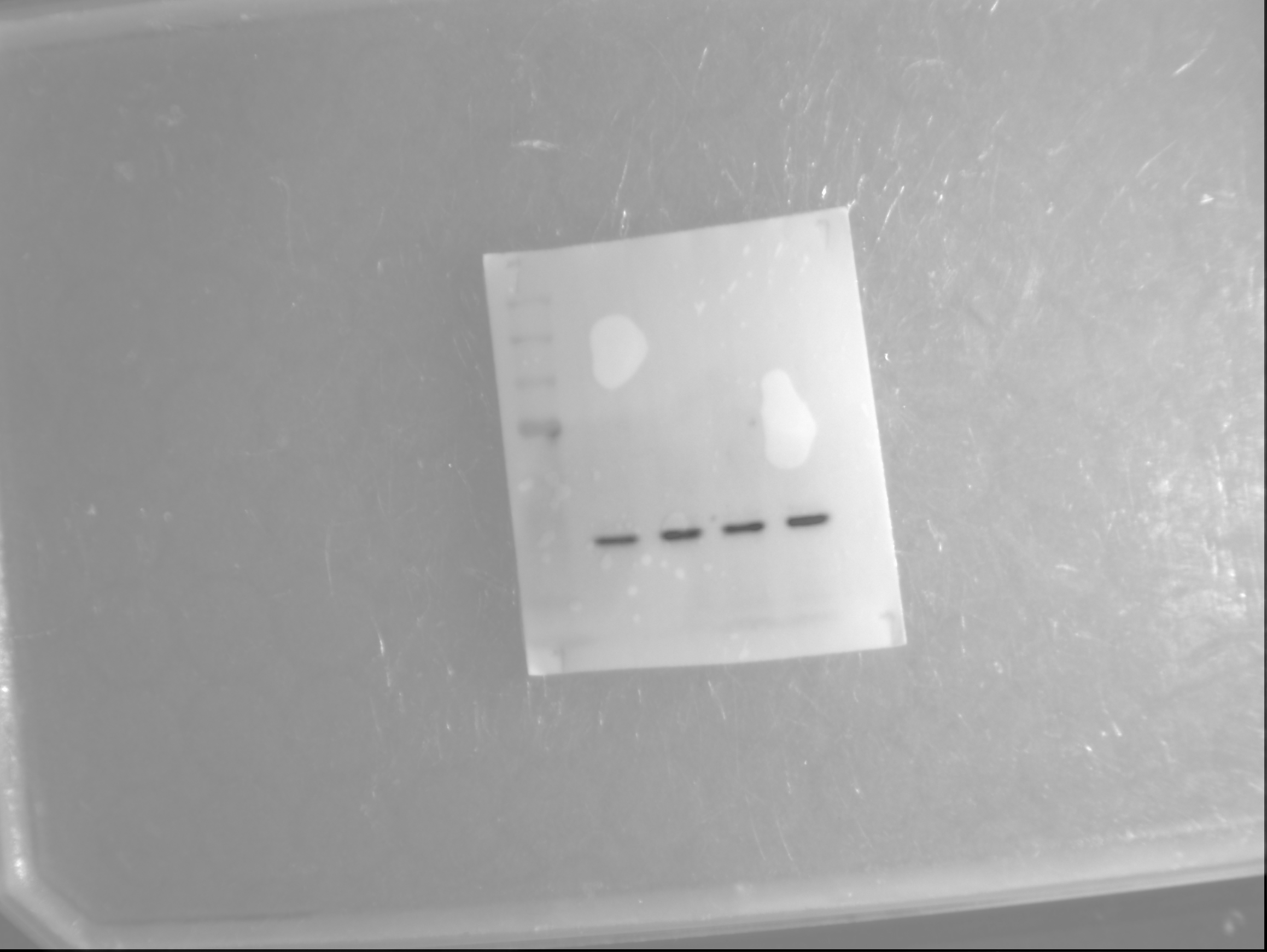

Supplement: S4 File — (ZIP) [file pone.0301540.s004.zip › S4 raw date 4/WB/TGF/pictur/3/tgf/2024-01-15-234232-image5.tif]

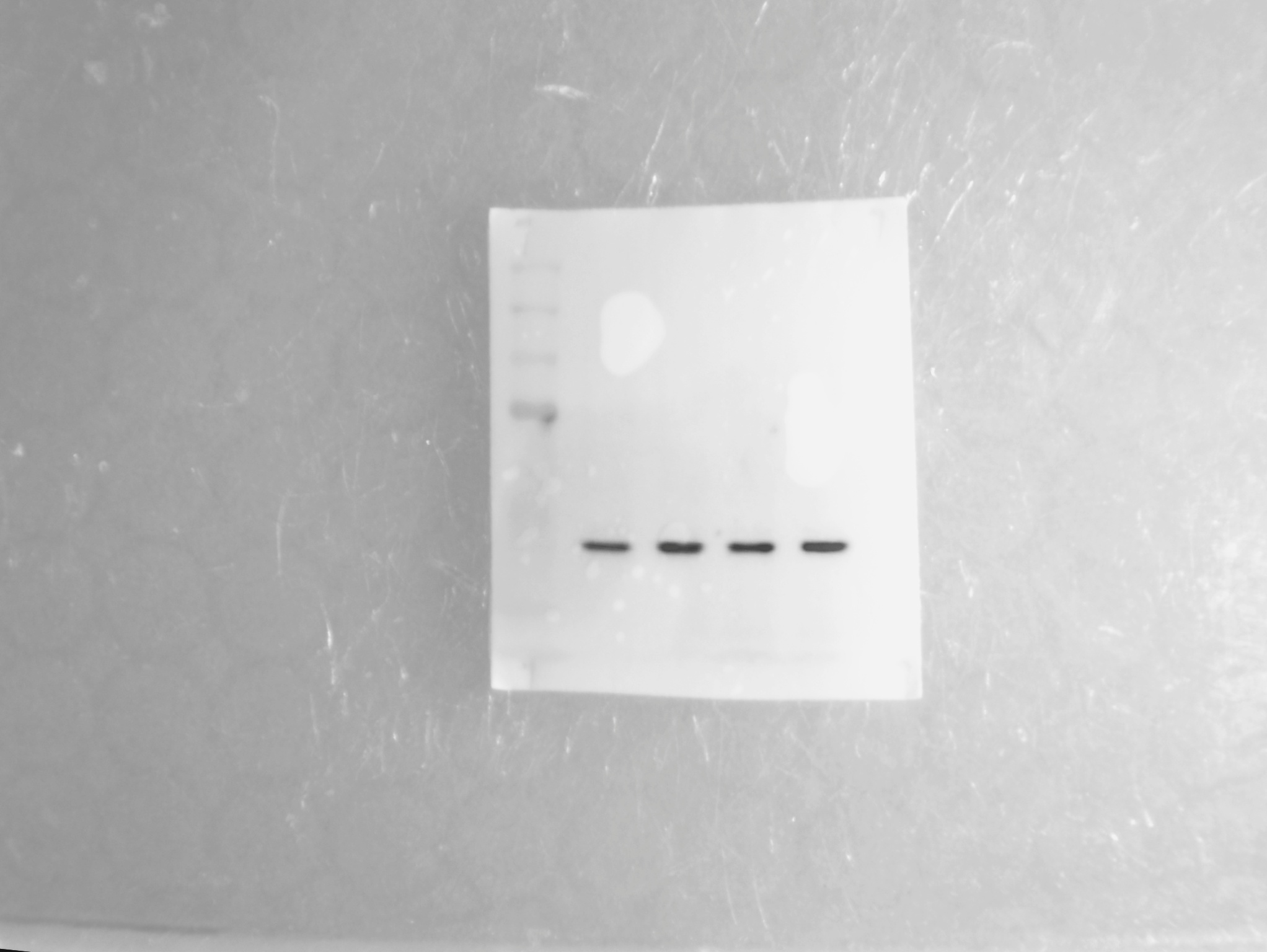

Supplement: S4 File — (ZIP) [file pone.0301540.s004.zip › S4 raw date 4/WB/TGF/pictur/3/tgf/2024-01-15-234232-image5_副本.jpg]

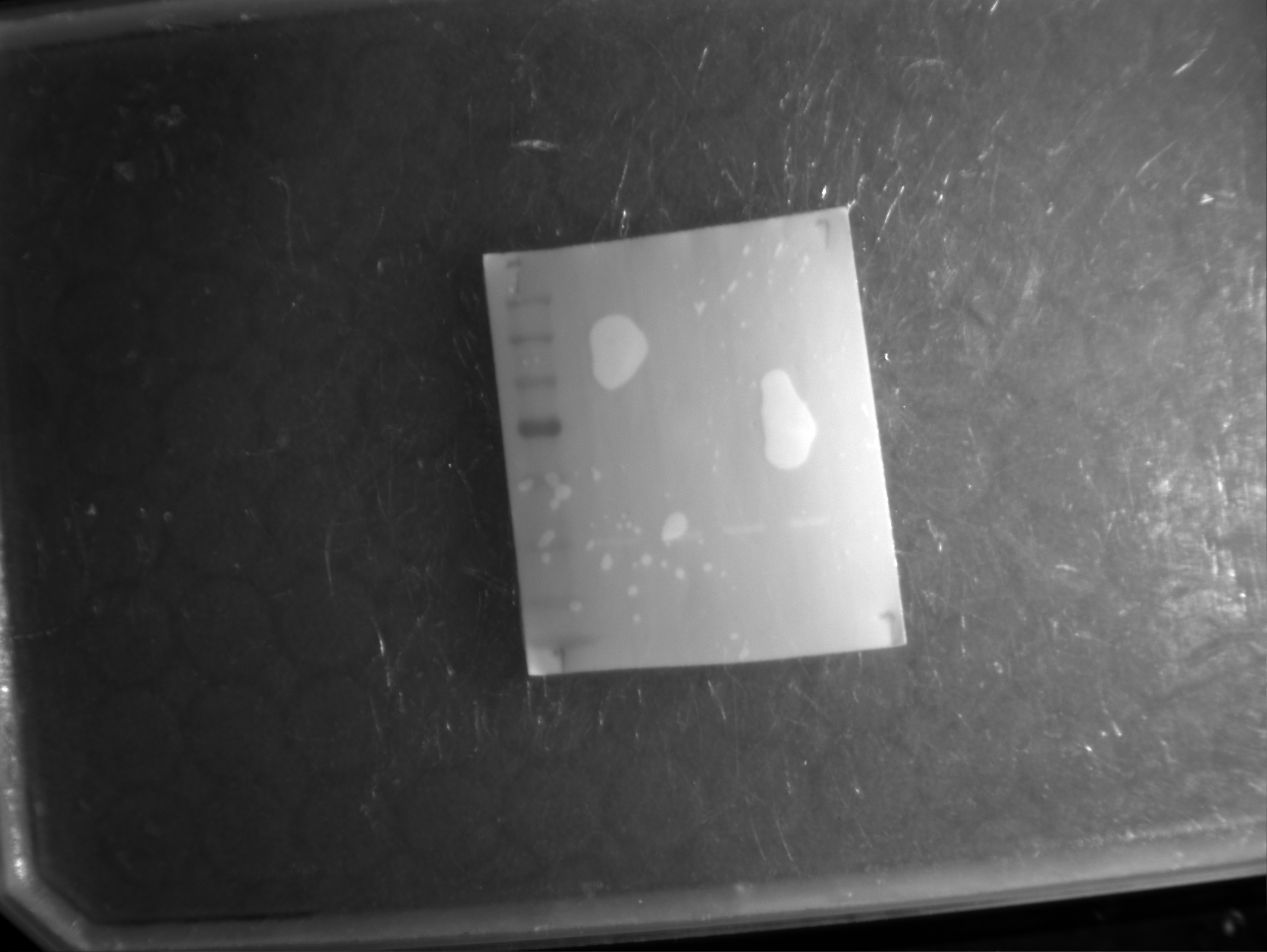

Supplement: S4 File — (ZIP) [file pone.0301540.s004.zip › S4 raw date 4/WB/TGF/pictur/3/tgf/2024-01-15-234236-image5-sub1-As-Displayed.tif]
